# Thiazolylcyanocyclopropanes: Novel Donor–Acceptor Cyclopropanes for Accessing Thiazole-Containing Targets

**DOI:** 10.3390/molecules30183767

**Published:** 2025-09-16

**Authors:** Emanuèl Bruno Savini, Edoardo Bandieri, Pietro Pecchini, Nicolò Santarelli, Luca Bernardi, Mariafrancesca Fochi

**Affiliations:** Department of Industrial Chemistry “Toso Montanari” and INSTM RU Bologna, Alma Mater Studiorum University of Bologna, V. P. Gobetti 85, 40129 Bologna, Italy; emanuelbruno.savini@unibo.it (E.B.S.); edoardo.bandieri@studio.unibo.it (E.B.); pietro.pecchini3@unibo.it (P.P.); nicolo.santarelli3@unibo.it (N.S.); luca.bernardi2@unibo.it (L.B.)

**Keywords:** donor–acceptor cyclopropanes, thiazoles, ring-strain release, cycloaddition, phase-transfer catalysis

## Abstract

Donor–acceptor (D–A) cyclopropanes are important precursors in the synthesis of complex molecules due to their bidentate character and high reactivity. Among them, cyclopropane-1,1-dicarbonitriles are less commonly reported in modern literature, primarily because of the high reactivity of the nitrile groups and their limited compatibility with metal-catalyzed processes, which is caused by the geometrical constraints imposed by the linear cyano substituents. While the cyano groups can be seen as a limitation, they also offer synthetic versatility by serving as handles for further functionalization. In this work, we performed a cycloaddition reaction with mercaptoacetaldehyde, leading to a new class of DA cyclopropanes bearing a thiazole moiety. This one-pot, two-step transformation requires only a single purification step. The resulting thiazolyl-substituted cyclopropanes were subjected to ring strain-release reactions, showing reactivity comparable to the parent cyclopropane-1,1-dicarbonitriles.

## 1. Introduction

Cyclopropane represents the smallest saturated cyclic structure. The C–C bonds in unsubstituted cyclopropane are relatively inert from a kinetic standpoint and, despite the significant ring strain, the molecule shows little tendency to open its cyclic framework [1].

However, this energy barrier is significantly reduced bearing vicinal donor (D) and acceptor (A) substituents within the three-membered ring. Through a synergistic “push–pull” effect, this substitution pattern promotes selective cleavage of the bond between the two substituents, formally generating a 1,3-zwitterionic intermediate. Consequently, D–A cyclopropanes have emerged as versatile building blocks in organic synthesis, serving as three-carbon synthetic equivalents for the construction of a wide variety of both acyclic and cyclic systems. D stands for electron-donating groups, such as OR, SR, NR_2_, CH_2_SiR_3_, aryl, alkyl, and cyclopropyl, whereas A represents electron-withdrawing groups, including CO_2_R, COR, CN, SO_2_Ph, NO_2_, and P(O)(OR)_2_. Detailed investigations by Werz and co-workers [2] have demonstrated that the nature of the donor and acceptor substituents exerts a profound influence on the kinetics of D–A cyclopropanes reactions. The synthesis of D–A cyclopropanes can be achieved through diverse strategies, as highlighted in a recent review [3].

**Scheme 1:**
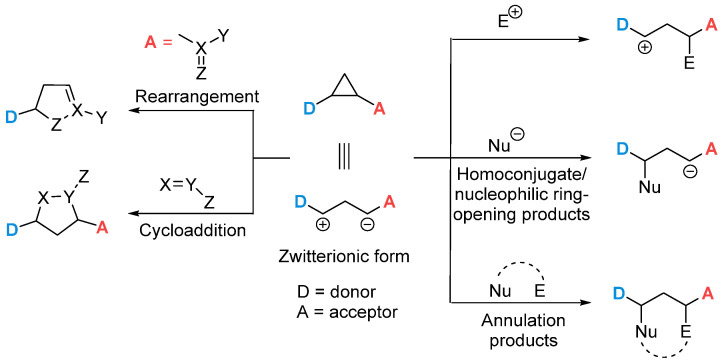
Zwitterionic relationship and reactivity of D–A cyclopropanes.

The remarkable reactivity and broad synthetic potential of D–A cyclopropanes account for the growing interest they have attracted among the scientific community. A plethora of synthetically valuable organic reactions has been reported, including rearrangements; cycloadditions with dipolarophiles, 1,3-dipoles, or dienes; and ring-opening processes with both nucleophiles and electrophiles, as well as with ambiphilic reagents, also in an enantioselective fashion. All these transformations take advantage of both the intrinsic ring strain and the pronounced bond polarization of D–A cyclopropanes (Scheme 1) [4,5,6,7,8,9,10,11,12,13,14,15,16,17,18].

Activation [4] of D–A cyclopropanes can be achieved through (i) thermal methods, (ii) Lewis or Brønsted acid/base catalysis, and (iii) low-valent transition metal catalysis [19,20]. Moreover, organocatalytic strategies have been recently reported, albeit less frequently [21,22,23,24,25].

The majority of reported studies on D–A cyclopropanes have focused on cyclopropyl-1,1-diesters [4], while cyclopropane-1,1-dicarbonitriles **1** have remained largely underexplored [21,22,24,25,26,27,28,29], primarily because of the high reactivity of the nitrile groups and their limited compatibility with metal-catalyzed processes, which is caused by the geometrical constraints imposed by the linear cyano substituents. Govaerts and Gómez-Suárez [30] very recently reported the nucleophilic ring-opening of cyclopropane-1,1-dicarbonitriles with alkyl amines, affording aminomalononitrile intermediates that were subsequently elaborated into structurally complex β-aminocarbonyl compounds. 2-Aroyl-substituted D−A cyclopropane-1,1-dicarbonitriles were recently used by Wang and Liu [31] for the DABCO-promoted (3+2) annulation with alkynoates, obtaining cyclopentenol derivatives, and by Wang [32] to access quinoxaline derivatives.

**Scheme 2:**
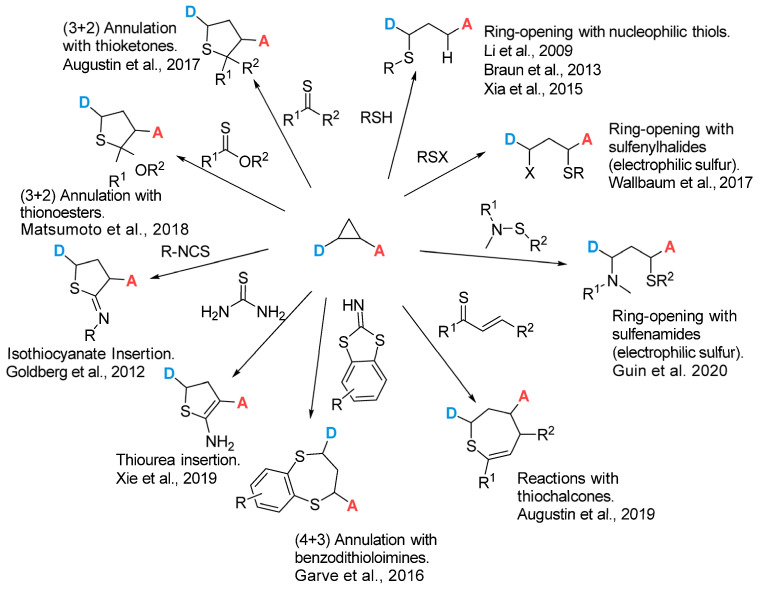
Representative transformations of donor–acceptor cyclopropanes leading to sulfur-containing compounds. Refs. [35,36,37,38,39,40,41,42,43,44,45].

Sulfur [33,34] has long been a favored heteroatom in the chemistry of D–A cyclopropanes, facilitating efficient access to sulfur-containing compounds of both cyclic and acyclic nature. Representative, non-exhaustive examples, are shown in Scheme 2. Ring-opening reactions of D–A cyclopropanes with sulfur nucleophiles—most prominently thiols—have been extensively investigated, including asymmetric variants [35,36,37]. Comparable reactivity is also observed with sulfur electrophiles, such as sulfenyl halides [38] and sulfenamides [39]. Beyond these processes, cycloaddition reactions provide efficient access to structurally diverse sulfur-containing heterocycles. For example, (3+2) annulations with thioketones [40] or thionoesters furnish a broad array of thiolanes [41], whereas reactions with isothiocyanates deliver thioimidates [42], and those with thioureas [43] afford 2-amino-4,5-dihydrothiophene derivatives. Higher-order annulations further expand this chemistry: (4+3) cycloadditions with amphiphilic benzodithioloimines, acting as surrogates for orthobisthioquinones, yield benzo-fused dithiepines [44], while reactions with thiochalcones [45] enable the synthesis of unsaturated tetrahydrothiepines.

**Scheme 3:**
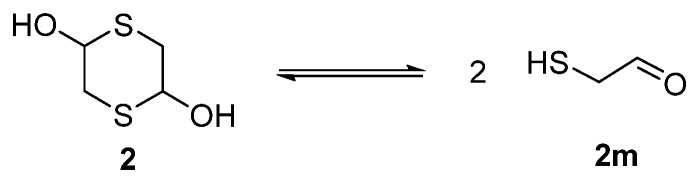
Formation of 2-mercaptoacetaldehyde **2m** from 1,4-dithiane-2,5-diol **2**.

The formation of six-membered sulfur-containing heterocyclics can be achieved via (3+3) annulation reactions of D−A cyclopropanes with mercaptoacetaldehyde **2m**, which can conveniently be generated in situ from its commercially available dimer, 1,4-dithiane-2,5-diol **2** (Scheme 3), serving as a bifunctional substrate to trigger cascade reactions. 1,4-Dithiane-2,5-diol **2** is a highly versatile synthetic precursor [46], extensively utilized in (3+2) annulations for the synthesis of various tetrahydrothiophenes, dihydrothiophenes, and thiophenes, as well as in (3+3) annulations involving azomethine imines.

**Scheme 4:**
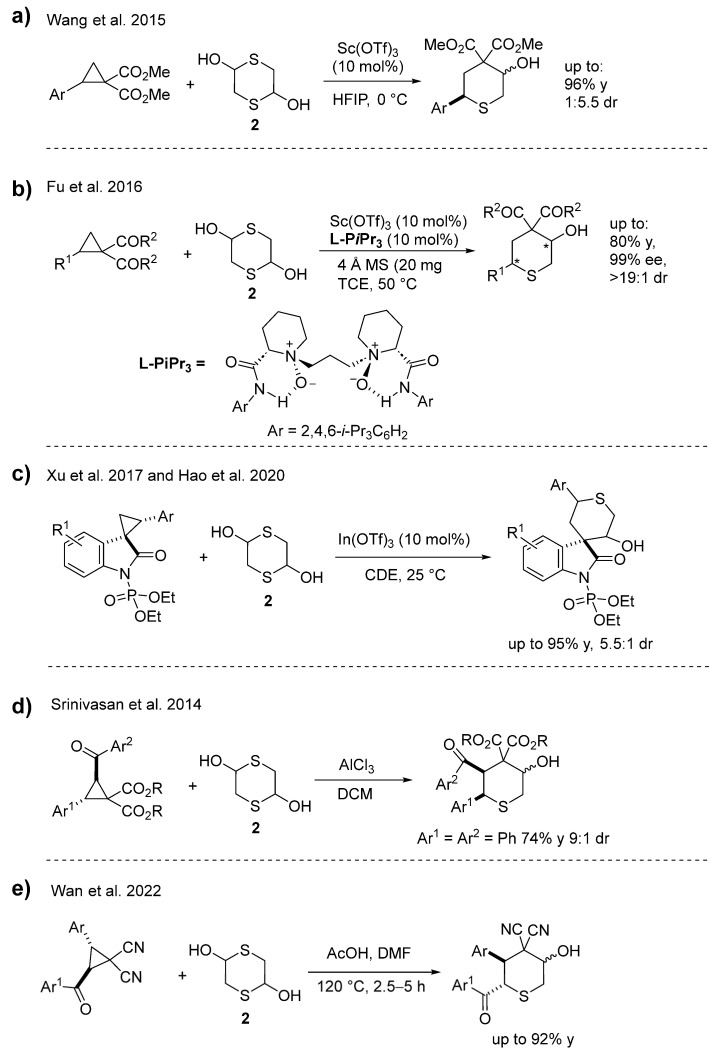
D–A cyclopropanes reactions with mercaptoacetaldehyde reported in the literature: (**a**) Ref. [47]; (**b**) Ref. [48]; (**c**) Refs. [49,50]; (**d**) Ref. [51]; (**e**) Ref. [52]. * = Asymmetic center.

Few studies in recent years have explored the reactivity of D–A cyclopropanes with mercaptoacetaldehyde. Wang et al. [47] have examined the reactivity of mercaptoacetaldehyde with D–A cyclopropanes-1,1-diesters, exploiting the activation of Lewis acid as Sc(OTf)_3_ to achieve the formation of polyfunctionalized tetrahydrothiopyranols in good yields (Scheme 4a). Fu et al. [48] developed an asymmetric version of this (3+3) annulation reaction with excellent diastereoselectivities and enantioselectivities in the presence of an *N*,*N*’-dioxide-Sc(III) complex as the catalyst (Scheme 4b). Later, Xu et al. [49] reported a fascinating catalytic enantioselective (3+3) annulation using spirocyclic D–A cyclopropanes, activated by installing an electron-withdrawing *N*-protecting group, with both aldonitrones and ketonitrones. This study also highlighted an example in which a spirocyclic D–A cyclopropane reacted with mercaptoacetaldehyde, leading to the formation of a spirocyclic tetrahydrothiopyranol in excellent yield, exploiting In(OTf)_3_ as a catalyst. In 2020, Hao et al. reported, in detail, the In(OTf)_3_-catalyzed (3+3) annulation of spirocyclopropyl oxindoles and 1,4-dithiane-2,5-diol **2** to assemble spiro-tetrahydrothiopyrans [50] (Scheme 4c).

Srinivasan et al. [51] studied an AlCl_3_-catalyzed (3+3) annulation of mercaptoacetaldehyde with aroyl-substituted D–A cyclopropanes leading to the formation of tetrahydrothiopyranols (Scheme 4d), and, in 2022, Wan et al. [52] disclosed an efficient acetic-acid-mediated regioselective (3+3) cycloaddition of aroyl-substituted cyclopropane-1,1-dicarbonitriles with mercaptoacetaldehyde for the synthesis of tetrahydrothiopyranols in a highly stereoselective way (Scheme 4e).

**Scheme 5:**
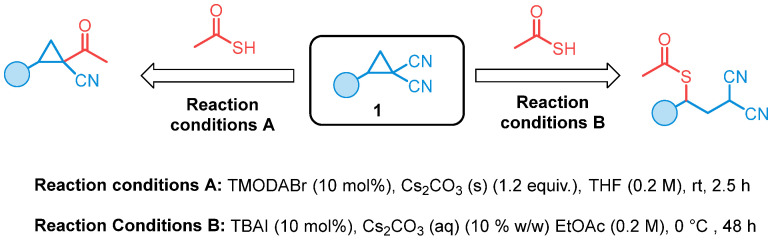
Divergent reactivity of cyclopropane-1,1-dicarbonitriles **1** with thioacetic acid.

Building on our extensive experience with phase transfer catalysis (PTC) [53,54,55,56,57,58,59,60] and inspired by the synthetic potential of cyclopropane-1,1-dicarbonitriles **1**, we embarked a few years ago on an investigation of their reactivity with sulfur nucleophiles under PTC conditions. A detailed study of cyclopropane-1,1-dicarbonitriles **1** with thioacetic acid as the nucleophile revealed a previously unreported decyanation–acetylation pathway, affording 1-acetyl-1-cyanocyclopropanes alongside the more conventional ring-opening products [61]. Systematic screening of PTCs, solvents, and temperatures led to the development of two complementary protocols, allowing for excellent control over product distribution (Scheme 5).

Following up on our previous findings with cyclopropane-1,1-dicarbonitriles **1**, we directed our focus toward their reactivity with mercaptoacetaldehyde **2m** under phase transfer catalysis (PTC).

## 2. Results and Discussion

**Scheme 6:**
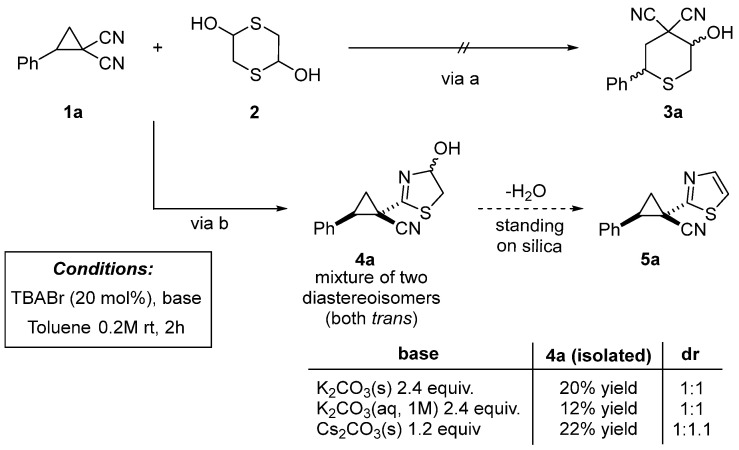
Reaction of cyclopropane-1,1-dicarbonitrile **1a** with 1,4-dithiane-2,5-diol **2**.

We started our investigation using our typical PTC conditions (tetrabutylammonium bromide (TBABr) as the phase-transfer catalyst, K_2_CO_3_ as the solid base, and toluene as the solvent). The (3+3) annulation of cyclopropane-1,1-dicarbonitrile **1a** with 1,4-dithiane-2,5-diol **2** was expected to yield the corresponding tetrahydrothiopyranol **3a**, in agreement with prior reports (Scheme 6, via a). Unexpectedly, the reaction yielded a novel cyclopropane derivative **4a**, arising from the cycloaddition of mercaptoacetaldehyde at one of the two CN functionalities of the substrate. (Scheme 6, via b). The hydroxythiazoline derivative **4a** was isolated in 20% yield as an approximately 1:1 mixture of two diastereoisomers, both *trans* (see below) at the cyclopropane moiety, with different configurations at the alcoholic carbon. The use of an aqueous base (K_2_CO_3_, 1 M) yielded product **4a** in significantly lower yield, and, likewise, under these conditions no trace of product **3a** was observed. On the other hand, the use of solid Cs_2_CO_3_ slightly improved the yield.

We observed that product **4a** was quite unstable on silica gel, yielding a small amount of the corresponding thiazolyl derivative **5a** during column chromatography. Although the hydroxythiazoline derivative **4a** could be isolated as a 1:1 mixture of two *trans*-diastereoisomers and completely characterized (see Supplementary Materials), we established a direct one-pot procedure (see below) to achieve the complete dehydration of **4a** to the corresponding thiazole **5a**, thereby avoiding a tedious and lengthy purification process.

Thiazoles are important five-membered heterocyclic systems that are widely found in pharmaceutical compounds and exhibit a broad spectrum of biological and pharmacological activities [62,63,64,65,66]. Thiazoles are most commonly prepared by Hantzsch synthesis involving the reaction of α-halo ketones with substituted thioamides [67]. In addition, a limited number of recent reports have described the preparation of thiazolyl derivatives from 1,4-dithiane-2,5-diol **2** and various nitrile derivatives [68,69].

It is important to point out that a double addition to both nitrile groups of D–A cyclopropane **1a** has never been observed, in agreement with previous reports on systems bearing two cyano groups, of which typically only one undergoes reaction [68,69] and that product **3a** was never detected in the crude reaction mixture.

Having identified hydroxythiazoline derivative **4a** as the only product, we started an optimization process of the reaction conditions, varying the catalyst, solvent, temperature, and reaction time. Since the obtained yields with potassium and caesium carbonate in the first attempts reported in Scheme 6 were similar, screening was initially performed using K_2_CO_3_. To facilitate the analysis of crude reaction mixtures from the screening process, 2-(4-fluorophenyl)cyclopropane-1,1-dicarbonitrile **1b** was selected as the model compound, in addition to **1a**, due to its compatibility with direct analysis by ^19^F NMR spectroscopy using α,α,α-trifluorotoluene (TFT) as an internal standard (Table 1).

First, various ammonium salts were screened as PT catalysts, and, as reported in entry 3, trimethyloctadecylammonium bromide (TMODAB) yielded product **4b** in 44% yield, and it was selected as the optimal catalyst. The obtainable yield of **4b** can be increased to 53% by reducing the amount of K_2_CO_3_ to 1.2 equivalent (entry 4). Subsequently, a range of solvents was evaluated, including alcohols (ethanol and *n*-butanol), ethyl acetate, acetonitrile, chlorinated solvents (dichloromethane and 1,2-dichloroethane), and various ethers (entries 5–14). Cyclopentyl methyl ether (CPME entry 15) afforded the highest yield among the solvents tested and was, consequently, selected for subsequent screening.

Concerning the base, solid caesium carbonate showed slightly better yields compared to solid potassium carbonate (compare entries 14 and 15). Stronger bases such as potassium hydroxide or potassium *tert*-butoxyde gave rise to highly complex crude mixtures and afforded significantly lower yields of **4b** (16% and 23%, respectively, not included in Table 1). Additionally, an increased yield was observed when the reaction is performed in dry solvent and in the presence of 3 Å molecular sieves, thus confirming the detrimental effect of water on the reaction (entry 16). After complete conversion of the starting material, prolonged standing in the reaction conditions leads to a decrease in yield (see entry 17), presumably due to dehydration to **5b** and other side reactions involving the hydroxythiazoline derivative. A few experiments were conducted to evaluate the effect of different reaction temperatures (0 °C and 40 °C, not reported in Table 1). Lowering the temperature only resulted in a longer reaction time, while increasing it led to a crude mixture with more byproducts.

The diastereoselectivity of the reaction was not affected by variations in PT catalysts, solvent, or temperature, consistently affording a 1:1 mixture of the two *trans*-diastereomers of **4b**. The corresponding *cis*-diastereomers were observed in amounts lower than 5%, as determined by crude ^1^H NMR analysis.

Therefore, the optimized condition entailed the utilization of cyclopropane-1,1-dicarbonitriles **1** (0.1 mmol), 1,4-dithiane-2,5-diol **2** (0.1 mmol), tetrametiloctadecylammonium bromide (TMODAB) as PT catalyst (20 mol%), solid Cs_2_CO_3_ (1.2 equiv.) as the base, and 3 Å molecular sieves (500 mg/mmol, powder preactivated in a microwave oven and stored under argon) in dry cyclopentyl methyl ether (CPME) at room temperature for 1 h.

As previously noted, the hydroxythiazoline derivatives **4** exhibit limited stability under the reaction conditions and on silica. Therefore, the reaction time for each substrate was individually adjusted based on TLC monitoring of the conversion. In addition, a one-pot dehydration protocol for converting hydroxythiazoline derivatives **4** into the corresponding thiazoles **5** was developed and optimized. Several in situ dehydration protocols were evaluated: the reaction between **1a** and **2** was carried out under the optimized conditions described above and, upon complete conversion of **1a**, the dehydrating agent was added and the reaction mixture was heated to 70 °C. Various dehydrating agents—such as tosyl chloride/triethylamine, mesyl chloride/DIPEA, sulfur trioxide–pyridine complex/DIPEA, and the Burgess reagent—were tested, and the outcomes of these experiments are summarized in Table 2.

The Burgess reagent (1-methoxy-*N*-triethylammoniosulfonyl-methanimidate), a carbamate-based internal salt known for promoting β-hydrogen *syn* elimination of secondary and tertiary alcohols [70], afforded the desired dehydration in high yield (entry 5) compared to the other methodology tested (entries 1–4) and was accordingly chosen for the second step of the synthetic sequence. Furthermore, it was observed that dehydration of the mixture of the two *trans*-diastereoisomers of **4a** afforded a mixture of the thiazoles *trans*-**5a** and *cis*-**6a**, in ratios that varied depending on the specific dehydration conditions employed. This outcome is likely attributable to a ring-opening/closing process promoted by the reaction conditions. The diastereomeric ratio between **5a** and **6a** was largely preserved during the dehydration step employing the Burgess reagent, maintaining an excellent ratio of 20:1 in favor of **5a**. As shown in Table 2, the yields of **5a** and **6a** increase with the amount of Burgess reagent (entries 5–8), reaching an optimal value of 78% in the presence of four equivalents (entry 7), with a satisfactory ratio of 14:1 in favor of **5a** (corresponding to a 94% of **5a**, starting from >95% diastereomeric excess of **4a**).

**Scheme 7:**
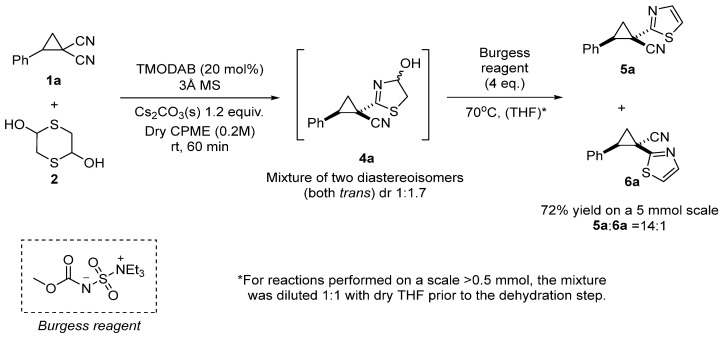
Optimized conditions for the one-pot reaction.

Scalability of the dehydration step beyond the 0.5 mmol scale was improved by diluting the reaction mixture with an equal volume of dry THF relative to the reaction solvent (CPME) prior to the addition of the Burgess reagent, which proved effective in minimizing clumping and stirring issues commonly encountered at larger scale. Both the yield and diastereoselectivity in scaled-up reactions were comparable to—or only slightly lower than—those obtained on a small scale. Indeed, the one-pot reaction was performed on a 5 mmol scale (840 mg of **1a**), affording **5a** in 72% yield and 14:1 dr (Scheme 7).

**Scheme 8:**
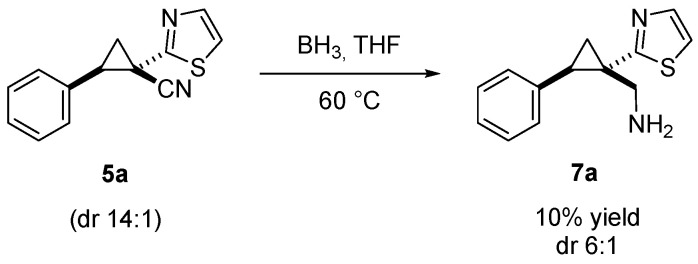
Reduction of the nitrile group of **5a**.

The relative configuration of the major product **5a** was investigated through a series of NOE experiments (see Supplementary Materials); however, these did not provide unambiguous results. Consequently, the nitrile group of **5a** was reduced using a borane–tetrahydrofuran complex, obtaining the corresponding methanamine derivative **7a** (10% yield, retaining a 6:1 dr, reaction conditions were not optimized) (Scheme 8). NOE experiments were repeated on this compound, revealing a *trans*-relative configuration of the main diastereoisomer (see Supplementary Materials).

**Scheme 9:**
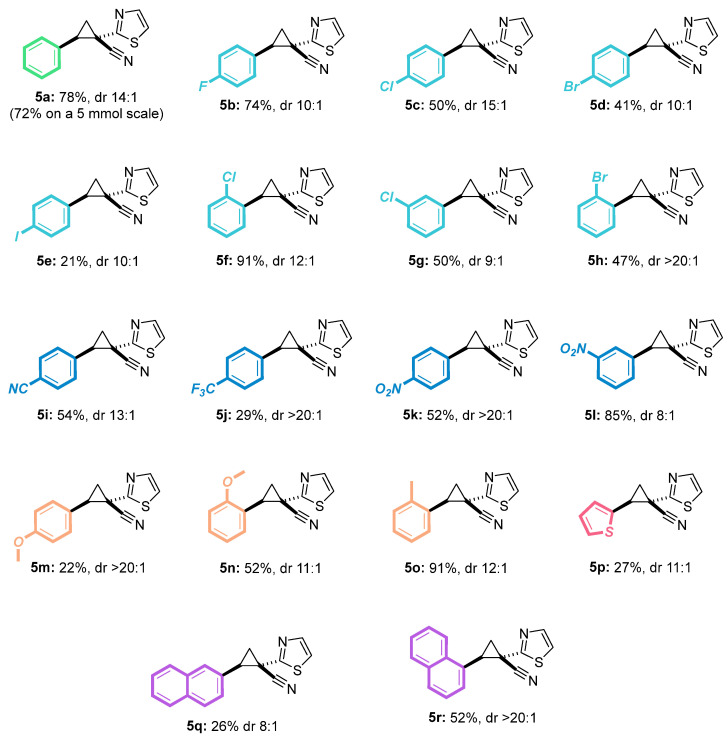
Reaction scope (isolated yields, dr determined by ^1^H NMR on the crude reaction mixture, structure of the major *trans* product).

With the optimal conditions in hand for both the reaction steps, the substrate scope was then evaluated (Scheme 9).

The optimized procedure was successfully applied to all cyclopropanes **1a**–**1r**, enabling the synthesis of thiazolyl derivatives **5a**–**5r** irrespective of the electronic nature—donating or withdrawing—(for example, the 2-chlorophenyl derivative **5f** was obtained in a 91% yield, as well as the 2-methylphenyl derivative **5o**, also in a 91% yield) and the position of the substituent on the aromatic ring (for example, the 4-bromophenyl derivative **5d** was obtained in a 41% yield and the 2-bromophenyl derivative **5h** in a 47% yield) in good or very good yields. Successful examples include cyclopropane derivatives bearing bulky groups such as naphthalene (**5q**—26% yield and **5r**—52% yield), as well as heteroaromatic rings like 2-thiophene (**5p**—27% yield). All thiazoles **5a**–**5r** were obtained with high diastereomeric ratio up to >20:1, and the *trans*-configuration determined for **5a** was accordingly assigned to all other analogues in the series.

**Scheme 10:**
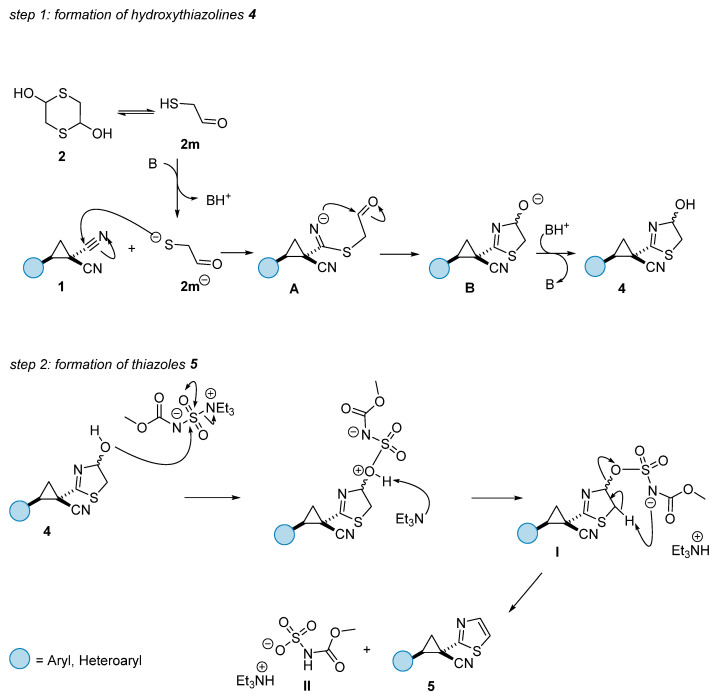
Plausible mechanism for the one-pot procedure: synthesis of hydroxythiazolines **4** (step 1) and their subsequent dehydration to thiazolyl derivatives **5** (step 2).

A plausible mechanism for the synthesis of hydroxythiazolines **4**, supported by a DFT calculation (see Supplementary Materials), is proposed in Scheme 10. Mercaptoacetaldehyde monomer (**2m**), generated in situ by **2** and deprotonated by the base, attacks the less-hindered -CN group *trans* to the aromatic substituent at C2, forming intermediate **A**. Subsequently, intramolecular nucleophilic attack of the nitrogen atom on the aldehyde carbonyl group leads to cyclization, yielding the ring-closed product **B**, which upon protonation affords hydroxythiazolines **4**. The second step, promoted by the Burgess reagent, follows the classical mechanism reported in the literature [71,72], involving the formation of an alcohol-derived leaving group, triethylammonium *N*-carboalkoxysulfamates **I**, which smoothly decomposes upon heating to afford thiazolyl derivatives **5** and the water-soluble salt **II**.

As previously reported, the tetrahydrothiopyranol **3a**, which would formally arise from ring-opening of **1a** followed by intramolecular cyclization, was never observed. This outcome can likely be ascribed to the fact that, under the employed basic conditions, the bond between the two cyclopropane carbons bearing the donor and acceptor substituents is not sufficiently weakened. In other words, the push–pull activation exerted by the vicinal donor–acceptor substitution does not provide an adequate driving force for selective cleavage of this bond, thereby preventing formation of **3a** in PTC conditions.

**Scheme 11:**
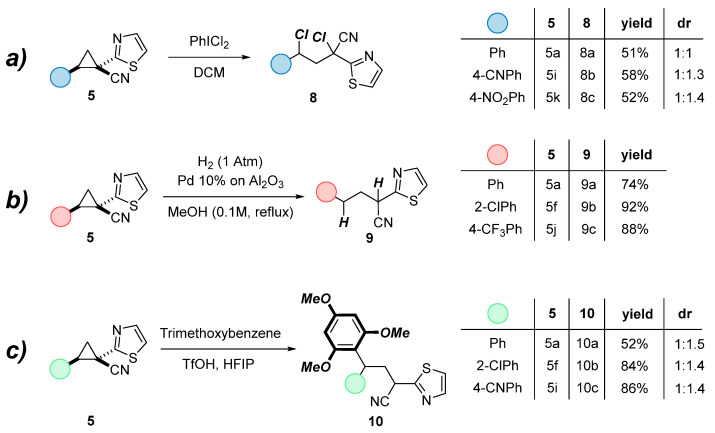
Structure modification of thiazolyl-substituted D–A cyclopropanes **5**: (**a**) dichlorination; (**b**) reductive ring opening; (**c**) arylation.

Finally, a series of classical cyclopropane ring-opening reactions was carried out on thiazolyl derivatives **5** to assess whether they retained the characteristic reactivity of these systems. It is well established that D–A cyclopropanes undergo ring opening in the presence of phenyl iodine dichloride to give 1,3-dichlorinated compounds [73]. This transformation was extended to thiazolyl-substituted D–A cyclopropanes **5**, which reacted under the same conditions to afford the corresponding dichlorinated products **8** in good yields as a roughly 1:1 mixture of diastereoisomers (Scheme 11a). Reductive ring opening through hydrogenolysis, in the presence of Pd/alumina at atmospheric pressure, of the thiazoles **5** is also possible, leading to thiazolyl butanenitriles **9** in excellent yields (Scheme 11b), whereas Friedel-Craft-type arylation with 1,3-5-trimetoxybenzene in hexafluoroisopropanol (HFIP) furnished the corresponding arylated compounds **10** as a 1:1.5 or 1:1.4 mixture of diastereoisomers (Scheme 11c).

## 3. Materials and Methods

### 3.1. General Methods

NMR analyses were conducted using the following instruments: Bruker 600 MHz (Billerica, MA, USA, all nuclei), Varian Inova 600 MHz (Palo Alto, CA, USA, ^1^H, ^13^C, NOE, and 2D), Varian MR 400 MHz (^1^H, ^13^C, and ^19^F), Varian Mercury 300 MHz (^1^H, ^13^C, and ^19^F), and Varian Mercury 400 MHz (^1^H, ^13^C, and ^19^F). Chemical shifts (δ) are reported in ppm relative to the residual solvent signals [74] for ^1^H and ^13^C NMR; ^19^F NMR spectra were referenced to α,α,α-trifluorotoluene (PhCF_3_, TFT) at δ = −63.72 ppm. Signal patterns are indicated as follows: bs, broad singlet; s, singlet; d, doublet; t, triplet; q, quartet; and m, multiplet. Coupling constants (*J*) are given in Hertz (Hz). ^13^C NMR were acquired with ^1^H broad-band decoupled mode. For all products **5** obtained in diastereomeric mixtures, the reported NMR signals refer to the major diastereoisomer. 

All HPLC-MS analyses were performed using an Agilent Infinity II 1260 system (Santa Clara, CA, USA) equipped with a DAD and ESI/SQ detector. Unless otherwise specified, separations were carried out using an Infinity Lab Poroshell 120 EC-C18 (Agilen), 4.6 × 150 mm, 2.7 µm with a gradient elution from 5% to 95% acetonitrile in water; the eluent contained 0.1% formic acid to enhance ionization.

GC-MS analyses were performed on an Agilent 8890 GC system coupled to a 5977C mass-selective detector (MSD). Helium was used as the carrier gas at a constant flow rate of 1.2 mL/min. The oven temperature was programmed from 60 °C to 250 °C. The ionization source operated in EI mode at 70 eV and 230 °C, while the quadrupole was maintained at 150 °C, scanning in the *m*/*z* range of 50–550.

High-resolution mass spectrometry (HRMS) analyses were performed using two ionization techniques. Electrospray ionization (ESI) HRMS spectra were acquired on a Waters Xevo G2-XS QT (Milford, MA, USA) of a spectrometer operated in the reflectron mode, using acetonitrile containing 0.1% formic acid as the mobile phase. Samples were introduced via the Waters Aquicity H Plus UPLC autosampler in direct infusion mode. Matrix-assisted laser desorption/ionization (MALDI) HRMS spectra were obtained on a Waters Synapt MALDI Q-TOF G2S spectrometer, also operated in the reflectron mode, employing α-cyano-4-hydroxycinnamic acid (4-HCCA, Sigma-Aldrich, St. Louis, MO, USA) as the ionization matrix.

FTIR spectra were acquired on a Bruker Alpha II, operating in the ATR mode.

NMR yields were determined using α,α,α-trifluorotoluene, trimethoxybenzene, dinitrobenzene, dibromomethane, or ethylene carbonate as internal quantification standards.

### 3.2. Materials

Analytical-grade solvents and commercially available reagents were used as received, unless otherwise stated. Dry THF was obtained by distillation from Na/benzophenone before use. Dry CPME and DCM were prepared by storing them over microwave-activated 3 Å molecular sieves. Solvent dryness was measured with a Karl–Fischer system, model Metrohm Eco Coulometer.

Chromatographic separations were carried out using a Büchi Chromatography system Pure C-815 Flash (Flawil, Switzerland) (FlashPure EcoFlex cartridge (4 to 40 g), 50 µm irregular) and by flash column chromatography using Silica 60 M (0.04–0.063 mm) Macherey–Nagel (Düren, Germany). Thin-layer chromatography analyses were performed using Alumgram Xtra SIL G UV254 plates from Macherey–Nagel.

**Scheme 12:**
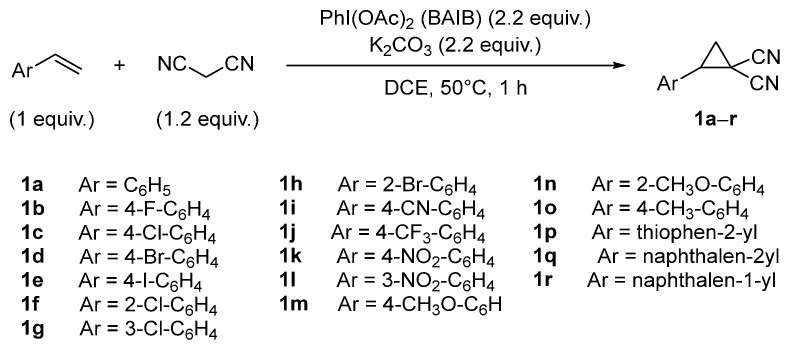
Synthesis of D–A cyclopropanes **1a**–**1r**.

D–A cyclopropanes **1a**–**1r** (Scheme 12) were obtained from the corresponding styrene derivatives and malononitrile following a procedure in the literature using bisacetoxyiodobenzene (BAIB) and K_2_CO_3_ [75] (see Supplementary Materials for ^1^H NMR spectra). The corresponding styrene derivatives, if not commercially available, were obtained by Wittig reaction from the corresponding aldehydes.

### 3.3. Synthesis of 1-(4-Hydroxy-4,5-dihydrothiazol-2-yl)-2-phenylcyclopropane-1-carbonitrile (***4a***)

To an appropriately sized vial was added 2-phenylcyclopropane-1,1-dicarbonitrile **1a** (1 equiv., 0.1 mmol, 16.8 mg); 1,4-dithiane-2,5-diol **2** (1 equiv., 0.1 mmol, 15.2 mg); caesium carbonate (1.2 equiv., 1.2 mmol, 39 mg); trimethyl(octadecyl)ammonium bromide (0.2 equiv., 0.2 mmol, 7.8 mg); and microwave-activated 3 Å molecular sieves (500 mg/mmol of **1a**, 50mg). The reagents were suspended in anhydrous cyclopentyl methyl ether (5 mL/mmol of **1**, 0.50 mL), and the reaction mixture was stirred vigorously at room temperature (1500 rpm) using a magnetic stirrer. The progress of the reaction was monitored by TLC (25% EtOAc in petroleum ether) until complete consumption of the starting material was observed. Reaction crude was evaporated under reduced pressure and purified by column chromatography (diethyl ether), and product **4a** was obtained in a 76% yield (18.6 mg) as a 1:1.7 mixture of two *trans*-diastereoisomers at the -OH carbon. The *trans*:*cis* diastereomeric ratio was determined to be >20:1 by crude ^1^H-NMR analysis.

^1^H NMR (600 MHz, CDCl_3_) major *trans*-diasteroisomer δ 7.38–7.26 (m, 5H), 6.11 (m, 1H), 3.61 (dd, *J* = 12.1, 7.0 Hz, 1H), 3.38 (dd, *J* = 12.1, 4.6 Hz, 1H), 3.26–3.21 (m, 1H), 2.95 (broad d, *J* = 4.7 Hz, 1H), 2.31–2.26 (m, 1H), and 2.23–2.19 (m, 1H).

The ^1^H NMR signals of the minor *trans*-diastereoisomer largely overlap with those of the major *trans*-diastereoisomer; however, the two OH signals can be clearly distinguished at different chemical shifts, as follows: 2.95 ppm (broad d, *J* = 4.7 Hz, 1H) for the major and 2.98 ppm (broad d, *J* = 4.5 Hz, 1H) for the minor isomer in a ratio 1:1.7 (See supplementary materials).

^13^C NMR (151 MHz, CDCl_3_) δ Major (M) and minor (m): 170.81 (m), 170.79 (M),133.28 (m), 133.22 (M), 128.8 (M+m), 128.5 (M+m), 128.2 (M+m), 117.48 (m), 117.46 (M), 98.8 (M+m), 40.94 (M), 40.91 (m), 37.22 (M), 36.97 (m), 24.49 (M), 24.10 (m), and 23.9 (M+m).

HRMS (MALDI-rTOF) *m*/*z* [M+Na]^+^ calc.: 267.0563 found: 267.0568.

### 3.4. General One-Pot Procedure for the Synthesis of Thiazoles ***5***

To an appropriately sized vial were added 2-arylcyclopropane-1,1-dicarbonitrile **1** (1.0 equiv.), 1,4-dithiane-2,5-diol **2** (1 equiv.), caesium carbonate (1 equiv.), trimethyl(octadecyl)ammonium bromide (0.2 equiv.), and microwave-activated 3 Å molecular sieves (500 mg/mmol of **1**). The reagents were suspended in anhydrous cyclopentyl methyl ether (5 mL/mmol of **1**), and the reaction mixture was stirred vigorously at room temperature (1500 rpm) using a magnetic stirrer. The progress of the reaction was monitored by TLC (25% EtOAc in petroleum ether) until complete consumption of the starting material was observed. At this point, anhydrous tetrahydrofuran (5 mL/mmol of **1**) and Burgess reagent (4.0 equiv.) were added (for reaction in smaller scale than 0.2 mmol dilution of the reaction mixture with THF is not necessary). The reaction mixture was then heated to 75 °C under continuous stirring for 30 min to promote dehydration. Complete conversion of the hydroxythiazoline **4** was confirmed by TLC (100% diethyl ether as eluent on SiO_2_). The crude reaction mixture was diluted with dichloromethane and adsorbed onto silica gel. After removal of solvents under reduced pressure, a free-flowing powder was obtained. Purification by flash column chromatography (40% diethyl ether in cyclohexane) yielded thiazoles **5** as a mixture of diastereoisomers, with a diastereomeric ratio unchanged from the one observed prior to chromatography.

*Trans*-2-phenyl-1-(thiazol-2-yl)cyclopropane-1-carbonitrile (**5a**)

Following the general procedure and using cyclopropane **1a** (16.8 mg), product **5a** was obtained in a 78% yield (17.7 mg) after chromatographic purification on silica gel (40% diethyl ether in cyclohexane, R_f_: 0.43) as a yellow oil. The diastereomeric ratio was determined to be 14:1 by crude ^1^H-NMR analysis. The reaction was repeated employing 840 mg (5 mmol) of **1a**, obtaining compound **5a** in a 72% yield.

IR (ATR, cm^−1^): 3100–3000 (CH, m), 2240 (CN, s), and 1602 (C=C, m). NMR signals refer to the major *trans*-diastereoisomer. ^1^H NMR (400 MHz, CDCl_3_) δ 7.71 (d, *J* = 3.3 Hz, 1H), 7.44–7.31 (m, 5H), 7.27 (d, *J* = 3.3 Hz, 1H), 3.34 (t, *J* = 8.7 Hz, 1H), 2.45 (dd, *J* = 9.1, 5.5 Hz, 1H), and 2.34 (dd, *J* = 8.2, 5.5 Hz, 1H). ^13^C NMR (151 MHz, CDCl_3_) δ 165.4, 142.9, 133.9, 129.1, 128.75, 128.3, 128.2, 128.1, 118.9, 118.5, 37.7, 24.7, and 23.4. HRMS (MALDI-rTOF) *m*/*z* [M + H]^+^ calc.: 227.0638, found: 227.0645.

The *trans*-relative configuration was determined by NOE experiments (see Supplementary Materials).

*Trans*-(4-fluorophenyl)-1-(thiazol-2-yl)cyclopropane-1-carbonitrile (**5b**)

Following the general procedure and using cyclopropane **1b** (37.2 mg), product **5b** was obtained in 48% yield (23.6 mg) after chromatographic purification on silica gel (40% diethyl ether in cyclohexane, R_f_: 0.40) as a yellow oil. The diastereomeric ratio was determined to be 10:1 by crude ^1^H-NMR analysis. NMR signals refer to the major *trans*-diastereoisomer. ^1^H NMR (600 MHz, CDCl_3_) δ 7.68 (d, *J* = 3.3 Hz, 1H), 7.33–7.24 (m, 2H), 7.10–7.02 (m, 2H), 3.30 (t, *J* = 8.6 Hz, 1H), 2.42 (dd, *J* = 9.1, 5.6 Hz, 1H), and 2.27 (dd, *J* = 8.2, 5.5 Hz, 1H). ^13^C NMR (151 MHz, CDCl_3_) δ 165.1, 162.6 (d, *J* = 247.4 Hz), 142.95, 129.9 (d, *J* = 8.5 Hz), 129.7 (d, *J* = 3.3 Hz), 119.0, 118.5, 115.8 (d, *J* = 21.5 Hz), 36.9, and 24.9, 23.3. ^19^F NMR (565 MHz, CDCl_3_) δ −114.1 (m). HRMS (MALDI-rTOF) *m*/*z* [M + H]^+^ calc.: 245.0544, found: 245.0547.

*Trans*-2-(4-chlorophenyl)-1-(thiazol-2-yl)cyclopropane-1-carbonitrile (**5c**)

Following the general procedure and using cyclopropane **1c** (20.2 mg), product **5c** was obtained in a 51% yield (13.2 mg) after chromatographic purification on silica gel (40% diethyl ether in cyclohexane, R_f_: 0.45) as a yellow oil. The diastereomeric ratio was determined to be 15:1 by crude ^1^H-NMR analysis. NMR signals refer to the major *trans*-diastereoisomer. ^1^H NMR (400 MHz, CDCl_3_) δ 7.71 (d, *J* = 3.3 Hz, 1H), 7.39–7.35 (m, 2H), 7.28 (d, *J* = 3.3 Hz, 1H), 7.27–7.24 (m, 2H), 3.32 (t, *J* = 8.6 Hz, 1H), 2.45 (dd, *J* = 9.1, 5.6 Hz, 1H), and 2.29 (dd, *J* = 8.2, 5.6 Hz, 1H). ^13^C NMR (151 MHz, CDCl_3_) δ 165.1, 143.1, 134.4, 132.6, 129.6, 129.2, 119.2, 118.5, 37.0, 24.95, and 23.5. HRMS (MALDI-rTOF) *m*/*z* [M + H]^+^ calc.: 261.0248; 263.0219, found: 261.0254; 263.0221.

*Trans*-2-(4-bromophenyl)-1-(thiazol-2-yl)cyclopropane-1-carbonitrile (**5d**)

Following the general procedure and using cyclopropane **1d** (32.8 mg), product **5d** was obtained in a 44% yield (18.0 mg) after chromatographic purification on silica gel (40% diethyl ether in cyclohexane, R_f_: 0.42) as a yellow oil. The diastereomeric ratio was determined to be 10:1 by crude ^1^H-NMR analysis. NMR signals refer to the major *trans*-diastereoisomer. ^1^H NMR (600 MHz, DMSO) δ 7.82 (d, *J* = 3.3 Hz, 1H), 7.75 (d, *J* = 3.3 Hz, 1H), 7.61 (d, *J* = 8.5 Hz, 2H), 7.41 (d, *J* = 8.5 Hz, 2H), 3.35 (t, *J* = 8.7 Hz, 1H), 2.76 (dd, *J* = 8.7, 6.1 Hz, 1H), and 2.35 (dd, *J* = 8.7, 6.1 Hz, 1H). ^13^C NMR (151 MHz, DMSO) δ 165.2, 143.5, 134.25, 131.9, 131.0, 121.8, 121.0, 118.9, 37.1, 24.0, and 23.8. HRMS (MALDI-rTOF) *m*/*z* [M + H]^+^ calc.: 304.9743; 306.9723, found: 304.9746; 306.9725.

*Trans*-2-(4-iodophenyl)-1-(thiazol-2-yl)cyclopropane-1-carbonitrile (**5e**)

Following the general procedure and using cyclopropane **1e** (53 mg), product **5f** was obtained in a 21% yield (13.6 mg) after chromatographic purification on silica gel (40% diethyl ether in cyclohexane, R_f_: 0.44) as a yellow oil. The diastereomeric ratio was determined to be 10:1 by crude ^1^H-NMR analysis. NMR signals refer to the major *trans*-diastereoisomer. ^1^H NMR (400 MHz, CDCl_3_) δ 7.75–7.69 (m, 3H), 7.28 (d, *J* = 3.3 Hz, 1H), 7.09–7.04 (m, 2H), 3.28 (t, *J* = 8.6 Hz, 1H), 2.45 (dd, *J* = 9.1, 5.6 Hz, 1H), and 2.29 (dd, *J* = 8.2, 5.6 Hz, 1H). ^13^C NMR (151 MHz, CDCl_3_) δ 164.9, 143.0, 137.9, 133.1, 129.9, 119.1, 118.3, 94.0, 37.05, 24.7, and 23.3. HRMS (MALDI-rTOF) *m*/*z* [M + H]^+^ calc.: 352.9604, found: 352.9605.

*Trans*-2-(2-chlorophenyl)-1-(thiazol-2-yl)cyclopropane-1-carbonitrile (**5f**)

Following the general procedure and using cyclopropane **1f** (250 mg), product **5f** was obtained in a 67% yield (216 mg) after chromatographic purification on silica gel (40% diethyl ether in cyclohexane, R_f_: 0.43) as a yellow solid. The diastereomeric ratio was determined to be 12:1 by crude. IR (ATR, cm^−1^): 3100–3000 (CH, w), 2245 (CN, s), and 1593 (C=C, m). NMR signals refer to the major *trans*-diastereoisomer. ^1^H-NMR analysis. ^1^H NMR (400 MHz, CDCl_3_) δ 7.72 (d, *J* = 3.2 Hz, 1H), 7.48 (d, *J* = 3.5 Hz, 1H), 7.35–7.26 (m, 4H), 3.35 (t, *J* = 8.6 Hz, 1H), 2.55 (dd, *J* = 8.9, 5.5 Hz, 1H), and 2.33 (dd, *J* = 8.3, 5.5 Hz, 1H). ^13^C NMR (151 MHz, CDCl_3_) δ 165.1, 142.9, 136.75, 132.8, 129.88, 129.86, 129.1, 127.2, 119.3, 118.5, 36.3, 24.1, and 22.7. HRMS (MALDI-rTOF) *m*/*z* [M + H]^+^ calc.: 261.0248; 263.0219, found: 261.0255; 263.0234.

*Trans*-2-(3-chlorophenyl)-1-(thiazol-2-yl)cyclopropane-1-carbonitrile (**5g**)

Following the general procedure and using cyclopropane **1g** (10.0 mg), product **5g** was obtained in a 50% yield (6.4 mg) after chromatographic purification on silica gel (40% diethyl ether in cyclohexane, R_f_: 0.48) as a yellow oil. The diastereomeric ratio was determined to be 9:1 by crude ^1^H-NMR analysis. NMR signals refer to the major *trans*-diastereoisomer. ^1^H NMR (400 MHz, CDCl_3_) δ 7.71 (d, *J* = 3.3 Hz, 1H), 7.39–7.31 (m, 3H), 7.29 (d, *J* = 3.3 Hz, 1H), 7.26–7.16 (m, 1H), 3.32 (t, *J* = 8.6 Hz, 1H), 2.45 (dd, *J* = 9.1, 5.6 Hz, 1H), and 2.31 (dd, *J* = 8.2, 5.6 Hz, 1H). ^13^C NMR (151 MHz, CDCl_3_) δ 164.9, 142.95, 135.9, 134.7, 130.0, 128.6, 128.5, 126.0, 119.1, 118.15, 36.8, 24.6, and 23.3. HRMS (MALDI-rTOF) *m*/*z* [M + H]^+^ calc.: 261.0248; 263.0219, found: 261.0256; 263.0223.

*Trans*-2-(2-bromophenyl)-1-(thiazol-2-yl)cyclopropane-1-carbonitrile (**5h**)

Following the general procedure and using cyclopropane **1h** (24.7 mg), product **5h** was obtained in a 78% yield (23.8 mg) after chromatographic purification on silica gel (40% diethyl ether in cyclohexane, R_f_: 0.44) as a yellow oil. The diastereomeric ratio was determined to be >20:1 by crude ^1^H-NMR analysis. NMR signals refer to the major *trans*-diastereoisomer. ^1^H NMR (400 MHz, DMSO) δ 7.80 (d, *J* = 3.3 Hz, 1H), 7.74–7.71 (m, 2H), 7.50–7.42 (m, 2H), 7.35–7.31 (m, 1H), 3.26 (t, *J* = 8.1 Hz, 1H), 2.72 (dd, *J* = 8.2, 6.0 Hz, 1H), and 2.39 (dd, *J* = 8.9, 6.0 Hz, 1H). ^13^C NMR (151 MHz, DMSO) δ 164.7, 142.9, 134.4, 132.6, 130.3, 130.0, 128.0, 126.3, 120.5, 118.4, 38.1, 23.9, and 22.5. HRMS (MALDI-rTOF) *m*/*z* [M + H]^+^ calc.: 304.9743; 306.9723, found: 304.9748; 306.9730.

*Trans*-2-(4-cyanophenyl)-1-(thiazol-2-yl)cyclopropane-1-carbonitrile (**5i**)

Following the general procedure and using cyclopropane **1i** (148 mg), product **5i** was obtained in a 60% yield (115 mg) after chromatographic purification on silica gel (40% diethyl ether in cyclohexane, R_f_: 0.18) as a yellow oil. The diastereomeric ratio was determined to be 13:1 by crude ^1^H-NMR analysis. IR (ATR, cm^−1^): 3115–3000 (CH, w), 2918 (CH, br), 2235 (CN, m), 2226 (CN, s), and 1606 (C=C, s). NMR signals refer to the major *trans*-diastereoisomer. ^1^H NMR (600 MHz, CDCl_3_) δ 7.73 (d, *J* = 3.3 Hz, 1H), 7.72–7.69 (m, 2H), 7.46–7.43 (m, 2H), 7.32 (d, *J* = 3.3 Hz, 1H), 3.40 (t, *J* = 8.6 Hz, 1H), 2.52 (dd, *J* = 9.1, 5.8 Hz, 1H), and 2.36 (dd, *J* = 8.2, 5.8 Hz, 1H). ^13^C NMR (151 MHz, CDCl_3_) δ 164.4, 143.2, 139.4, 132.7, 129.0, 119.6, 118.5, 118.05, 112.3, 36.9, 25.0, and 23.8. HRMS (MALDI-rTOF) *m*/*z* [M + H]^+^ calc.: 252.0590, found: 252.0597.

*Trans*-2-(4-trifluoromethylphenyl)-1-(thiazol-2-yl)cyclopropane-1-carbonitrile (**5j**)

Following the general procedure and using cyclopropane **1j** (47.2 mg), product **5j** was obtained in a 29% yield (17.1 mg) after chromatographic purification on silica gel (40% diethyl ether in cyclohexane, R_f_: 0.41) as a yellow oil. The diastereomeric ratio was determined to be 18:1 by crude ^1^H-NMR analysis. NMR signals refer to the major *trans*-diastereoisomer. ^1^H NMR (400 MHz, CDCl_3_) δ 7.72 (d, *J* = 3.3 Hz, 1H), 7.66 (d, *J* = 8.1 Hz, 2H), 7.45 (d, *J* = 8.6 Hz, 1H), 7.30 (d, *J* = 3.3 Hz, 1H), 3.40 (t, *J* = 8.6 Hz, 1H), 2.50 (dd, *J* = 9.1, 5.7 Hz, 1H), and 2.36 (dd, *J* = 8.2, 5.7 Hz, 1H). ^13^C NMR (151 MHz, CDCl_3_) δ 163.7, 142.0, 136.9, 129.4 (q, *J* = 32.7 Hz), 127.5, 124.75 (q, *J* = 3.8 Hz), 122.0 (q, *J* = 270.3 Hz), 118.2, 117.1, 35.8, 23.8, and 22.4. ^19^F NMR (565 MHz, CDCl_3_) δ -63.7. HRMS (MALDI-rTOF) *m*/*z* [M + H]^+^ calc.: 295.0512, found: 295.0519.

*Trans*-2-(4-nitrophenyl)-1-(thiazol-2-yl)cyclopropane-1-carbonitrile (**5k**)

Following the general procedure and using cyclopropane **1k** (29.7 mg), product **5k** was obtained in a 52% yield (19.8 mg) after chromatographic purification on silica gel (40% diethyl ether in cyclohexane, R_f_: 0.24) as a yellow oil. The diastereomeric ratio was determined to be >20:1 by crude NMR signals refer to the major *trans*-diastereoisomer. ^1^H-NMR analysis. ^1^H NMR (400 MHz, CDCl_3_) δ 8.27 (d, *J* = 8.8 Hz, 2H), 7.73 (d, *J* = 3.3 Hz, 1H), 7.50 (d, *J* = 8.4 Hz, 2H), 7.32 (d, *J* = 3.3 Hz, 1H), 3.45 (t, *J* = 8.6 Hz, 1H), 2.55 (dd, *J* = 9.0, 5.8 Hz, 1H), and 2.39 (dd, *J* = 8.2, 5.8 Hz, 1H). ^13^C NMR (151 MHz, DMSO) δ 164.3, 147.1, 143.1, 142.1, 129.7, 123.5, 120.8, 118.2, 36.5, 23.9, and 23.8. HRMS (MALDI-rTOF) *m*/*z* [M + H]^+^ calc.: 272.0488, found: 272.0492.

*Trans*-2-(3-nitrophenyl)-1-(thiazol-2-yl)cyclopropane-1-carbonitrile (**5l**)

Following the general procedure and using cyclopropane **1l** (42.6 mg), product **5l** was obtained in an 85% yield (45.1 mg) after chromatographic purification on silica gel (40% diethyl ether in cyclohexane, R_f_: 0.27) as a yellow oil. The diastereomeric ratio was determined to be 8:1 by crude ^1^H-NMR analysis. NMR signals refer to the major *trans*-diastereoisomer. ^1^H NMR (400 MHz, CDCl_3_) δ 8.24–8.18 (m, 2H), 7.72 (d, *J* = 3.3 Hz, 1H), 7.69–7.56 (m, 2H), 7.31 (d, *J* = 3.2 Hz, 1H), 3.46 (t, *J* = 8.6 Hz, 1H), 2.53 (dd, *J* = 9.1, 5.8 Hz, 1H), and 2.41 (dd, *J* = 8.1, 5.8 Hz, 1H). ^13^C NMR (151 MHz, CDCl_3_) δ 164.2, 148.4, 143.0, 136.1, 134.0, 129.8, 123.4, 123.3, 119.4, 117.9, 36.3, 24.6, and 23.4. HRMS (MALDI-rTOF) *m*/*z* [M + H]^+^ calc.: 272.0489, found: 272.0496.

*Trans*-2-(4-methoxyphenyl)-1-(thiazol-2-yl)cyclopropane-1-carbonitrile (**5m**)

Following the general procedure and using cyclopropane **1m** (39.6 mg), product **5m** was obtained in a 30% yield (15.6 mg) after chromatographic purification on silica gel (40% diethyl ether in cyclohexane, R_f_: 0.38) as a yellow oil. The diastereomeric ratio was determined to be >20:1 by crude ^1^H-NMR analysis. NMR signals refer to the major *trans*-diastereoisomer. ^1^H NMR (600 MHz, DMSO) δ 7.74 (d, *J* = 3.3 Hz, 1H), 7.66 (d, *J* = 3.2 Hz, 1H), 7.31–7.25 (m, 2H), 6.93–6.87 (m, 2H), 3.71 (s, 3H), 3.20 (t, *J* = 8.7 Hz, 1H), 2.60 (dd, *J* = 8.3, 6.0 Hz, 1H), and 2.25 (dd, *J* = 9.2, 6.0 Hz, 1H). ^13^C NMR (151 MHz, DMSO) δ 165.55, 159.4, 143.4, 129.9, 126.4, 120.6, 119.2, 114.3, 55.5, 37.5, 24.1, and 23.65. HRMS (MALDI-rTOF) *m*/*z* [M + H]^+^ calc.: 257.0743, found: 257.0746.

*Trans*-2-(2-methoxyphenyl)-1-(thiazol-2-yl)cyclopropane-1-carbonitrile (**5n**)

Following the general procedure and using cyclopropane **1n** (39.6 mg), product **5n** was obtained in a 52% yield (26.7 mg) after chromatographic purification on silica gel (40% diethyl ether in cyclohexane, R_f_: 0.35) as a yellow oil. The diastereomeric ratio was determined to be 11:1 by crude ^1^H-NMR analysis. NMR signals refer to the major *trans*-diastereoisomer. ^1^H NMR (400 MHz, DMSO) δ 7.80 (d, *J* = 3.2 Hz, 1H), 7.74 (d, *J* = 3.3 Hz, 1H), 7.36 (dt, *J* = 7.8, 1.7 Hz, 1H), 7.27 (d, *J* = 7.6 Hz, 1H), 7.08 (dd, *J* = 8.0, 1.1 Hz, 1H), 6.99 (td, *J* = 7.7, 1.1 Hz, 1H), 3.80 (s, 3H), 3.13 (t, *J* = 8.7 Hz, 1H), 2.55 (dd, *J* = 8.3, 5.7 Hz, 1H), and 2.33 (dd, *J* = 8.9, 5.7 Hz, 1H). ^13^C NMR (101 MHz, DMSO) δ 165.3, 158.8, 142.8, 129.6, 128.2, 122.9, 120.45, 120.3, 118.9, 110.9, 55.7, 33.0, 23.0, and 21.9. HRMS (MALDI-rTOF) *m*/*z* [M + H]^+^ calc.: 257.0743, found: 257.0747.

*Trans*-2-(2-methylphenyl)-1-(thiazol-2-yl)cyclopropane-1-carbonitrile (**5o**)

Following the general procedure and using cyclopropane **1o** (42 mg), product **5o** was obtained in a 78% yield (43.4 mg) after chromatographic purification on silica gel (40% diethyl ether in cyclohexane, R_f_: 0.48) as a yellow oil. The diastereomeric ratio was determined to be 12:1 by crude NMR signals refer to the major *trans*-diastereoisomer. ^1^H-NMR analysis. ^1^H NMR (400 MHz, DMSO) δ 7.83 (d, *J* = 3.3 Hz, 1H), 7.76 (d, *J* = 3.3 Hz, 1H), 7.32–7.22 (m, 4H), 3.26 (t, *J* = 8.6 Hz, 1H), 2.67 (dd, *J* = 8.6, 5.8 Hz, 1H), 2.35 (dd, *J* = 8.6, 5.8 Hz, 1H), and 2.26 (s, 3H). ^13^C NMR (151 MHz, DMSO) δ 165.5, 143.55, 139.0, 133.8, 130.4, 128.7, 127.95, 126.5, 121.0, 119.1, 36.5, 23.9, 22.5, and 19.7. HRMS (MALDI-rTOF) *m*/*z* [M + H]^+^ calc.: 241.0794, found: 241.0796.

*Trans*-1-(thiazol-2-yl)-2-(thiophen-2-yl)cyclopropane-1-carbonitrile (**5p**)

Following the general procedure and using cyclopropane **1p** (34.8 mg), product **5p** was obtained in a 27% yield (12.7 mg) after chromatographic purification on silica gel (40% diethyl ether in cyclohexane, R_f_: 0.45) as a yellow oil. The diastereomeric ratio was determined to be 11:1 b crude ^1^H-NMR analysis. NMR signals refer to the major *trans*-diastereoisomer. ^1^H NMR (600 MHz, CDCl_3_) δ 7.70 (d, *J* = 3.3 Hz, 1H), 7.33–7.25 (m, 2H), 7.07–7.01 (m, 2H), 3.44 (t, *J* = 8.9 Hz, 1H), 2.53 (dd, *J* = 9.1, 5.5 Hz, 1H), and 2.31 (dd, *J* = 7.9, 5.5 Hz, 1H). ^13^C NMR (151 MHz, CDCl_3_) δ 164.7, 143.1, 137.5, 127.5, 126.6, 126.0, 119.2, 118.5, 32.9, 26.6, and 24.5. HRMS (MALDI-rTOF) *m*/*z* [M + H]^+^ calc.: 233.0202, found: 233.0205.

*Trans*-2-(naphth-2-yl)-1-(thiazol-2-yl)cyclopropane-1-carbonitrile (**5q**)

Following the general procedure and using cyclopropane **1q** (16.9 mg), product **5r** was obtained in a 26% yield (5.6 mg) after chromatographic purification on silica gel (40% diethyl ether in cyclohexane, R_f_: 0.42) as a yellow oil. The diastereomeric ratio was determined to be 8:1 by crude ^1^H-NMR analysis. NMR signals refer to the major *trans*-diastereoisomer. ^1^H NMR (400 MHz, CDCl_3_) δ 7.93–7.77 (m, 4H), 7.73 (d, *J* = 3.3 Hz, 1H), 7.55–7.40 (m, 3H), 7.30 (d, *J* = 3.3 Hz, 1H), 3.51 (t, *J* = 8.6 Hz, 1H), and 2.57–2.46 (m, 2H). ^13^C NMR (151 MHz, CDCl_3_) δ 165.6, 143.1, 133.4, 133.2, 131.5, 128.8, 128.1, 127.9, 127.3, 126.7, 126.5, 126.0, 119.1, 118.7, 38.1, 25.0, and 23.6. HRMS (MALDI-rTOF) *m*/*z* [M + H]^+^ calc.: 277.0794, found: 277.0800.

*Trans*-2-(naphth-1-yl)-1-(thiazol-2-yl)cyclopropane-1-carbonitrile (**5r**)

Following the general procedure and using cyclopropane **5r** (18.7 mg), product **5s** was obtained in a 52% yield (12.2 mg) after chromatographic purification on silica gel (40% diethyl ether in cyclohexane, R_f_: 0.45) as a yellow oil. The diastereomeric ratio was determined to be >20:1 by crude ^1^H-NMR analysis. NMR signals refer to the major *trans*-diastereoisomer. ^1^H NMR (600 MHz, CDCl_3_) δ 7.91–7.87 (m, 2H), 7.85 (d, *J* = 8.1 Hz, 1H), 7.77 (d, *J* = 3.3 Hz, 1H), 7.53–7.39 (m, 5H), 7.33 (d, *J* = 3.3 Hz, 1H), 3.76 (t, *J* = 8.5 Hz, 1H), and 2.56–2.48 (m, 2H). ^13^C NMR (151 MHz, CDCl_3_) δ 165.5, 143.3, 133.9, 133.1, 130.8, 129.5, 129.1, 127.1, 126.4, 125.5, 125.4, 123.4, 119.4, 118.6, 35.5, 24.7, and 23.0. HRMS (MALDI-rTOF) *m*/*z* [M + H]^+^ calc.: 277.0794, found: 277.0803.

### 3.5. Synthesis of Trans-2-phenyl-1-(thiazol-2-yl)cyclopropyl)methanamine (***7a***)

To a solution of compound **5a** (54.1 mg, 240 μmol) in dry THF (2.0 mL, 0.2 M) at room temperature, a solution of borane–THF complex (1.0 M in THF, 0.5 mL, 2.0 equiv.) was added dropwise. The reaction mixture was then heated at 60 °C overnight under an inert atmosphere. After completion (monitored by TLC), the mixture was cooled to 0 °C and carefully quenched with saturated aqueous NaHCO_3_. The resulting mixture was extracted with EtOAc (3×), and the combined organic layers were washed with brine, dried over anhydrous Na_2_SO_4_, filtered, and concentrated under reduced pressure. The crude product was purified by flash column chromatography on silica (40% diethyl ether in cyclohexane) to afford the amine **7a** in 10% (5.6 mg, 6:1 d.r., d.e. 85%).

The same reduction was also carried out using 2 equiv. of LiAlH_4_ (1M solution in THF) at room temperature obtaining **7a** in a 34% yield, with a 3:1 dr, indicating that epimerization under these conditions occurred more rapidly. ^1^H NMR (600 MHz, CDCl_3_) δ 7.65 (d, *J* = 3.4 Hz, 1H), 7.33–7.25 (m, 5H), 7.19 (d, *J* = 3.3 Hz, 1H), 2.99 (d, *J* = 14.0 Hz, 1H), 2.89 (t, *J* = 8.4 Hz, 1H), 2.79 (d, *J* = 14.0 Hz, 1H), 2.37 (bs, 2H), and 1.76–1.68 (m, 2H). ^13^C NMR (151 MHz, CDCl_3_) δ 175.1, 142.0, 135.9, 128.8, 128.55, 127.1, 117.4, 44.4, 34.7, 29.7, and 21.1. HRMS (MALDI-rTOF) *m*/*z* [M + H]^+^ calc.: 231.0951, found: 231.0955.

### 3.6. Reactivity of Thiazolyl Derivatives ***5***

#### 3.6.1. General Procedure for the Dichlorination of Compounds **5**

In a 4 mL vial, aryl-substituted 2-aryl-1-(thiazol-2-yl)cyclopropane-1 carbonitrile (100 μmol, 1 equiv.), phenyliodine dichloride (120 μmol, 1.2 equiv.), and DCM (1.33 mL, 75 mM) were added. The reaction mixture was stirred at room temperature until total conversion of the substrate was observed via TLC. The crude mixture was loaded on a silica-packed column and purified by flash column chromatography using mixtures of diethyl ether and cyclohexane as the eluent.

2,4-Dichloro-4-phenyl-2-(thiazol-2-yl)butanenitrile (**8a**)

Following the general procedure and using cyclopropane **5a** (22.6 mg), product **8a** was obtained in a 75% yield (22.2 mg) after chromatographic purification on silica gel (40% diethyl ether in cyclohexane, R_f_:0.57) as a yellow oil. The diastereomeric ratio was determined to be 1:1 by ^1^H-NMR analysis. ^1^H NMR (600 MHz, CDCl_3_) mixture of two diastereoisomers δ 7.84 (d, *J* = 3.2 Hz, 1H), 7.82 (d, *J* = 3.2 Hz, 1H), 7.49 (d, *J* = 3.2 Hz, 1H), 7.46 (d, *J* = 3.2 Hz, 1H), 7.42–7.30 (m, 10H), 5.25 (dd, *J* = 7.8, 5.7 Hz, 1H), 5.19 (t, *J* = 7.0 Hz, 1H), 3.52 (dd, *J* = 15.0, 7.8 Hz, 1H), 3.48 (dd, *J* = 14.9, 7.1 Hz, 1H), 3.43 (dd, *J* = 14.9, 6.7 Hz, 1H), and 3.31 (dd, *J* = 15.0, 5.7 Hz, 1H). ^13^C NMR (151 MHz, CDCl_3_) δ 165.0 (d1), 164.7 (d2), 143.83 (d1), 143.78 (d2), 139.5 (d1), 139.0 (d2), 129.3 (d1) 129.2 (d2), 128.96 (d1), 128.94 (d2), 127.4 (d1), 127.2 (d1), 122.6 (d1 + d2), 116.1 (d1), 115.8 (d2), 57.78 (d1), 57.76 (d2), 55.9 (d1), 55.7 (d2), and 51.65 (d1 + d2). HRMS (ESI-rTOF) *m*/*z* [M + H–NH_3_]^2+^ calc.: 139.9872, found: 139.9885.

2,4-Dichloro-4-(4-cyanophenyl)-2-(thiazol-2-yl)butanenitrile (**8b**)

Following the general procedure and using cyclopropane **5i** (25.1 mg), product **8b** was obtained in a 58% yield (18.8 mg) after chromatographic purification on silica gel (40% diethyl ether in cyclohexane) as a waxy pale-yellow solid. The diastereomeric ratio was determined to be 1:1.3 by ^1^H-NMR analysis. ^1^H NMR ^1^H NMR (600 MHz, CDCl_3_) mixture of major (M) and minor (m) diastereoisomers δ 7.84 (d, *J* = 3.2 Hz, 1H-M), 7.81 (d, *J* = 3.2 Hz, 1H-m), 7.68–7.64 (m, 2H-M + 2H-m), 7.54–7.51 (m, 3H-M + 2H-m), 7.50 (d, *J* = 3.2 Hz, 1H-m), 5.29 (dd, *J* = 7.4, 6.1 Hz, 1H-M), 5.23 (t, *J* = 6.9 Hz, 1H-m), 3.52–3.40 (dd, *J* = 15.1, 7.4 Hz, 1H-M and m, 2H-m), and 3.32 (dd, *J* = 15.1, 6.1 Hz, 1H-M). ^13^C NMR (151 MHz, CDCl_3_) δ 164.6 (M), 164.2 (m), 144.3 (m), 143.90 (M), 143.88 (M + m), 132.76 (M), 132.73 (m), 128.3 (m), 128.2 (M), 122.93 (m), 122.91(M), 118.11 (m), 118.11 (M), 115.9 (M), 115.7 (m), 113.1 (m), 113.0 (M), 56.5 (M + m), 55.5 (M), 55.1 (m), 51.14 (m), and 51.12 (M). HRMS (ESI-rTOF) *m*/*z* [M − H]^−^ calc.: 319.9821, found: 319.9823.

2,4-Dichloro-4-(4-nitrophenyl)-2-(thiazol-2-yl)butanenitrile (**8c**)

Following the general procedure and using cyclopropane **5k** (27.1 mg), product **8c** was obtained in a 52% yield (17.8 mg) after chromatographic purification on silica gel (40% diethyl ether in cyclohexane) as a dark yellow solid. The diastereomeric ratio was determined to be 1:1 by ^1^H-NMR analysis. The diastereomeric ratio was determined to be 1:1.4 by ^1^H-NMR analysis. ^1^H NMR (600 MHz, CDCl_3_) mixture of major (M) and minor (m) diastereoisomers δ 8.23 (d, *J* = 7.3 Hz, 2H-M), 8.21 (d, *J* = 7.3 Hz, 2H-m), 7.84 (d, *J* = 3.2 Hz, 1H-M), 7.82 (d, *J* = 3.2 Hz, 1H-m), 7.59 (m, 4H), 7.51 (d, *J* = 3.2 Hz, 1H-M), 7.50 (d, *J* = 3.2 Hz, 1H-m), 5.35 (dd, *J* = 7.3, 6.2 Hz, 1H-M), 5.28 (dd, *J*_1_ = *J*_2_= 6.9 Hz, 1H-m), 3.55–3.43 (several dd, 1H-M + 2H-m), and 3.35 (dd, *J* = 15.1, 6.2 Hz, 1H-M). ^13^C NMR (151 MHz, CDCl_3_) δ 163.5 (M), 163.1 (m), 147.12 (m), 147.06 (M), 145.15 (M), 144.7 (m), 142.9 (M + m), 127.6 (m), 127.5 (M), 123.17 (M), 123.14 (m), 121.96 (m), 121.93 (M), 114.8 (M), 114.7 (m), 55.1 (M + m), 54.5 (M), 54.0 (m), 52.4 (m), and 50.1 (M + m). HRMS (ESI-rTOF) *m*/*z* [M + H]^+^ calc.: 341.9865, found: 341.9854.

#### 3.6.2. General Procedure for the Hydrogenation of Compounds **5**

In a 10 mL glass tube, aryl-substituted 2-aryl-1-(thiazol-2-yl)cyclo propane-1-carbonitrile (50 μmol, 1 equiv.), palladium on aluminum oxide (10% loading, 26.6 mg, 0.5 equiv.), and methanol (500 μL, 100 mM) were added. The reaction vessel was first purged with nitrogen and then hydrogen. Next, a hydrogen balloon was connected to the reaction vessel via a rubber septum, and the reaction was heated to 70 °C for 24 h. The reaction mixture was purified via flash column chromatography using mixtures of diethyl ether and cyclohexane as the eluent.

4-Phenyl-2-(thiazol-2-yl)butanenitrile (**9a**)

Following the general procedure and using cyclopropane **5a** (14 mg), product **9a** was obtained in a 74% yield (10.4 mg) after chromatographic purification on silica gel (40% diethyl ether in cyclohexane) as a pale-yellow oil. ^1^H NMR (600 MHz, CDCl_3_) δ 7.76 (d, *J* = 3.3 Hz, 1H), 7.34 (d, *J* = 3.3 Hz, 1H), 7.30 (dd, *J* = 8.2, 6.9 Hz, 2H), 7.23–7.19 (m, 3H), 4.19 (t, *J* = 7.5 Hz, 1H), 2.92–2.82 (m, 2H), and 2.42 (q, *J* = 8.2 Hz, 2H). ^13^C NMR (151 MHz, CDCl_3_) δ 163.35, 143.2, 139.1, 128.8, 128.5, 126.7, 120.2, 118.4, 35.7, 34.75, and 32.7. HRMS (MALDI-rTOF) *m*/*z* [M + H]^+^ calc.: 229.0794, found: 229.0798.

4-(2-Chlorophenyl)-2-(thiazol-2-yl)butanenitrile (**9b**)

Following the general procedure and using cyclopropane **5f** (13.4 mg), product **9b** was obtained in a 92% yield (12.1 mg) after chromatographic purification on silica gel (40% diethyl ether in cyclohexane) as a yellow oil. ^1^H NMR (600 MHz, CDCl_3_) δ 7.79 (d, *J* = 3.3 Hz, 1H), 7.38 (d, *J* = 3.3 Hz, 1H), 7.36 (dd, *J* = 7.5, 1.7 Hz, 1H), 7.28–7.26 (m, 1H), 7.23–7.17 (m, 2H), 4.27 (dd, *J* = 8.7, 6.1 Hz, 1H), 3.08–2.93 (m, 2H), and 2.51–2.39 (m, 2H). ^13^C NMR (151 MHz, CDCl_3_) δ 163.6, 143.6, 137.3, 134.35, 131.1, 130.2, 128.65, 127.5, 120.7, 118.7, 35.4, 34.2, and 31.3. HRMS (ESI-rTOF) *m*/*z* [M + H]^+^ calc.: 263.0405, found: 263.0403.

2-(Thiazol-2-yl)-4-(4-(trifluoromethyl)phenyl)butanenitrile (**9c**)

Following the general procedure and using cyclopropane **5j** (52.6 mg), product **9c** was obtained in an 89% yield (47 mg) after chromatographic purification on silica gel (40% diethyl ether in cyclohexane, R_f_: 0.49) as a yellow oil. ^1^H NMR (600 MHz, CDCl_3_) δ 7.79 (d, *J* = 3.3 Hz, 1H), 7.60–7.56 (m, 2H), 7.39 (d, *J* = 3.3 Hz, 1H), 7.36–7.34 (m, 2H), 4.24 (dd, *J* = 7.8, 6.9 Hz, 1H), 3.03–2.88 (m, 2H), and 2.46 (tdd, *J* = 7.9, 6.7, 0.7 Hz, 2H). ^13^C NMR (151 MHz, CDCl_3_) δ 163.25, 143.6 (d, *J* = 1.8 Hz), 129.4 (q, *J* = 32.3 Hz), 129.2, 126.1 (q, *J* = 3.8 Hz), 124.5 (q, *J* = 271.9 Hz), 120.7, 118.6, 35.6, 35.1, and 32.9. ^19^F NMR (565 MHz, CDCl_3_) δ −63.4. HRMS (ESI-rTOF) *m*/*z* [M + H]^+^ calc.: 297.0668, found: 297.0679.

#### 3.6.3. General Procedure for the Arylation of Compounds **5**

In a 4 mL vial, aryl-substituted 2-aryl-1-(thiazol-2-yl)cyclo propane-1-carbonitrile (250 μmol, 1 equiv.), 1,3,5-trimethoxybenzene (2 equiv.), hexafluoroisopropanol (125 μL), and trifluoromethanesulfonic acid (0.1 equiv.) were added. The resulting mixture was stirred at room temperature until complete consumption of the starting material was observed by TLC, typically within 2–4 h. Upon completion, the reaction was quenched by the addition of water and extracted with DCM. The organic layers were dried over anhydrous sodium sulfate, filtered, and concentrated under reduced pressure. The crude residue was purified by flash column chromatography on silica gel using mixtures of diethyl ether and cyclohexane as the eluent.

4-Phenyl-2-(thiazol-2-yl)-4-(2,4,6-trimethoxyphenyl)butanenitrile (**10a**)

Following the general procedure and using cyclopropane **5a** (56.6 mg), product **10a** was obtained in a 52% yield (51.3 mg) after chromatographic purification on silica gel (40% diethyl ether in cyclohexane) as a pale-yellow solid. The diastereomeric ratio was deter-mined to be 1:1.5 by ^1^H NMR. ^1^H NMR (600 MHz, CDCl_3_) mixture of major (M) and minor (m) diastereoisomers δ 7.77 (d, *J* = 3.3 Hz, 1H-m), 7.74 (d, *J* = 3.3 Hz, 1H-M), 7.32–7.29 (m, 3H-m + 3H-M), 7.25–7.20 (m, 2H-m + 2H-M), 7.16–7.11 (m, 1H-m + 1H-M), 6.15 (s, H-M), 6.10 (s, 2H-m), 5.04 (dd, *J* = 11.8, 5.1 Hz, 1H-M), 4.73 (t *J* = 8.2 Hz, 1H-m), 4.09 (t, *J* = 7.5 Hz, 1H-m), 4.05 (dd, *J* = 11.3, 4.6 Hz, 1H-M), 3.80 (s, 3H-M), 3.79 (s, 3H-m), 3.76 (s, 6H-M), 3.71 (s, 6H-m), 3.26–3.18 (ddd, *J* = 4.5, 11,8, 13,0 Hz, 1H-m), 3.13–3.02 (m, 1H-m + 1H-M), and 2.74 (ddd, *J* = 13.0, 11.3, 5.1 Hz, 1H-M). ^13^C NMR (151 MHz, CDCl_3_) δ 164.6 (M), 163.7 (m) 160.4 (M), 160.3 (m), 159.5 (M), 159.2 (m), 143.6 (M), 143.1 (m), 143.03 (m), 142.96 (M), 128.05 (m), 127.96 (M), 127.8 (m), 127.7 (M), 126.0 (m), 125.85 (M), 119.99 (m), 119.92 (M), 119.1 (m), 118.7 (M), 110.3 (m), 109.6 (M), 91.2 (M), 91.0 (m), 55.7 (m), 55.5 (M), 55.3 (M), 55.2 (m), 37.6 (m), 37.5 (M), 37.2 (M), 36.85 (m), 34.7 (M), and 34.0 (m). HRMS (ESI-rTOF) *m*/*z* [M + H]^+^ calc.: 395.1424, found: 395.1416.

4-(2-Chlorophenyl)-2-(thiazol-2-yl)-4-(2,4,6-trimethoxyphenyl)butanenitrile (**10b**)

Following the general procedure and using cyclopropane **5f** (65.2 mg), product **10b** was obtained in an 84% yield (90.6 mg) after chromatographic purification on silica gel (40% diethyl ether in cyclohexane) as a pale-yellow. The diastereomeric ratio was determined to be 1:1.4 by ^1^H NMR. ^1^H NMR (600 MHz, CDCl_3_) mixture of major (M) and minor (m) diastereoisomers δ 7.68 (d, *J* = 3.3 Hz, 1H-m), 7.64 (d, *J* = 3.3 Hz, 1H-M), 7.36 (dd, *J* = 7.9, 1.7 Hz, 1H-m),7.30 (dd, *J* = 7.8, 1.7 Hz, 1H-M), 7.25 (d, *J* = 3.3, Hz, 1H-m), 7.23–7.16 (m, 1H-M + 1H-m), 7.21 (d, *J* = 3.3, Hz, 1H-M), 7.09–7.06 (m, 1H-M + 1H-m), 7.00–6.98 (m, 1H-M + 1H-m), 6.07 (s, 2H-M), 6.04 (s, 2H-m), 5.25 (dd, *J* = 12.1, 4.5 Hz, 1H-M), 4.90 (dd, *J* = 10.3, 6.0 Ha, 1H-m), 4.08 (J = 8.5, 6.0 Hz, 1H-m), 4.05–4.01 (m, 1H-M), 3.72 (s, 3H-M), 3.71 (s, 3H-m), 3.68 (s, 6H-M), 3.63 (s, 6H-m), 3.06–2.98 (m, 1H-M + 1Hm), 2.79 (ddd, *J* = 13.4, 8.5, 6.0 Hz, 1H-m), and 2.62 (ddd, *J* = 13.0, 11.5, 4.5 Hz, 1H-M). ^13^C NMR (151 MHz, CDCl_3_) δ 163.5 (M), 162.5 (m), 159.61 (M), 159.58 (m), 158.8 (M), 158.6 (m), 141.94 (m), 141.90 (M), 139.9 (M), 139.6 (m), 132.94 (m), 132.86 (M), 128.8 (m) 128.7 (M), 128.4 (M+m), 126.28 (m), 126.26 (M), 125.2 (m), 125.1 (M), 119.1 (m), 118.9 (M), 118.1 (m), 117.6 (M), 107.4 (m), 107.0 (M), 90.1 (M), 90.0 (m) 54.6 (M), 54.5 (m), 54.3 (M), 54.2 (m) 36.5 (M), 36.2 (m), 34.9 (M), 34.4 (m), 33.6 (M), and 32.7 (m). HRMS (ESI-rTOF) *m*/*z* [M + H]^+^ calc.: 429.1035, found: 429.1043.

4-(3-Cyano-3-(thiazol-2-yl)-1-(2,4,6-trimethoxyphenyl)propyl)benzonitrile (**10c**)

Following the general procedure and using cyclopropane **5i** (62.8 mg), product **10c** was obtained in an 86% yield (89.7 mg) after chromatographic purification on silica gel (40% diethyl ether in cyclohexane, R_f_: 0.27) as a pale-yellow solid. The diastereomeric ratio was determined to be 1:1.4 by ^1^H NMR. ^1^H NMR (600 MHz, CDCl_3_) mixture of major (M) and minor (m) diastereoisomers δ 7.80 (d, *J* = 3.3 Hz, 1H-m), 7.77 (d, *J* = 3.3 Hz, 1H-M), 7.53–7.50 (m, 2H-M+2H-m), 7.40–7.37 (m, 2H-M+2H-m), 7.36 (d, *J* = 3.3 Hz, 1H-m), 7.35 (d, *J* = 3.3 Hz, 1H-M), 6.15 (s, 2H-M), 6.10 (s, 2H-m), 5.08 (dd, *J* = 11.9, 4.7 Hz, 1H-M), 4.76 (dd, *J* = 9.6, 6.6 Hz, 1H-m), 4.12 (dd, *J* = 8.5, 5.8 Hz, 1H-m), 4.07 (dd, *J* = 11.5, 4.2 Hz, 1H-M), 3.81 (s, 3H-M), 3.80 (s, 3H-m), 3.76 (s, 6H-M), 3.70 (s, 6H-m), 3.19 (ddd, *J* = 12.2, 4.4 Hz, 1H-M), 3.14–3.02 (m, 2H-m), and 2.72 (ddd, *J* = 12.0, 4.8 Hz, 1H-M). ^13^C NMR (151 MHz, CDCl_3_) δ 164.37 (M), 163.5 (m), 160.93 (M), 160.86 (m), 159.3 (M), 159.1 (m), 149.34 (M), 148.95 (m), 143.13 (m), 143.09 (M), 131.85 (m), 131.8 (M), 128.5 (m), 128.4 (M), 120.2 (m), 120.1 (M), 119.20 (M), 119.17 (m), 118.7 (m), 118.4 (M), 109.6 (m), 109.5 (M), 108.7 (m), 108.3 (M), 91.2 (M), 91.0 (m), 55.7 (m), 55.5 (M), 55.35 (M), 55.32 (m), 37.4 (M), 37.1 (m), 36.6 (M), 35.9 (m), 34.5 (M), and 33.6 (m). HRMS (ESI-rTOF) *m*/*z* [M + H]^+^ calc.: 420.1377, found: 420.1383.

## 4. Conclusions

We have developed a novel and efficient reaction between D–A cyclopropane-1,1-dicarbonitriles **1** and 1,4-dithiane-2,5-diol **2** that enables the straightforward synthesis of hydroxythiazoline derivatives **4**. Under optimized phase-transfer catalysis (PTC) conditions, the reaction proceeds with high diastereoselectivity, affording an approximately 1:1 mixture of *trans*-diastereomers. Hydroxythiazoline intermediates **4** could be directly dehydrated using Burgess reagent in a one-pot sequence to yield thiazole derivatives **5** in good to excellent yields and with excellent retention of the diastereoselection achieved. The protocol proved scalable up to 5 mmol scale without significant loss in yield or selectivity.

The *trans*-configuration of the major thiazole diastereomer was established through NOE experiments on a reduced derivative **6**, and this stereochemical assignment was extended to the entire compound library. Additionally, the thiazolyl-substituted cyclopropanes **5** retained the typical reactivity of D–A cyclopropanes, undergoing classical ring-opening transformations, such as dichlorination, hydrogenolysis and arylation.

Overall, the synthetic sequence reported herein represents a practical and general method for the construction of functionalized thiazoles bearing a D–A cyclopropane moiety, offering new opportunities for the design of complex heterocyclic frameworks with potential relevance in medicinal and synthetic chemistry.

## Figures and Tables

**Table 1:** Reaction condition screening ^1^.

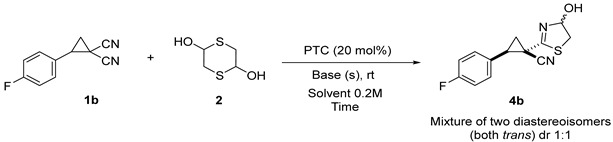					
Entry	Ammonium Salt	Base (equiv.)	Solvent	Reaction Time (min)	Yield 4b (%) ^2^
1	TBABr	K_2_CO_3_ (2.4 equiv.)	PhMe	60	20
2	BzTEABr	K_2_CO_3_ (2.4 equiv.)	PhMe	60	34
3	TMODAB	K_2_CO_3_ (2.4 equiv.)	PhMe	60	44
4	TMODAB	K_2_CO_3_ (1.2 equiv.)	PhMe	60	53
5	TMODAB	K_2_CO_3_ (1.2 equiv.)	EtOH	60	65
6	TMODAB	K_2_CO_3_ (1.2 equiv.)	*n*BuOH	60	62
7	TMODAB	K_2_CO_3_ (1.2 equiv.)	EtOAc	60	55
8	TMODAB	K_2_CO_3_ (1.2 equiv.)	CH_3_CN	60	34
9	TMODAB	K_2_CO_3_ (1.2 equiv.)	DCM	60	46
10	TMODAB	K_2_CO_3_ (1.2 equiv.)	1,2-DCE	60	52
11	TMODAB	K_2_CO_3_ (1.2 equiv.)	Et_2_O	60	42
12	TMODAB	K_2_CO_3_ (1.2 equiv.)	THF	60	40
13	TMODAB	K_2_CO_3_ (1.2 equiv.)	*t*BuOMe	60	48
14	TMODAB	K_2_CO_3_ (1.2 equiv.)	CPME	60	72
15	TMODAB	Cs_2_CO_3_ (1.2 equiv.)	CPME	60	75
16 ^3^	TMODAB	Cs_2_CO_3_ (1.2 equiv.)	CPME + 3 Å MS	60	78 (71)
17 ^3^	TMODAB	Cs_2_CO_3_ (1.2 equiv.)	CPME + 3 Å MS	240	68 (62)

^1^ Reaction conditions: **1b** (0.1 mmol); 1,4-dithiane-2,5-diol (0.1 mmol); ammonium salt (20 mol%); base (solid) in solvent (0.2M); and t. ^2^ Determined by ^19^F NMR using TFT as an internal standard, isolated yields are reported in parentheses (%). ^3^ Dry solvent was used. TBABr = tetrabutilammonium bromide; BzTEABr = benzyltrietilammonium bromide; TMODAB = tetrametiloctadecylammonium bromide.

**Table 2:** Dehydration protocols evaluation.

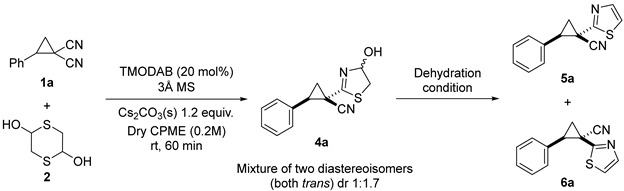			
Entry	Dehydration Condition ^1^	Yield (%) ^2^	5a:6a ^3^
1	TsCl/DIPEA	37	4:1
2	MsCl/Et_3_N	34	12:1
3	TsOH	34	3:1
4	TFA	17	3:1
5	Burgess reagent (2 equiv.)	50	20:1
6	Burgess reagent (3 equiv.)	69	13:1
7	Burgess reagent (4 equiv.)	78	14:1
8	Burgess reagent (6 equiv.)	38	16:1

^1^ Reaction conditions: **1a** (0.1 mmol); 1,4-dithiane-2,5-diol (0.1 mmol); ammonium salt (20 mol%); Cs_2_CO_3_ (solid); and 3 Å MS, in CPME (0.2M), upon complete conversion of **1a** dehydrating agent was added, and the resulting mixture was heated at 70 °C. ^2^ Isolated yields. ^3^ Determined by ^1^H-NMR.

## Data Availability

The original contributions presented in this study and all data (^1^H NMR, ^13^C NMR, HRMS, and IR) of all synthetized compounds are included within the article or reported in the Supplementary Materials. Further inquiries can be directed to the corresponding author.

## Supplementary Materials

The following supporting information can be downloaded at: https://www.mdpi.com/article/10.3390/molecules30183767/s1, NMR spectra of new compounds, NOE experiments, DFT calculations. References [30,76,77,78,79] are cited in the Supplementary Materials.

## Abbreviations

The following abbreviations are used in this manuscript:

D–A	Donor–acceptor
PTC	Phase transfer catalysis
PT	Phase transfer
TBABr	Tetrabutylammonium bromide
BzTEABr	Benzyltrietilammonium bromide
TMODAB	Tetrametiloctadecylammonium bromide
CPME	Cyclopentyl methyl ether
TFT	α,α,α-Trifluorotoluene
DIPEA	Diisopropylethylamine
TFA	Trifluoroacetic acid
HFIP	Hexafluoroisopropanol
