# Supplementary material for: Thiazolylcyanocyclopropanes: Novel Donor–Acceptor Cyclopropanes for Accessing Thiazole-Containing Targets
Source: Molecules. 2025 Sep 16;30(18):3767. doi: 10.3390/molecules30183767 (PMC12472231; doi:10.3390/molecules30183767)
Supplement: Supplementary file 1 [file molecules-30-03767-s001.zip › molecules-3821676-supplementary.pdf]

Supplementary material

Thiazolylcyanocyclopropanes: Novel Donor–Acceptor Cyclopropanes for Accessing Complex Thiazole-Containing Targets.

Emanuèl Bruno Savini, Edoardo Bandieri, Pietro Pecchini, Nicolò Santarelli, Luca Bernardi and Mariafrancesca Fochi.

Materials and methods: 3
Synthesis of 2-arylcyclopropane-1,1-dicarbonitriles (1) 4
Synthesis of 1-(4-hydroxy-4,5-dihydrothiazol-2-yl)-2-phenylcyclo propane-1-carbonitrile (4a) 11
General one-pot procedure for the synthesis of thiazoles (5) 12
Structural elucidation of compound 5a 13
Spectra of synthesized products 22
trans-2-phenyl-1-(thiazol-2-yl)cyclopropane-1-carbonitrile 5a 23
trans-(4-fluorophenyl)-1-(thiazol-2-yl)cyclopropane-1-carbonitrile 5b 25
trans-2-(4-chlorophenyl)-1-(thiazol-2-yl)cyclopropane-1-carbonitrile 5c 27
trans-2-(4-bromophenyl)-1-(thiazol-2-yl)cyclopropane-1-carbonitrile 5d 28
trans-2-(4-iodophenyl)-1-(thiazol-2-yl)cyclopropane-1-carbonitrile 5e 29
trans-2-(2-chlorophenyl)-1-(thiazol-2-yl)cyclopropane-1-carbonitrile 5f 30
trans-2-(3-chlorophenyl)-1-(thiazol-2-yl)cyclopropane-1-carbonitrile 5g 32
trans-2-(2-bromophenyl)-1-(thiazol-2-yl)cyclopropane-1-carbonitrile 5h 33
trans-2-(4-cyanophenyl)-1-(thiazol-2-yl)cyclopropane-1-carbonitrile 5i 34
trans-2-(4-trifluoromethylphenyl)-1-(thiazol-2-yl)cyclopropane-1-carbonitrile 5j 36
trans-2-(4-nitrophenyl)-1-(thiazol-2-yl)cyclopropane-1-carbonitrile 5k 38
trans-2-(3-nitrophenyl)-1-(thiazol-2-yl)cyclopropane-1-carbonitrile 5l 39
trans-2-(4-methoxyphenyl)-1-(thiazol-2-yl)cyclopropane-1-carbonitrile 5m 40
trans-2-(2-methoxyphenyl)-1-(thiazol-2-yl)cyclopropane-1-carbonitrile 5n 41
trans-2-(2-methylphenyl)-1-(thiazol-2-yl)cyclopropane-1-carbonitrile 5o 42
trans-1-(thiazol-2-yl)-2-(thiophen-2-yl)cyclopropane-1-carbonitrile 5p 43
trans-2-(naphth-2-yl)-1-(thiazol-2-yl)cyclopropane-1-carbonitrile 5r 44
trans-2-(naphth-1-yl)-1-(thiazol-2-yl)cyclopropane-1-carbonitrile 5s 45
trans-2-phenyl-1-(thiazol-2-yl)cyclopropyl)methanamine 7a 46

2,4-dichloro-4-phenyl-2-(thiazol-2-yl)butanenitrile 8a (mixture of diastereoisomers dr 1:1) 47

2,4-dichloro-4-(4-cyanophenyl)-2-(thiazol-2-yl)butanenitrile 8b (mixture of diastereo-isomers dr 1:1.3) 48

2,4-dichloro-4-(4-nitrophenyl)-2-(thiazol-2-yl)butanenitrile 8c (mixture of diastereo-isomers dr 1:1.4) 49

4-phenyl-2-(thiazol-2-yl)butanenitrile 9a 50

4-(2-chlorophenyl)-2-(thiazol-2-yl)butanenitrile 9b 51

2-(thiazol-2-yl)-4-(4-(trifluoromethyl)phenyl)butanenitrile 9c 52

4-phenyl-2-(thiazol-2-yl)-4-(2,4,6-trimethoxyphenyl)butanenitrile 10a (mixture of diastereo-isomers dr 1:1.5) 54

4-(2-chlorophenyl)-2-(thiazol-2-yl)-4-(2,4,6-trimethoxyphenyl)butanenitrile 10b (mixture of diastereoisomers dr 1:1.4) 55

4-(3-cyano-3-(thiazol-2-yl)-1-(2,4,6-trimethoxyphenyl)propyl)benzonitrile 10c (mixture of diastereoisomers dr 1:1.4) 56

Geometries: 57

Materials and methods:

NMR analyses were conducted using the following instruments: Bruker 600 MHz (all nuclei), Varian Inova 600 MHz (^{1}H, ^{13}C, NOE, 2D), Varian MR 400 MHz (^{1}H, ^{13}C, ^{19}F), Varian Mercury 300 MHz (^{1}H, ^{13}C, ^{19}F), Varian Mercury 400 MHz (^{1}H, ^{13}C, ^{19}F), Bruker Ascend Advance Neo 600 MHz, equipped with a Prodigy cryoprobe (All nuclei, 2D).
Chemical shifts (δ) are reported in ppm relative to residual $CHCl_3$ signals for 1H (7.26 ppm) and $CDCl_3$ for 13C (77.0 ppm) NMR, using $CF_3C_6H_5$ as reference calibrated at -63.72 ppm for ^{19}F NMR.
Chromatographic separations were carried out using a Büchi Chromatography system Pure C-815 Flash (FlashPure EcoFlex cartridge (4 to 40 g), 50 μm irregular) and by flash column chromatography using Silica 60 M (0,04-0,063mm) Macherey-Nagel.
Thin Layer Chromatography analysis was performed using Alumgram Xtra SIL G UV254 plates from Macherey-Nagel.
All HPLC-MS analyses were performed using an Agilent Infinity II 1260 system equipped with a DAD and ESI/SQ detector. Unless otherwise specified, separations were carried out using an Infinity Lab Poroshell 120 EC-C18, 4.6 x 150 mm, 2.7 μm with a gradient elution from 5% to 95% acetonitrile in water; the eluent contained 0.1% formic acid to enhance ionization.
GC-MS analyses were performed on an Agilent 8890 GC system coupled to a 5977C mass selective detector (MSD). Helium was used as the carrier gas at a constant flow rate of 1.2 mL/min. The oven temperature was programmed from 60 °C to 250 °C. The ionization source operated in EI mode at 70 eV and 230 °C, while the quadrupole was maintained at 150 °C, scanning in the m/z range of 50–550.
High-resolution mass spectrometry (HRMS) analyses were performed using two ionization techniques. Electrospray ionization (ESI) HRMS spectra were acquired on a Waters Xevo G2-XS QTof spectrometer operated in reflectron mode, using acetonitrile containing 0.1% formic acid as the mobile phase. Samples were introduced via the Aquicity H Plus UPLC autosampler in direct infusion mode. Matrix-assisted laser desorption/ionization (MALDI) HRMS spectra were obtained on a Waters Synapt MALDI Q-TOF G2S spectrometer, also operated in reflectron mode, employing α-cyano-4-hydroxycinnamic acid (4-HCCA, Sigma-Aldrich) as the ionization matrix.
FTIR Spectra were acquired on a Bruker Alpha II, operating in ATR mode.
NMR yields were determined using $\alpha\alpha\alpha$-trifluorotoluene, trimethoxybenzene, dinitrobenzene, dibromomethane, or ethylene carbonate as internal quantification standards.
Analytical grade solvents and commercially available reagents were used as received, unless otherwise stated. Dry THF was obtained by distillation from Na/benzophenone before use or through SPS system Pure Process Technology model PPT-SPS-5-CM. Dry CPME and DCM were prepared by storing them over microwave-activated 3Å molecular sieves. Solvent dryness was measured with a Karl-Fischer system, model Metrohm Eco Coulometer. The chemicals used for the reactions are commercial and used without further purification, provided by BLD pharm, Fluorochem, TCI, Thermo-Fischer, Sigma-Aldrich, unless otherwise stated.

Synthesis of 2-arylcyclopropane-1,1-dicarbonitriles (1)

D–A Cyclopropanes **1a-1s** (Scheme S1) were obtained from the corresponding styrene derivatives and malononitrile following a literature procedure using bisacetoxyiodobenzene (BAIB) and K_2CO_3 following literature procedure [75]. The corresponding styrene derivatives, if not commercially available, were obtained by Wittig reaction from the corresponding aldehydes

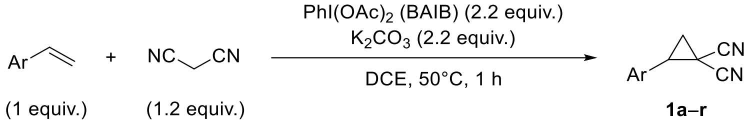

**1a**	Ar = C_6H_5	**1h**	Ar = 2-Br-C_6H_4	**1n**	Ar = 2-CH_3O-C_6H_4
**1b**	Ar = 4-F-C_6H_4	**1i**	Ar = 4-CN-C_6H_4	**1o**	Ar = 4-CH_3-C_6H_4
**1c**	Ar = 4-Cl-C_6H_4	**1j**	Ar = 4-CF_3-C_6H_4	**1p**	Ar = thiophen-2-yl
**1d**	Ar = 4-Br-C_6H_4	**1k**	Ar = 4-NO_2-C_6H_4	**1q**	Ar = naphthalen-2yl
**1e**	Ar = 4-I-C_6H_4	**1l**	Ar = 3-NO_2-C_6H_4	**1r**	Ar = naphthalen-1-yl
**1f**	Ar = 2-Cl-C_6H_4	**1m**	Ar = 4-CH_3O-C_6H		
**1g**	Ar = 3-Cl-C_6H_4				

Scheme S1. Synthetic procedure to D–A Cyclopropanes **1**.

In a 1-neck round-bottomed flask, in the following order, 1,2-dichloroethane (4.0 mL/mmol of styrene derivative), potassium carbonate (2.2 equiv.), substituted styrene derivative (1 equiv.), malononitrile (1.2 equiv.), and (diacetoxyiodo)benzene (2.2 equiv.) were mixed. The flask was fitted with an air-cooled condenser, and the reaction mixture was heated to 50 °C. After 1 hour, the reaction was quenched by adding water (5 mL/mmol of styrene derivative). The reaction mixture was vacuum filtered through paper, and the filter cake was washed with DCM. The filtrate was then extracted with DCM, and the combined organic layers were dried over anhydrous sodium sulfate. Celite was added to the dried organic phase to form a slurry, and the solvent was removed in vacuo. The desired product was purified by flash column chromatography on silica, usually employing 25% ethyl acetate in petroleum ether.

2-phenylcyclopropane-1,1-dicarbonitrile (1a)

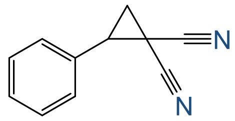

Prepared according to the standard procedure with a yield of 82%.
^{1}H NMR (400 MHz, $CDCl_3$) δ 7.49 – 7.37 (m, 3H), 7.36 – 7.25 (m, 2H), 3.34 – 3.25 (m, 1H), 2.25 (dq, *J* = 9.2, 6.4 Hz, 2H). NMR data are consistent with those previously reported in the literature [75, see also 76-78].

2-(4-fluorophenyl)cyclopropane-1,1-dicarbonitrile (1b)

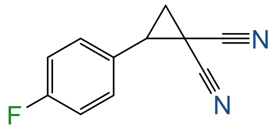

Prepared according to the standard procedure with a yield of 72%.
^{1}H NMR (400 MHz, $CDCl_3$) δ 7.29 (dddd, J = 8.8, 5.3, 2.6, 0.7 Hz, 2H), 7.16 – 7.09 (m, 2H), 3.31 – 3.25 (m, 1H), 2.28 – 2.20 (m, 2H). NMR data are consistent with those previously reported in the literature [76, see also 77,78].

2-(4-chlorophenyl)cyclopropane-1,1-dicarbonitrile (1c)

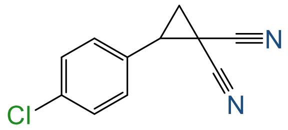

Prepared according to the standard procedure with a yield of 47%.
^{1}H NMR (400 MHz, $CDCl_3$) δ 7.40 – 7.38 (m, 2H), 7.29 – 7.26 (m, 2H), 3.26 (dd, J = 9.4, 8.7 Hz, 1H), 2.24 – 2.16 (m, 2H). NMR data are consistent with those previously reported in the literature [77,78].

2-(4-bromophenyl)cyclopropane-1,1-dicarbonitrile (1d)

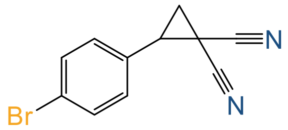

Prepared according to the standard procedure with a yield of 63%.
^{1}H NMR (400 MHz, $CDCl_3$) δ 7.60 – 7.53 (m, 2H), 7.21 – 7.14 (m, 2H), 3.25 (t, *J* = 9.0 Hz, 1H), 2.30 – 2.20 (m, 2H). NMR data are consistent with those previously reported in the literature [75, see also 77,78].

2-(4-iodophenyl)cyclopropane-1,1-dicarbonitrile (1e)

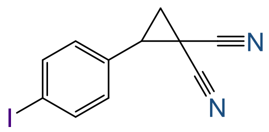

Prepared according to the standard procedure with a yield of 43%.
^{1}H NMR (600 MHz, $CDCl_3$) δ 7.79 – 7.74 (m, 2H), 7.07 – 7.01 (m, 2H), 3.24 (t, *J* = 9.0 Hz, 1H), 2.29 – 2.19 (m, 2H). ^{13}C NMR (151 MHz, $CDCl_3$) δ 138.43, 130.16, 115.10, 112.90, 95.72, 34.68, 22.37, 7.35. HRMS (ESI-rTOF) m/z $[M-H]^-$ calc: 292.9581, found: 292.9583.

2-(2-chlorophenyl)cyclopropane-1,1-dicarbonitrile (1f)

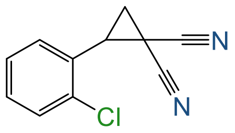

Prepared according to the standard procedure with a yield of 83%.
^{1}H NMR (600 MHz, $CDCl_3$) δ 7.54 (dd, *J* = 8.0, 1.2 Hz, 1H), 7.39 (tdd, *J* = 8.0, 1.6, 0.6 Hz, 1H), 7.32 (td, *J* = 7.6, 1.3 Hz, 1H), 7.19 – 7.14 (m, 1H), 3.35 (t, *J* = 9.0 Hz, 1H), 2.32 (dd, *J* = 9.2, 6.4 Hz, 1H), 2.27 (dd, *J* = 8.8, 6.4 Hz, 1H). ^{13}C NMR (151 MHz, $CDCl_3$) δ 136.68, 131.02, 130.22, 129.63, 129.16, 127.45, 115.10, 112.92, 33.63, 22.67, 7.08. HRMS (MALDI-rTOF) m/z $[M-H]^-$ calc: 201.0225; 203.0195, found: 201.0222; 203.0189.

2-(3-chlorophenyl)cyclopropane-1,1-dicarbonitrile (1g)

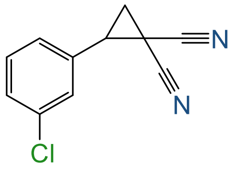

Prepared according to the standard procedure with a yield of 54%.
^{1}H NMR (400 MHz, $CDCl_3$) δ 7.40 – 7.34 (m, 2H), 7.31 (td, *J* = 1.8, 0.9 Hz, 1H), 7.19 (dtd, *J* = 6.6, 1.8, 0.6 Hz, 1H), 3.27 (t, *J* = 9.0 Hz, 1H), 2.31 – 2.18 (m, 2H). HRMS (ESI-rTOF) m/z $[M-H]^-$ calc: 201.0225, found: 201.0221.

2-(2-bromophenyl)cyclopropane-1,1-dicarbonitrile (1h)

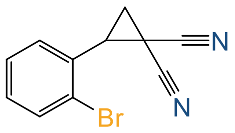

Prepared according to the standard procedure with a yield of 41%.
^{1}H NMR (400 MHz, $CDCl_3$) δ 7.72 (dd, *J* = 7.7, 1.4 Hz, 1H), 7.34 (dtd, *J* = 23.9, 7.5, 1.6 Hz, 2H), 7.15 (dd, *J* = 7.4, 1.8 Hz, 1H), 3.32 (t, *J* = 9.0 Hz, 1H), 2.35 – 2.25 (m, 2H). NMR data are consistent with those previously reported in the literature [76, see also 77,78].

2-(4-cyanophenyl)cyclopropane-1,1-dicarbonitrile (1i)

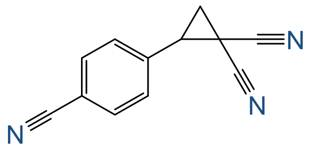

Prepared according to the standard procedure with a yield of 46%.
^{1}H NMR (600 MHz, $CDCl_3$) δ 7.76 – 7.70 (m, 2H), 7.46 – 7.40 (m, 2H), 3.34 (t, *J* = 9.0 Hz, 1H), 2.35 (dd, *J* = 9.4, 6.6 Hz, 1H), 2.30 (dd, *J* = 8.7, 6.7 Hz, 1H). ^{13}C NMR (151 MHz, $CDCl_3$) δ 135.88, 132.97, 129.31, 117.99, 114.65, 113.64, 112.55, 34.30, 22.46, 7.72. HRMS (MALDI-rTOF) m/z $[M+H]^+$ calc: 194.0713, found: 194.0715. NMR data are consistent with those previously reported in the literature [76].

2-(4-trifluoromethylphenyl)cyclopropane-1,1-dicarbonitrile (1j)

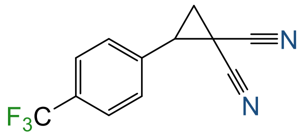

Prepared according to the standard procedure with a yield of 71%.
^{1}H NMR (400 MHz, $CDCl_3$) δ 7.71 (d, *J* = 8.0 Hz, 2H), 7.44 (d, *J* = 8.0 Hz, 2H), 3.34 (t, *J* = 9.0 Hz, 1H), 2.37 – 2.25 (m, 2H). NMR data are consistent with those previously reported in the literature [76, see also 77,78].

2-(4-nitrophenyl)cyclopropane-1,1-dicarbonitrile (1k)

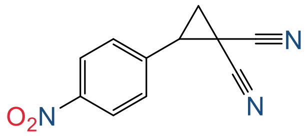

Prepared according to the standard procedure with a yield of 29%.
^{1}H NMR (400 MHz, $CDCl_3$) δ 8.33 – 8.29 (m, 2H), 7.53 – 7.48 (m, 2H), 3.41 – 3.34 (m, 1H), 2.40 – 2.31 (m, 2H). NMR data are consistent with those previously reported in the literature [76].

2-(3-nitrophenyl)cyclopropane-1,1-dicarbonitrile (1l)

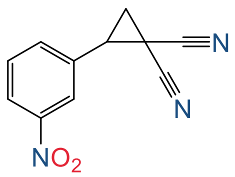

Prepared according to the standard procedure with a yield of 56%.
^{1}H NMR (600 MHz, $CDCl_3$) δ 8.30 (dt, *J* = 7.6, 1.9 Hz, 1H), 8.19 (q, *J* = 1.4 Hz, 1H), 7.71 – 7.63 (m, 2H), 3.41 (t, *J* = 9.0 Hz, 1H), 2.41 – 2.34 (m, 2H). ^{13}C NMR (151 MHz, $CDCl_3$) δ 148.66, 134.58, 132.97, 130.55, 124.66, 123.65, 114.61, 112.56, 33.99, 22.46, 7.69. HRMS (MALDI-rTOF) m/z $[M-H]^-$ calc: 212.0465, found: 212.0462. NMR data are consistent with those previously reported in the literature [61].

2-(4-methoxyphenyl)cyclopropane-1,1-dicarbonitrile (1m)

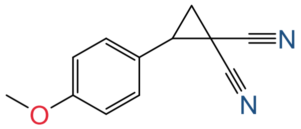

Prepared according to the standard procedure with a yield of 76%.
^{1}H NMR (400 MHz, $CDCl_3$) δ 7.24 – 7.20 (m, 2H), 6.95 – 6.91 (m, 2H), 3.81 (s, 3H), 3.26 (t, *J* = 9.0 Hz, 1H), 2.21 (d, *J* = 9.1 Hz, 2H). NMR data are consistent with those previously reported in the literature [76, see also 77,78].

2-(2-methoxyphenyl)cyclopropane-1,1-dicarbonitrile (1n)

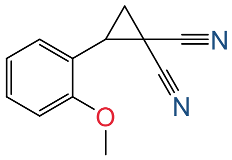

Prepared according to the standard procedure with a yield of 41%
^{1}H NMR (600 MHz, $CDCl_3$) δ 7.42 – 7.36 (m, 1H), 7.07 – 7.03 (m, 1H), 7.00 – 6.94 (m, 2H), 3.96 (s, 3H), 3.28 (t, *J* = 9.0 Hz, 1H), 2.24 – 2.15 (m, 2H). ^{13}C NMR (151 MHz, $CDCl_3$) δ 158.95, 130.92, 128.28, 120.57, 119.85, 115.76, 113.51, 110.70, 55.74, 31.15, 22.33. HRMS (MALDI-rTOF) m/z $[M+H]^+$ calc: 199.0866, found: 199.0873.

2-(2-methylphenyl)cyclopropane-1,1-dicarbonitrile (1o)

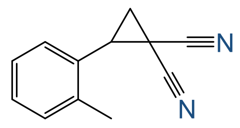

Prepared according to the standard procedure with a yield of 57%.
^{1}H NMR (400 MHz, $CDCl_3$) δ 7.35 – 7.29 (m, 2H), 7.25 – 7.20 (m, 1H), 7.07 – 7.04 (m, 1H), 3.20 (t, *J* = 9.0 Hz, 1H), 2.49 (s, 3H), 2.31 – 2.22 (m, 2H). NMR data are consistent with those previously reported in the literature [79].

2-(thiophen-2-yl)cyclopropane-1,1-dicarbonitrile (1p)

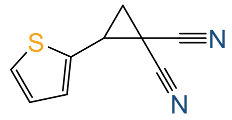

Prepared according to the standard procedure with a yield of 33%.
^{1}H NMR (600 MHz, $CDCl_3$) δ 7.37 (dd, *J* = 5.0, 1.3 Hz, 1H), 7.09 – 7.03 (m, 2H), 3.43 (ddd, *J* = 9.4, 8.4, 0.9 Hz, 1H), 2.32 (dd, *J* = 9.3, 6.4 Hz, 1H), 2.24 (dd, *J* = 8.4, 6.4 Hz, 1H). ^{13}C NMR (151 MHz, $CDCl_3$) δ 133.6, 128.2, 127.7, 127.5, 114.9, 112.9, 30.5, 24.1, 8.5. NMR data are consistent with those previously reported in the literature [75, see also 76].

2-(naphthalen-2-yl)cyclopropane-1,1-dicarbonitrile (1q)

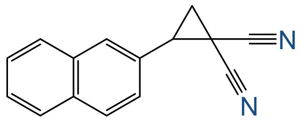

Prepared according to the standard procedure with a yield of 31%
^{1}H NMR (400 MHz, $cdcl_3$) δ 8.10 (dq, *J* = 8.4, 1.0 Hz, 1H), 8.00 – 7.88 (m, 2H), 7.71 (ddt, *J* = 8.3, 6.9, 1.2 Hz, 1H), 7.47 (dd, *J* = 8.3, 7.1 Hz, 1H), 7.33 (dt, *J* = 7.2, 1.2 Hz, 1H), 3.68 (t, *J* = 9.1 Hz, 1H), 2.49 – 2.37 (m, 2H). NMR data are consistent with those previously reported in the literature [77].

2-(naphthalen-1-yl)cyclopropane-1,1-dicarbonitrile (1r)

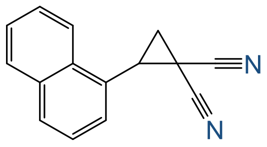

Prepared according to the standard procedure with a yield of 52%

^{1}H NMR (400 MHz, $CDCl_3$) δ 8.11 (dq, *J* = 8.4, 1.0 Hz, 1H), 8.01 – 7.88 (m, 2H), 7.72 (ddt, *J* = 8.3, 6.9, 1.2 Hz, 1H), 7.47 (dd, *J* = 8.3, 7.1 Hz, 1H), 7.33 (dt, *J* = 7.2, 1.2 Hz, 1H), 3.69 (t, *J* = 9.1 Hz, 1H), 2.50 – 2.37 (m, 2H). NMR data are consistent with those previously reported in the literature [77].

Synthesis of 1-(4-hydroxy-4,5-dihydrothiazol-2-yl)-2-phenylcyclo propane-1-carbonitrile (4a)

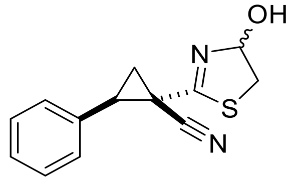

To an appropriately sized vial were added 2-phenylcyclopropane-1,1-dicarbonitrile **1a** (1 equiv, 0.1 mmol, 16.8 mg), 1,4-dithiane-2,5-diol **2** (1 equiv, 0.1 mmol, 15.2 mg), caesium carbonate (1.2 equiv, 1.2 mmol, 39 mg), trimethyl(octadecyl)ammonium bromide (0.2 equiv, 0.2 mmol, 7.8 mg), and microwave-activated 3Å molecular sieves (500 mg/mmol of **1a,** 50mg). The reagents were suspended in anhydrous cyclopentyl methyl ether (5 mL/mmol of **1**, 0.50 mL), and the reaction mixture was stirred vigorously at room temperature (1500 rpm) using a magnetic stirrer. The progress of the reaction was monitored by TLC (25% EtOAc in petroleum ether) until complete consumption of the starting material was observed. Reaction crude was evaporated under reduced pressure and purified by column chromatography (100% Et_2O), product **4a** was obtained in 76% yield (18.6 mg) as a 1:1.7 mixture of diastereoisomers at the -OH carbon of the *trans*-diastereoisomer since the *trans*:*cis* diastereomeric ratio was determined to be >20:1 by crude ^{1}H-NMR analysis.
^{1}H NMR (600 MHz, $CDCl_3$) Major *trans*-diasteroisomer δ 7.38 – 7.26 (m, 5H), 6.11 (m, 1H), 3.61 (dd, J = 12.1, 7.0 Hz, 1H), 3.38 (dd, J = 12.1, 4.6 Hz, 1H), 3.26 – 3.21 (m, 1H), 2.95 (broad d, J = 4.7 Hz, 1H), 2.31 – 2.26 (m, 1H), 2.23 – 2.19 (m, 1H).

The ^{1}H NMR signals of the minor *trans*-diastereoisomer largely overlap with those of the major *trans*-diastereoisomer; however, the two OH signals can be clearly distinguished at different chemical shifts: 2.95 ppm (broad d, J = 4.7 Hz, 1H) for the major and 2.98 ppm (broad d, J = 4.5 Hz, 1H) for the minor isomer in a ratio 1:1.7.

^{13}C NMR (151 MHz, $CDCl_3$) δ Major (M) and minor (m): 170.81 (m), 170.79 (M),133.28 (m), 133.22 (M), 128.8 (M+m), 128.5 (M+m), 128.2 (M+m), 117.48 (m), 117.46 (M), 98.8 (M+m), 40.94 (M), 40.91 (m), 37.22 (M), 36.97 (m), 24.49 (M), 24.10 (m), 23.9 (M+m).

HRMS (MALDI-rTOF) m/z $[M+Na]^+$ calc: 267.0563 found: 267.0568.

General one-pot procedure for the synthesis of thiazoles (5)

To an appropriately sized vial were added 2-arylcyclopropane-1,1-dicarbonitrile **1** (1.0 equiv), 1,4-dithiane-2,5-diol **2** (1 equiv), caesium carbonate (1 equiv), trimethyl(octadecyl)ammonium bromide (0.2 equiv), and microwave-activated 3Å molecular sieves (500 mg/mmol of **1**). The reagents were suspended in anhydrous cyclopentyl methyl ether (5 mL/mmol of **1**), and the reaction mixture was stirred vigorously at room temperature (1500 rpm) using a magnetic stirrer. The progress of the reaction was monitored by TLC (25% EtOAc in petroleum ether) until complete consumption of the starting material was observed. At this point, anhydrous tetrahydrofuran (5 mL/mmol of **1**) and Burgess reagent (4.0 equiv) were added (for reaction in smaller scale then 0.2mmol dilution of the reaction mixture with THF is not necessary). The reaction mixture was then heated to 75 °C under continuous stirring for 30 minutes to promote dehydration. Complete conversion of the hydroxythiazoline **4** was confirmed by TLC (100% diethyl ether as eluent on SiO_2). The crude reaction mixture was diluted with dichloromethane and adsorbed onto silica gel. After removal of solvents under reduced pressure, a free-flowing powder was obtained. Purification by flash column chromatography (40% diethyl ether in cyclohexane) yielded thiazoles **5** as a mixture of diastereoisomers, with a diastereomeric ratio unchanged from the one observed prior to chromatography.

Structural elucidation of compound 5a

The hydroxy thiazoline **4a** has been obtained as an approximately 1:1 mixture of two diastereoisomers both *trans* at the cyclopropane moiety, with different configuration at the alcoholic carbon with a diastereomeric ratio (*trans*/*cis*) of >20:1

The diastereomeric ratio between **5a** and **6a** was largely preserved during the dehydration step employing the Burgess reagent, maintaining an excellent ratio of 14:1 in favor of **5a** (corresponding to a 94% of **5a**, starting from >95% diastereomeric excess of **4a**) (Scheme S2).

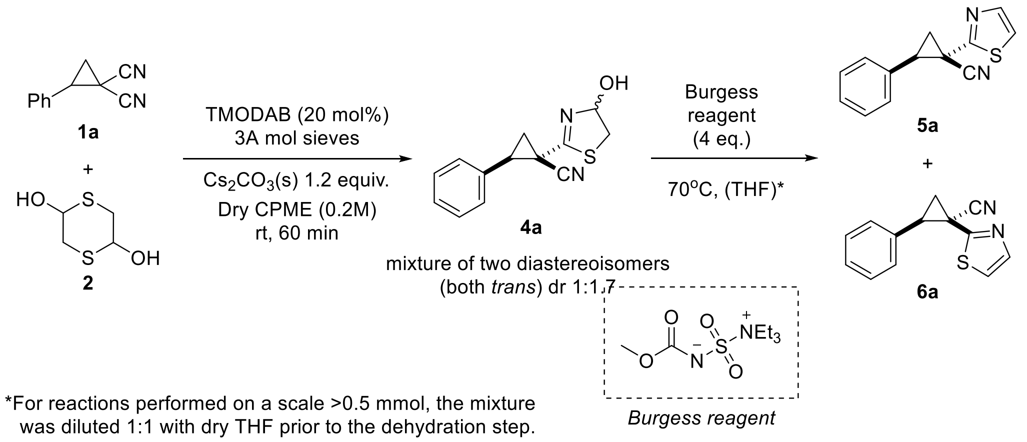

Scheme S2. Synthetic one pot procedure leading to **4a** and **5a**

1D and 2D NMR

1D NMR spectra of compound **5a** are presented in Figure S1-S2, 2D NMR spectra (gCOSY, gHMBC and gHSQC) are presented in Figure S3, S4 and S5.

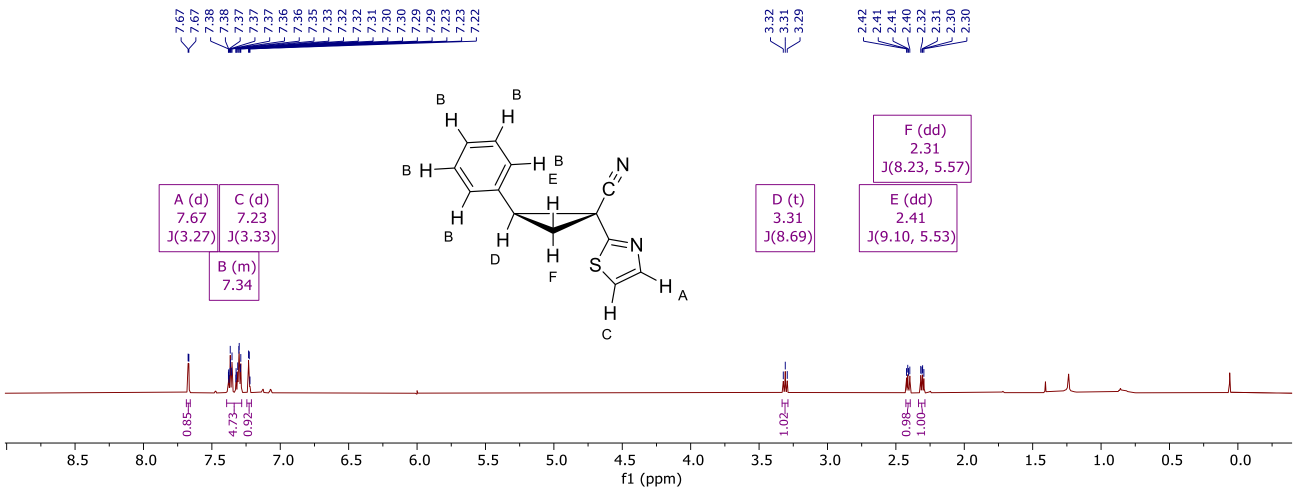

Figure S1. ^{1}H NMR of **5a**.

The obtained ^{1}H NMR spectrum of product **5a** is consistent with the proposed structure, although at first glance it could resemble that of a potential byproduct resulting from the

dehydration of intermediate **3a** (see main text, Scheme 4, pathway a). However, the exclusive formation of the cyano cycloaddition product is supported by a distinctive ³J coupling of 3.3 Hz between protons **A** and **C**, a value characteristic of heteroaromatic five-membered rings. In contrast, six-membered unsaturated rings derived from thiopyrans—such as the hypothetical alternative product—would be expected to exhibit significantly larger coupling constants.

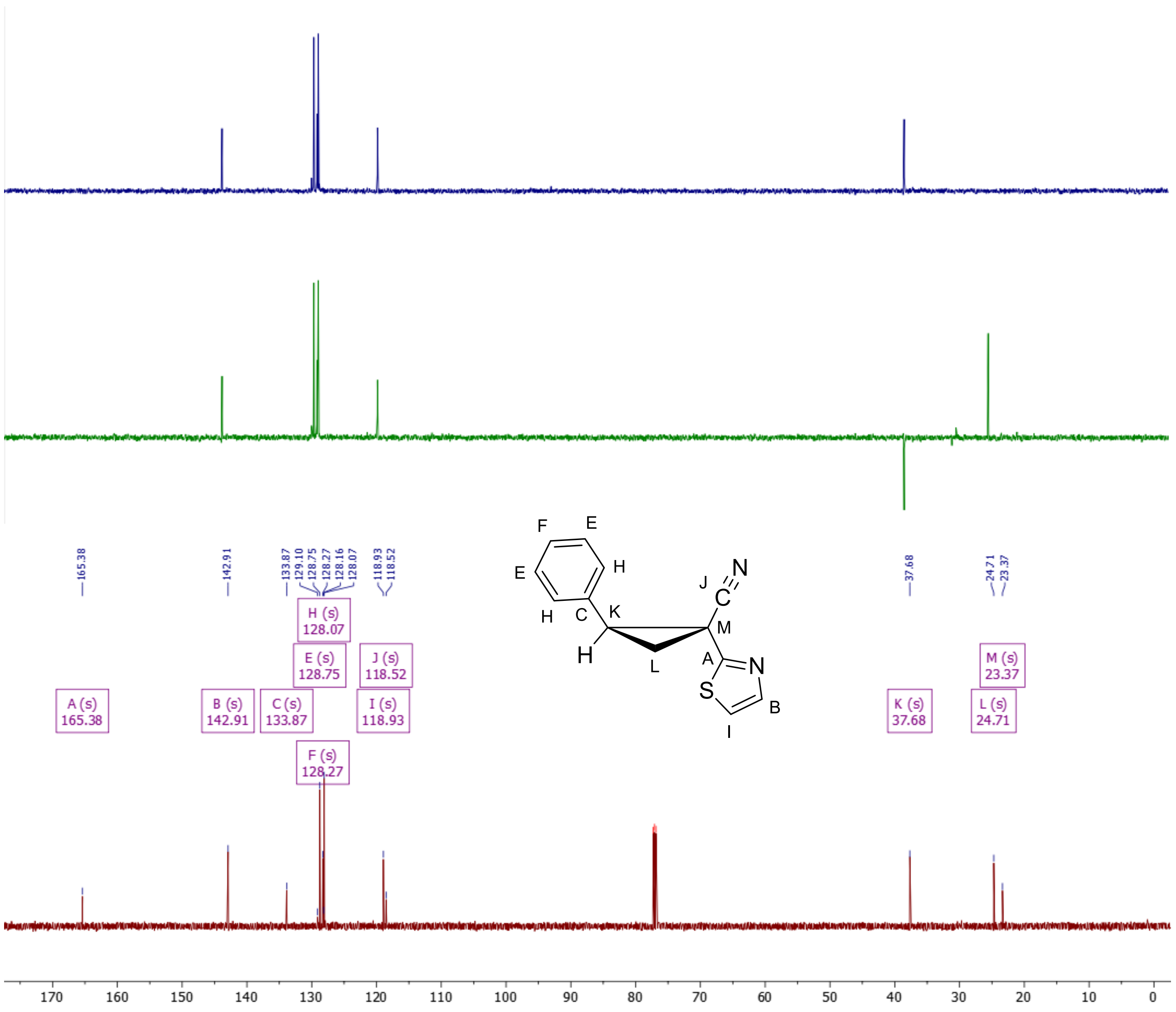

Figure S2. ¹³C NMR and DEPT spectra of **5a**.

The ¹³C NMR spectrum further supports the proposed structure, showing a distinctive signal for carbon A at 165.4 ppm, an uncommon chemical shift for typical carbon environments but characteristic of the C2 position in alkyl-substituted thiazoles. Similarly, carbon I appears in the region usually associated with nitrile carbons; however, it instead corresponds to the C5 position of the thiazole ring (appears as a CH from DEPT), consistent with literature values reported for simpler thiazole derivatives.

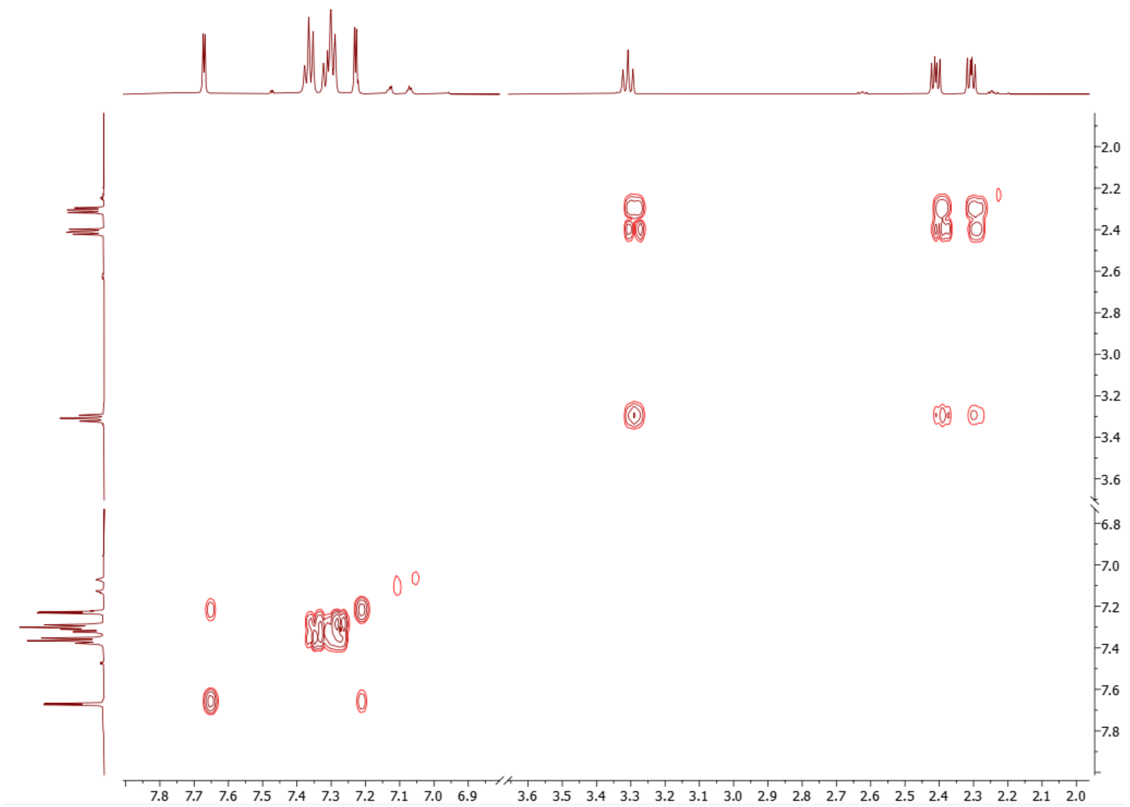

Figure S3. gCOSY of **5a**.

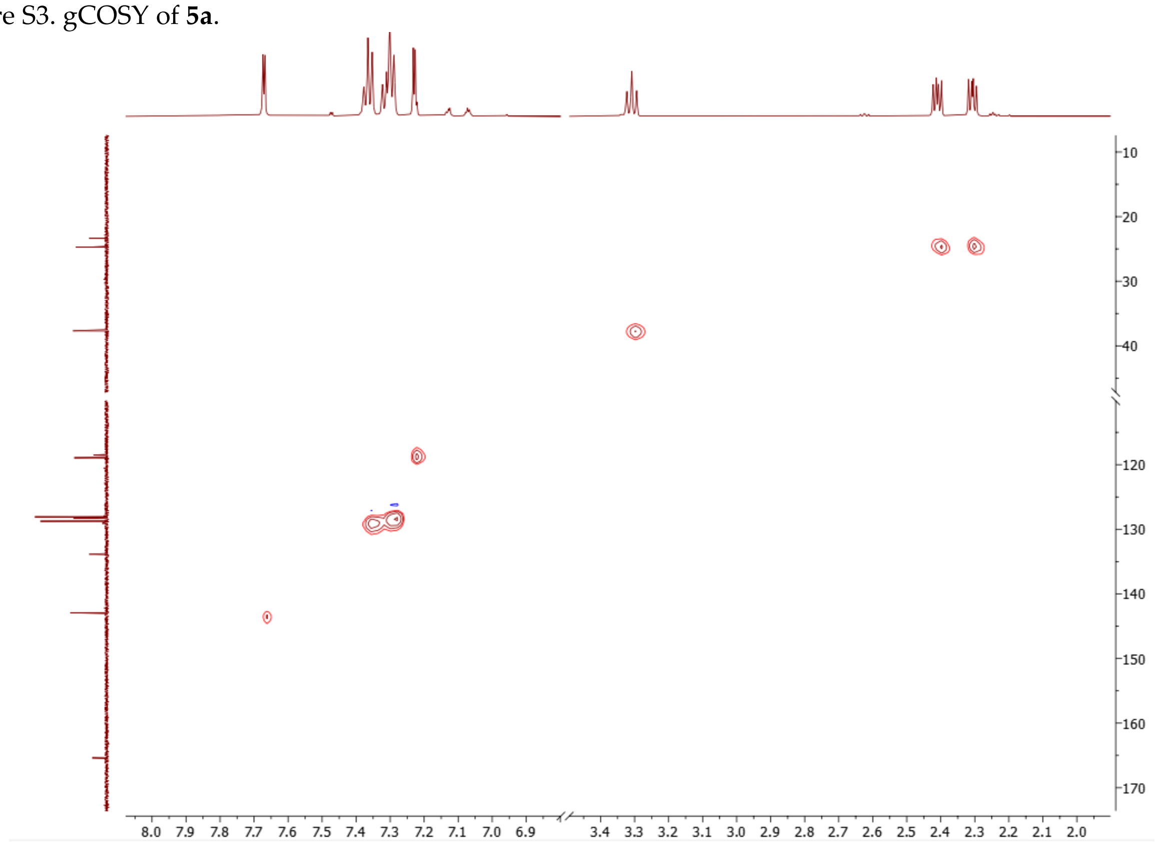

Figure S4. gHSQC of **5a**.

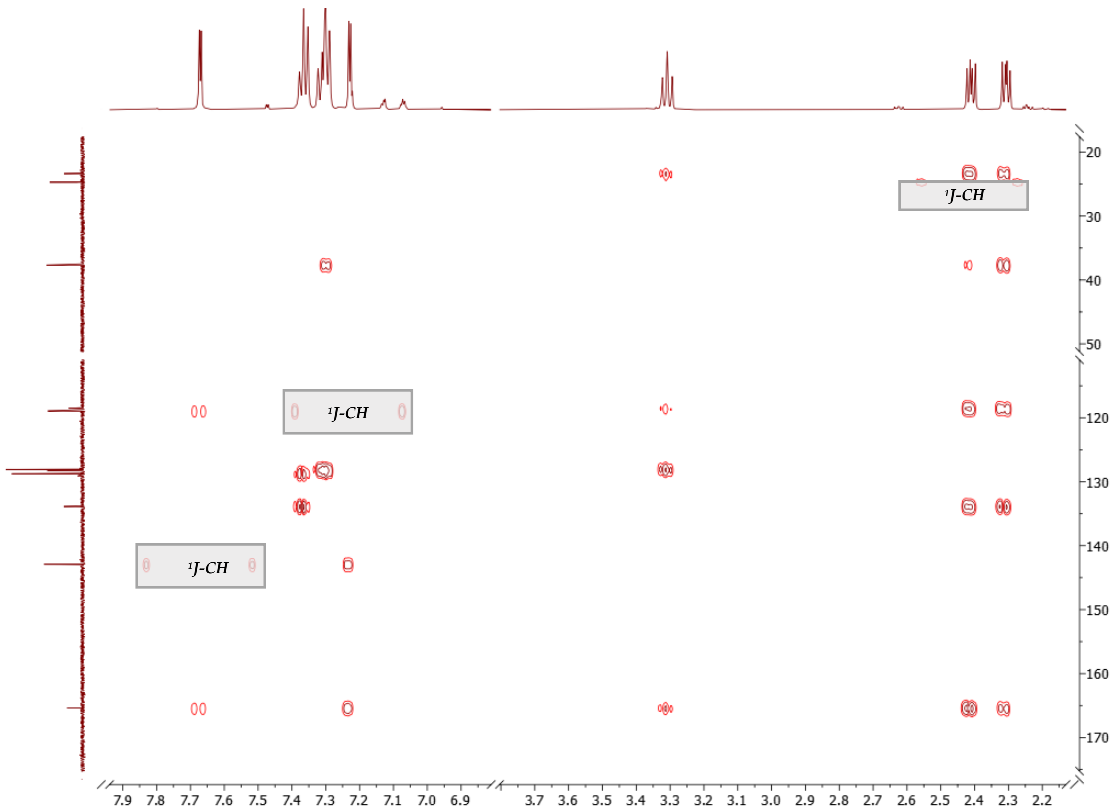

Figure S5. gHMBC of **5a**. Artifacts arising from J[1] couplings are observed and have been masked for clarity.

2D correlation experiments further confirm the expected connectivity, fully consistent with the assignments derived from the previously acquired 1D ^{1}H and ^{13}C NMR spectra.

NOE experiments for the relative stereochemistry determination

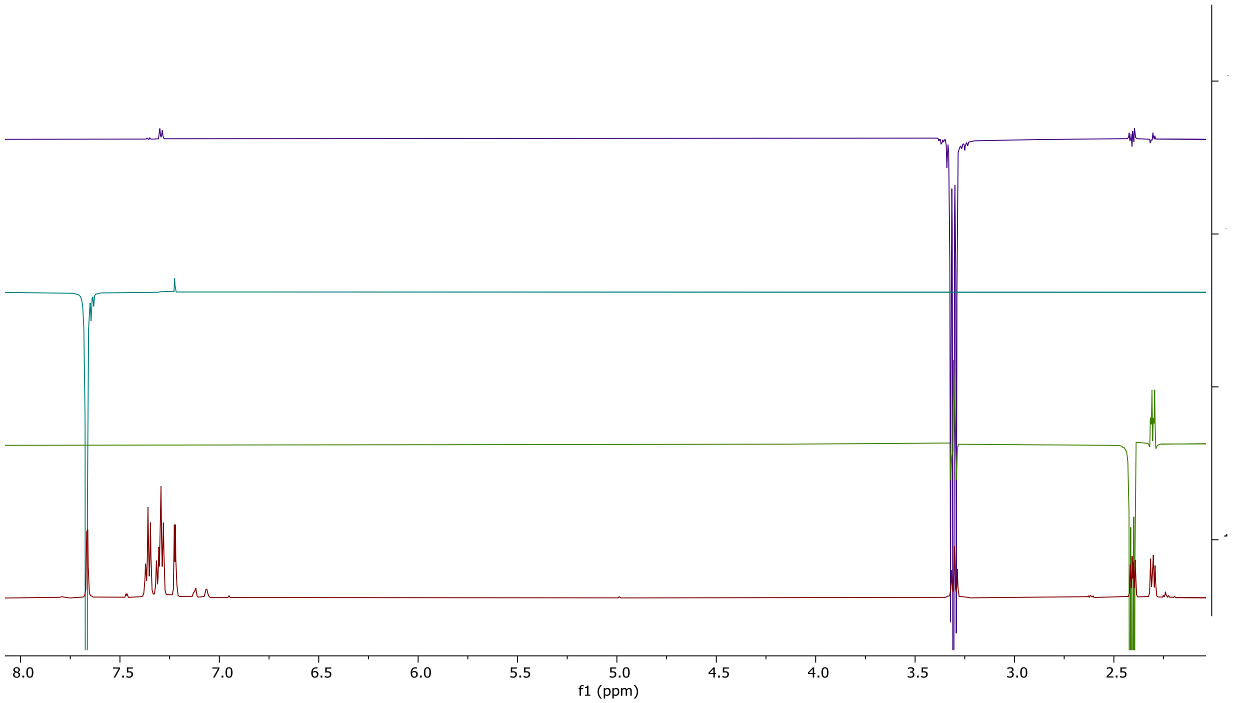

Figure S6. NOE of **5a**.

1D-NOE (Figure S6) experiments carried out directly on **5a** provided confirmation of its structural features. The thiazole protons did not exhibit any observable NOE interactions, consistent with its spatial isolation. NOE correlations among protons A, A′, and B (Figure S7) supported the presence of the cyclopropane moiety, while the interaction between protons E and B (Figure S7) corroborated the connectivity inferred from the 2D NMR spectra. However, these experiments did not provide conclusive information regarding the relative stereochemistry of the two chiral centers.

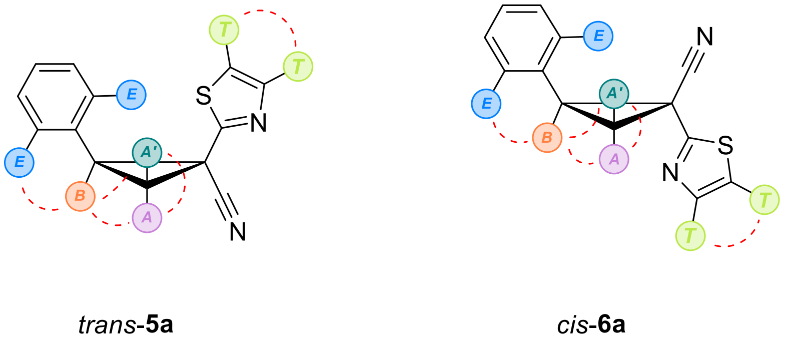

Figure S7. Structure of **5a** and **6a**.

Therefore, in order to gain insight into the relative configuration of product **5a**, we reduced it to the corresponding amine **7a** using borane in THF at 60°C (Scheme S3).

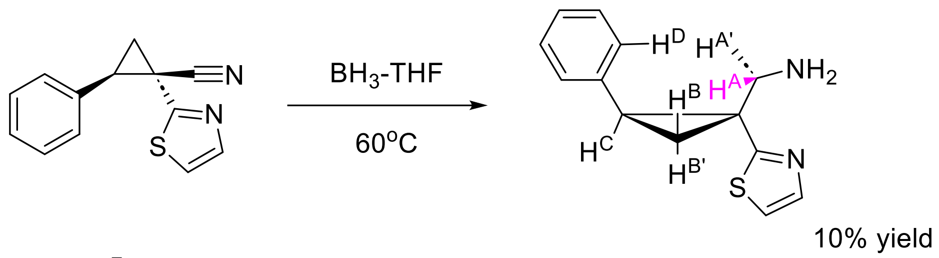

5a **7a**

Scheme S3. Reduction of **5a**

Compound **7a** was then subjected to selective irradiation at one of the two diastereotopic hydrogens (**H_A**) of the methylene group, and the resulting NOE response was monitored (Figure S8)

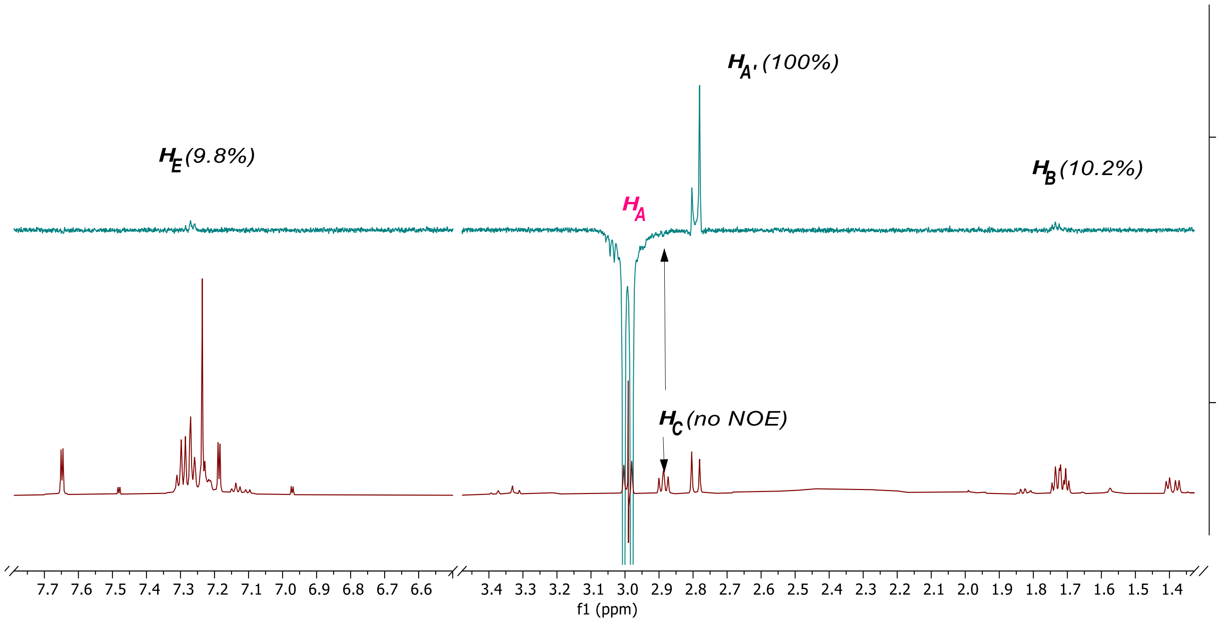

Figure S8. ^{1}H-NMR and NOE spectra of **7a**.

The NOE experiment showed a strong enhancement of $H_{A'}$, which was used as reference signal and set at 100% integral. A corresponding distance of 1.8 Å between H_A and $H_{A'}$ was calculated via DFT, and found to be approximately the same in both diastereoisomers.
Upon irradiation, H_B exhibited a 10.2% enhancement, corresponding to an estimated internuclear distance of 2.5 Å. This value is in good agreement with the 2.3 Å distance obtained from DFT calculations and it is compatible with both diastereoisomeric structures (2.334 Å for *trans*-**7a** and 2.356 Å for *cis*-**7a** respectively, Figure S8).
One of the protons of the phenyl ring exhibited a similar NOE response, with an integral of 9.8%, corresponding to an estimated internuclear distance of 2.7 Å. This observation is in excellent agreement with the DFT-calculated distances for the *trans*-diastereoisomer (*trans*-**7a**, 2.625 Å) and is completely inconsistent with the geometry of the *cis*-diastereoisomer (*cis* -**7a**, 4.387 Å) (Figure S9).

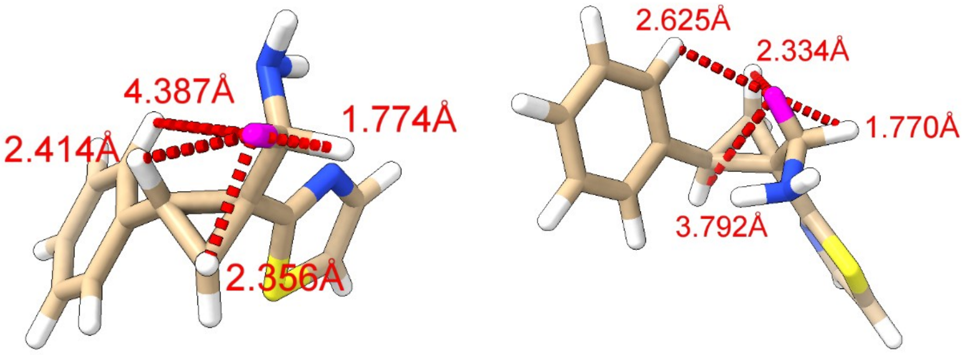

Figure S9. DFT calculated geometries (r2SCAN-3c/D4/def2-mTZVPP)

These results led us to conclude that the major diastereoisomer obtained from our synthesis is *trans*-**4a** that is then dehydrated to the corresponding *trans*- **5a**, in which the two aryl groups are oriented in opposite directions. DFT calculations for the *cis*-diastereoisomer **7a** indeed revealed interproton distances inconsistent with the experimental data, such as an H_A and H_D distance greater than 4.2 Å. Furthermore, no NOE enhancement was observed between H^A and H^C, despite their expected close spatial proximity in the *cis* configuration. A DFT-based mechanistic investigation (Figure S10) reinforced this assignment, showing that the rate-determining step leading to the *trans* products **4** proceeds *via* a significantly lower activation barrier compared to the *cis* pathway.

Computational DFT investigation

All mechanism and geometries calculations were carried out using r²SCAN-3c/D4/def2-mTZVPP in ORCA 6.0.1 [80]. Calculations were conducted without applying any solvation model to the sistem. Harmonic frequency analyses were carried out to confirm the nature of each stationary point – either as a minimum (no imaginary frequencies) or a transition state (one imaginary frequency) – and to compute zero-point energy as well as thermal corrections to enthalpy and Gibbs free energy under standard conditions (298.15 K).

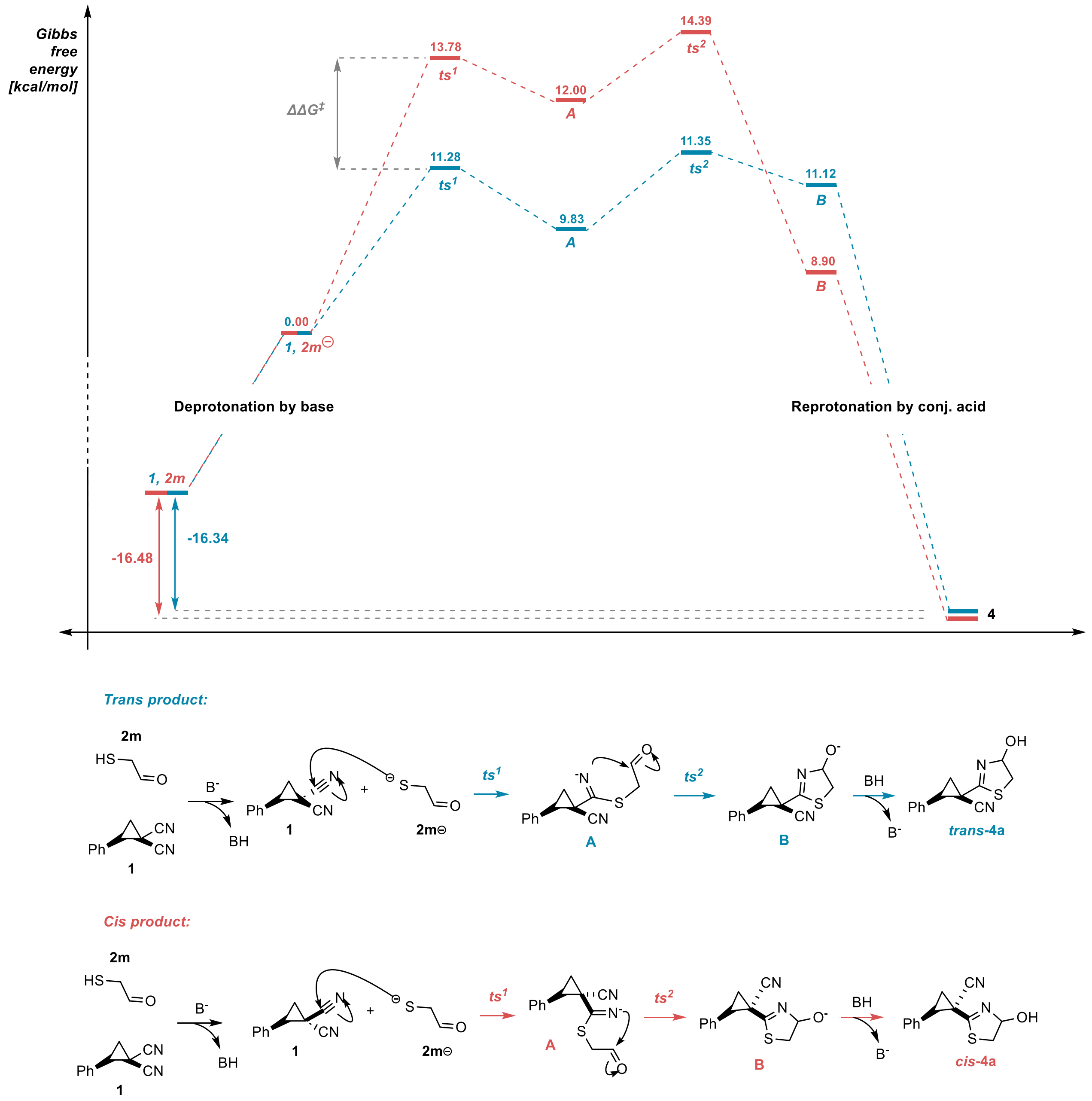

Figure S10. Mechanism elucidation via DFT calculation

The DFT calculations confirm that the proposed reaction mechanism is both reasonable and consistent with the experimental observations. For the *trans* diastereomer, the first transition state (ts^1) exhibits an activation barrier of 11.28 kcal/mol, leading to intermediate A at 9.83 kcal/mol. The reaction then proceeds through a second transition state (ts^2) at 11.35 kcal/mol.
In contrast, the pathway leading to the *cis* diastereomer (*cis*-**4a**) involves significantly higher energy barriers, with the rate-determining transition state $ts^{1'}$ located at 13.78 kcal/mol. The resulting $\Delta\Delta G^{\ddagger}$ between the two competing pathways is approximately 2.5 kcal/mol, rendering the *cis*-selective pathway kinetically disfavored under the studied conditions.

The calculated diastereomeric excess of approximately 98%, derived from $\Delta\Delta G^{\ddagger}$ values using the Eyring equation under the assumption of purely kinetic control, is in excellent agreement with the >20:1 experimentally observed diastereomeric ratio (>95% trans) for product **4a**.
Moreover, all attempts to identify a concerted single-step transition state for the nucleophilic addition were unsuccessful, as no such structure could be located computationally. This further supports a stepwise reaction mechanism proceeding through a discrete intermediate.

Spectra of synthesized products

1-(4-hydroxy-4,5-dihydrothiazol-2-yl)-2-phenylcyclopropane-1-carbonitrile 4a

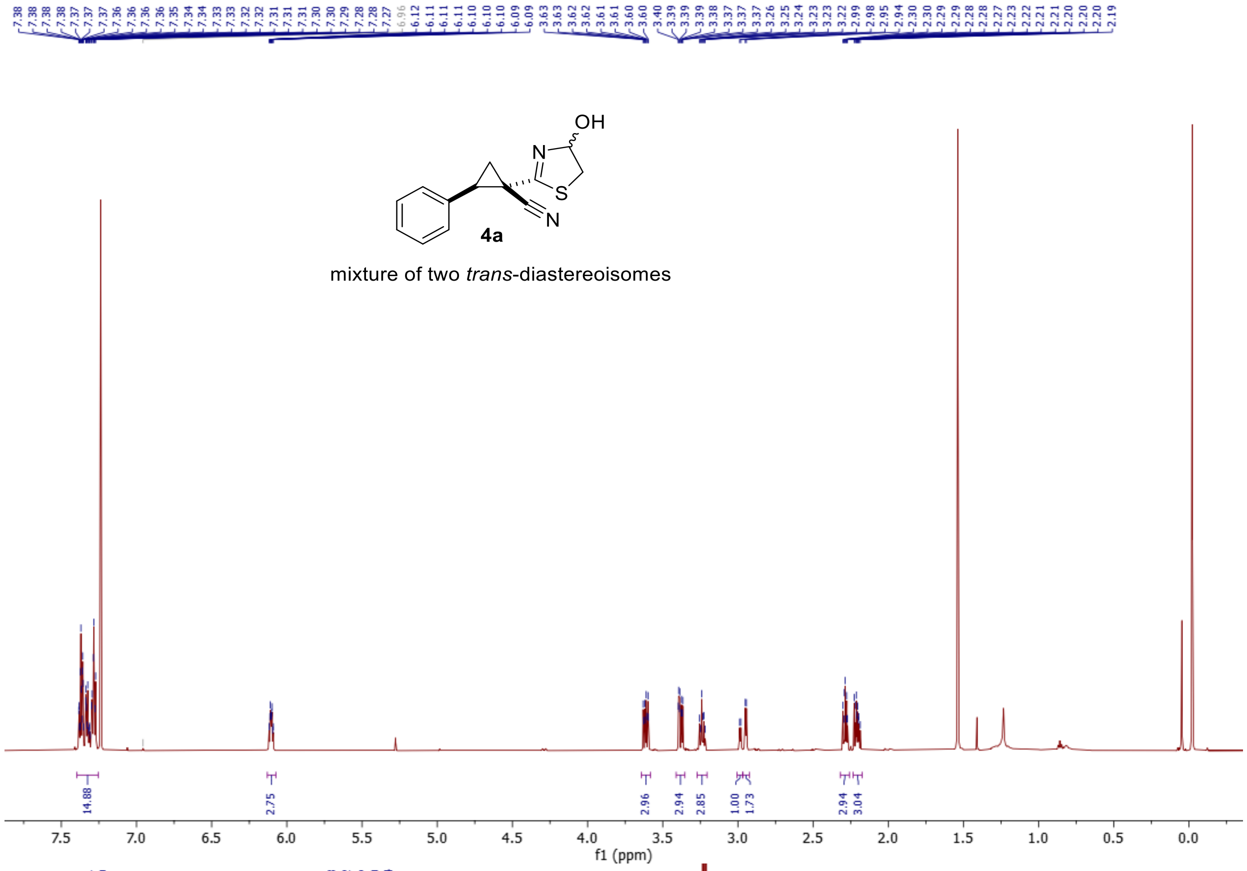

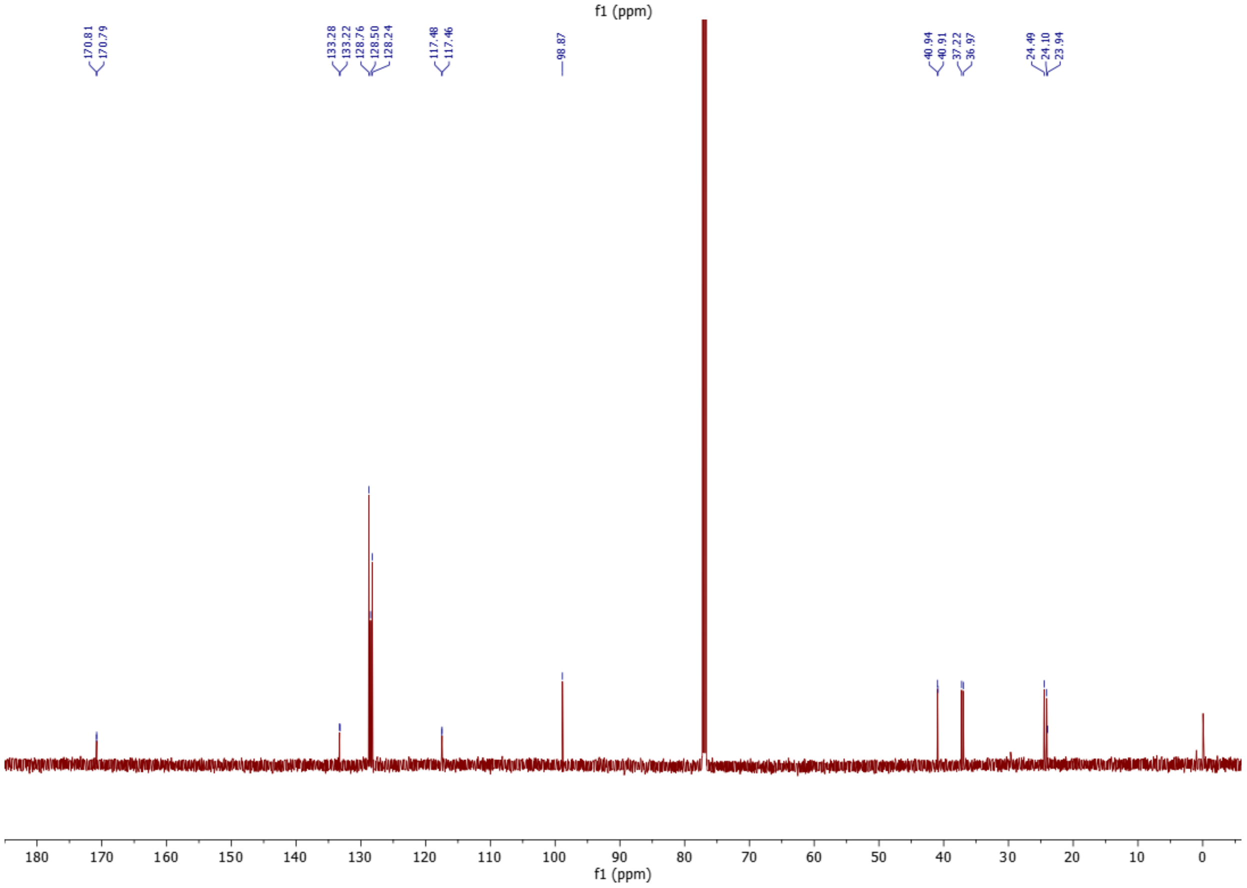

trans-2-phenyl-1-(thiazol-2-yl)cyclopropane-1-carbonitrile 5a

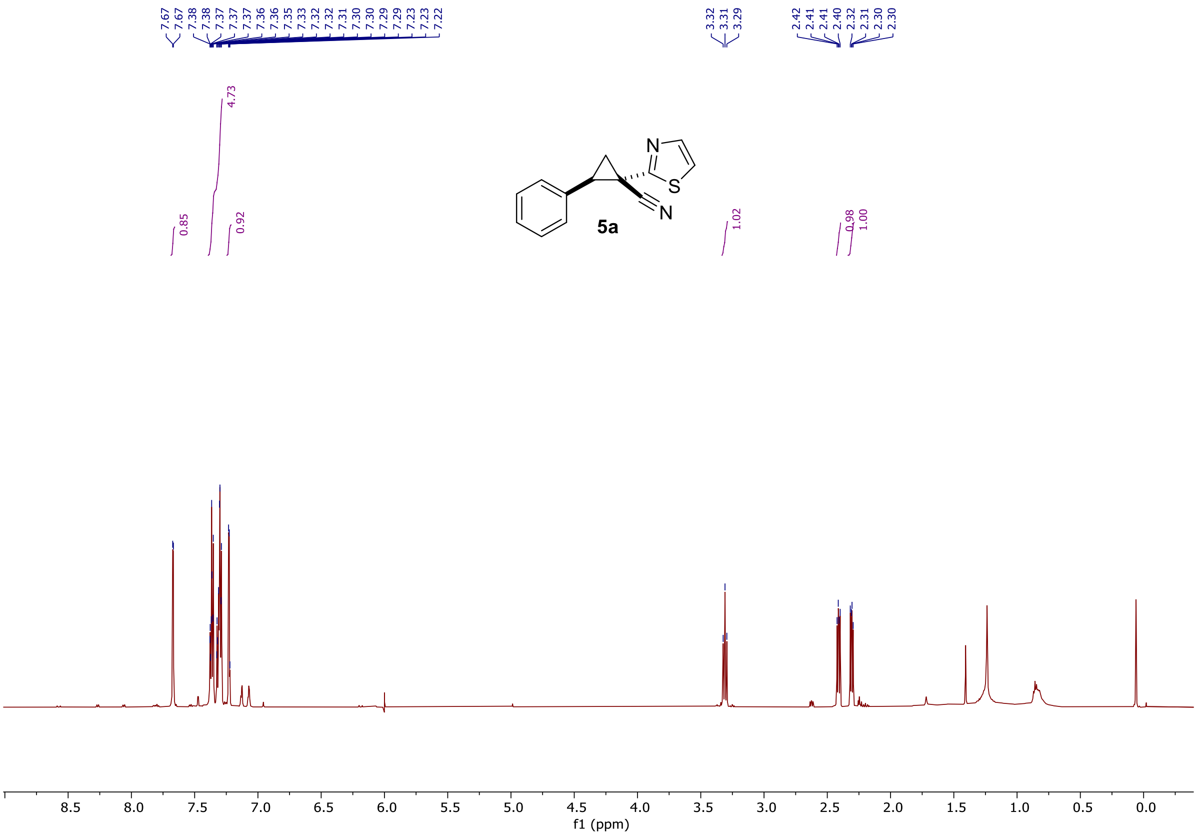

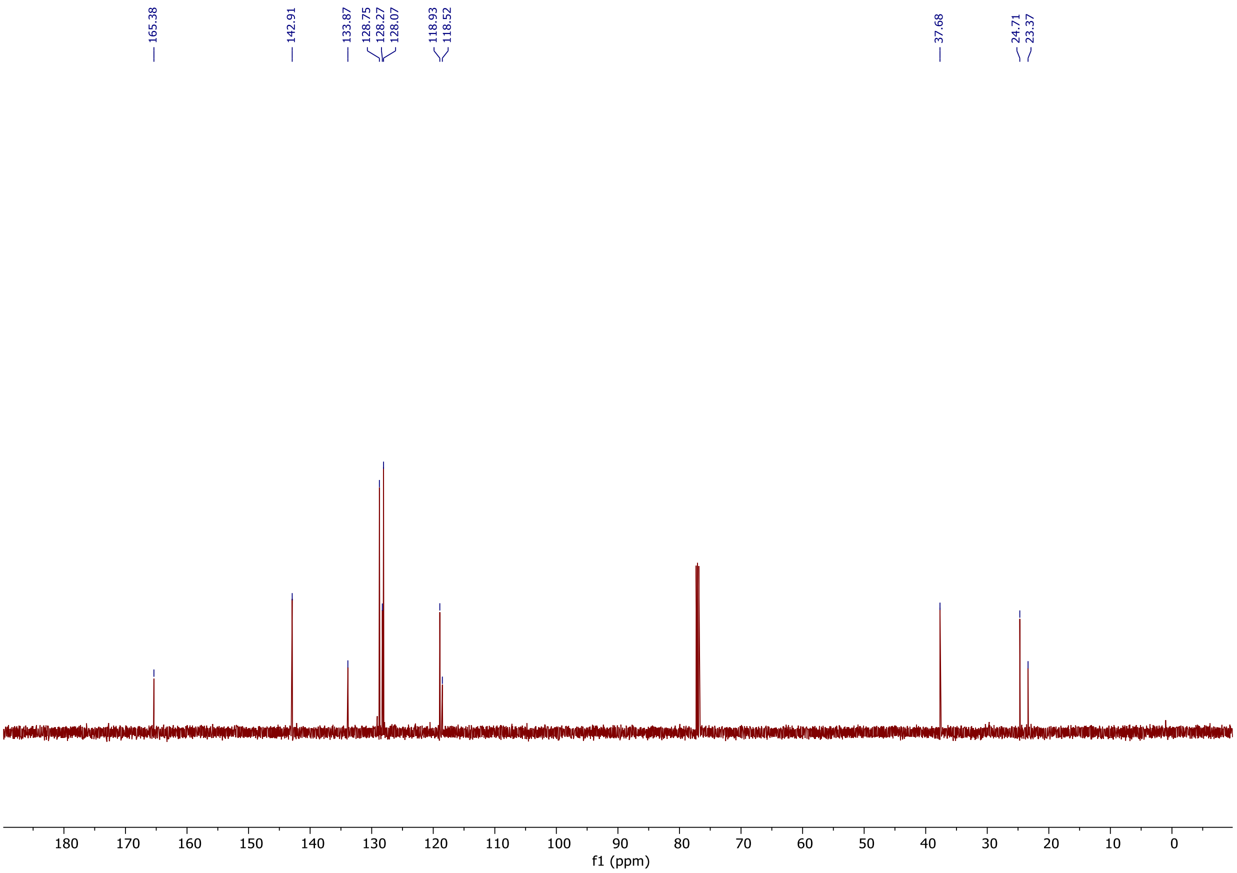

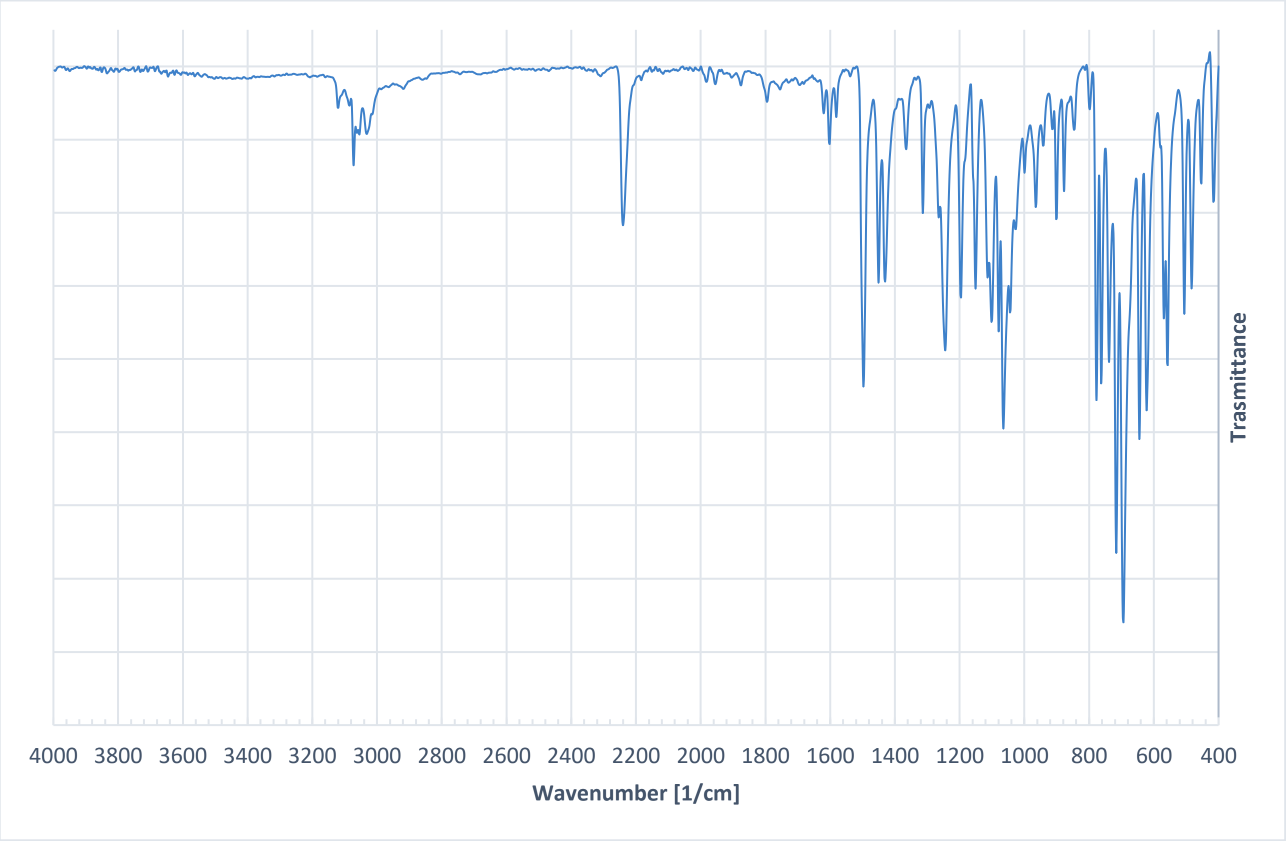
4000 3800 3600 3400 3200 3000 2800 2600 2400 2200 2000 1800 1600 1400 1200 1000 800 600 400
Wavenumber [1/cm]
Trasmittance

trans-(4-fluorophenyl)-1-(thiazol-2-yl)cyclopropane-1-carbonitrile 5b

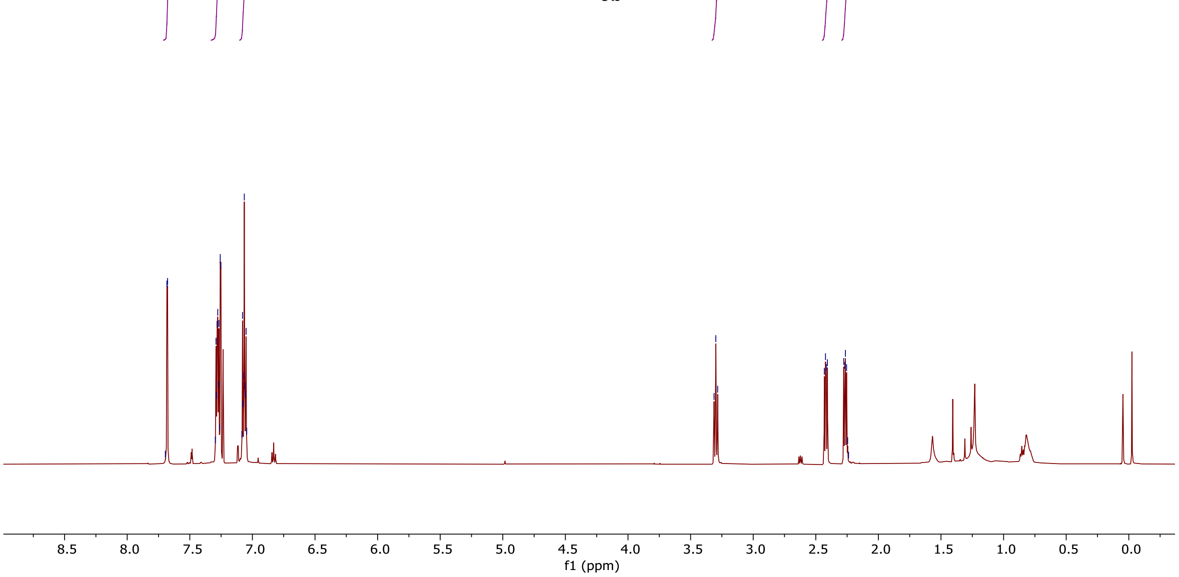

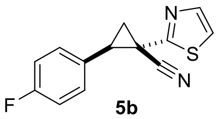

5b

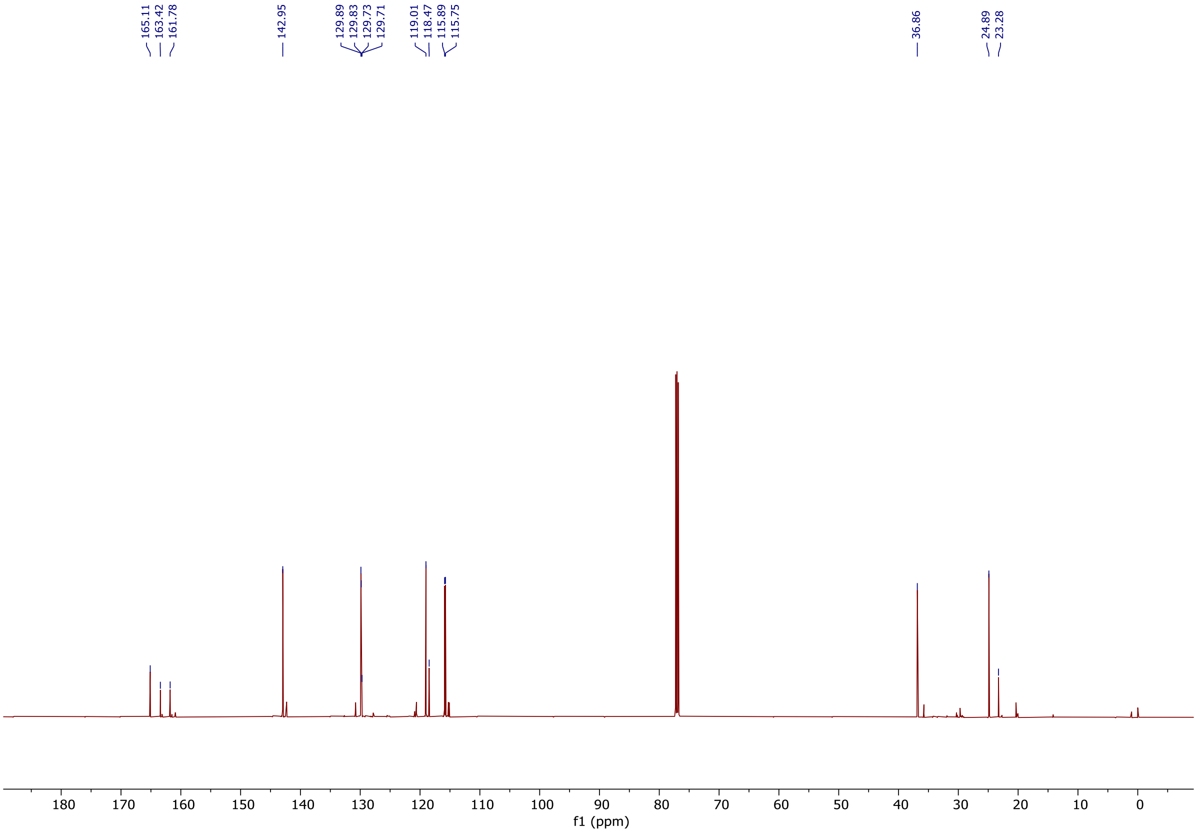

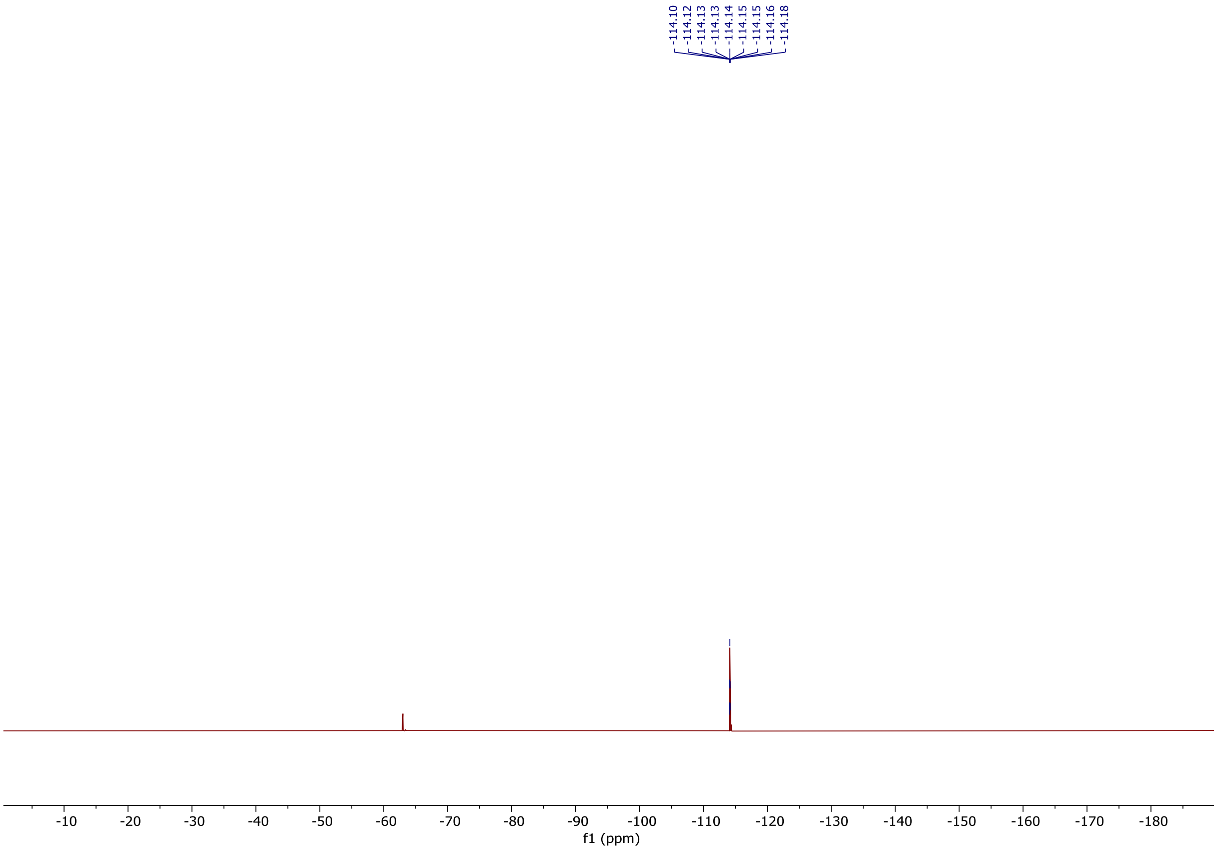
-114.10
-114.12
-114.13
-114.13
-114.14
-114.15
-114.15
-114.16
-114.18
-10
-20
-30
-40
-50
-60
-70
-80
-90
-100
-110
-120
-130
-140
-150
-160
-170
-180
f1 (ppm)

trans-2-(4-chlorophenyl)-1-(thiazol-2-yl)cyclopropane-1-carbonitrile 5c

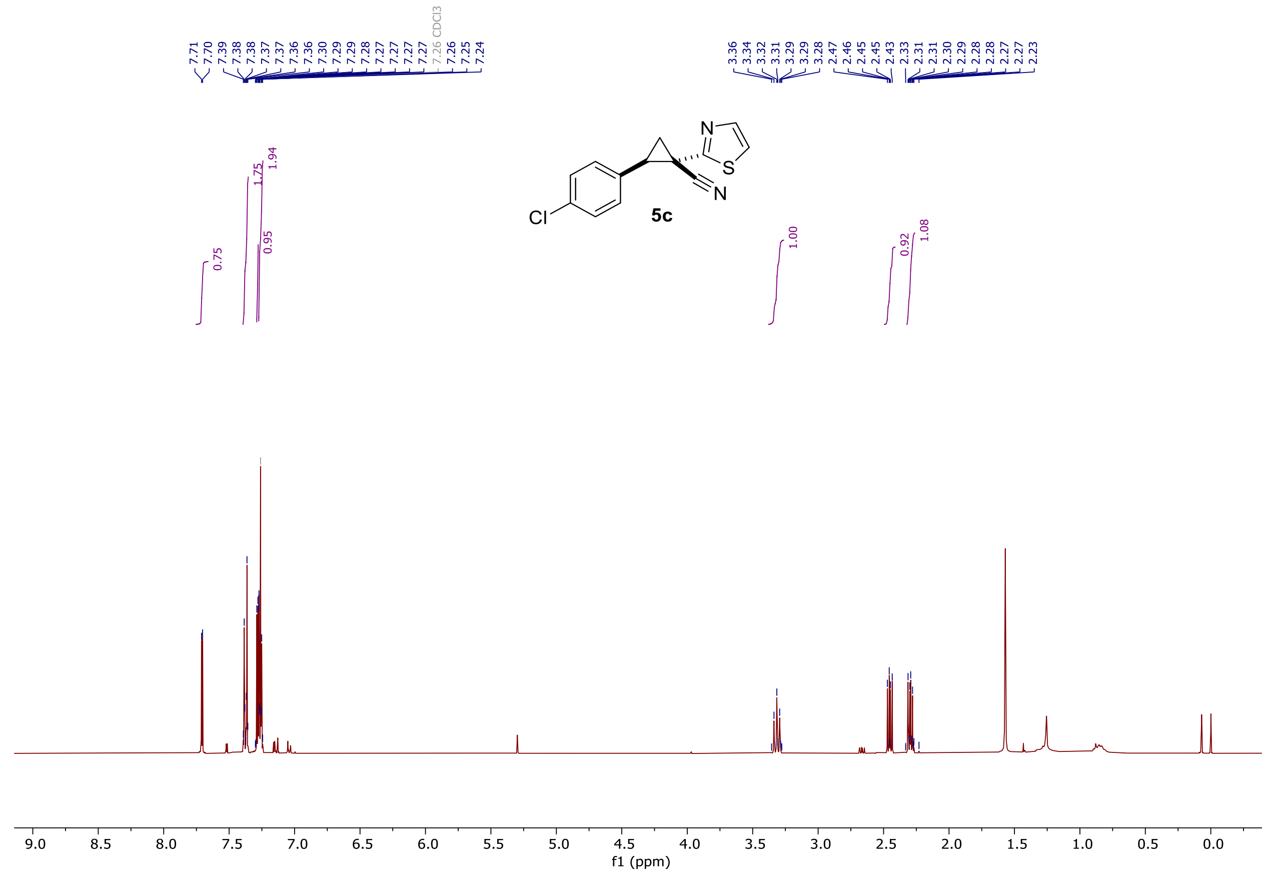

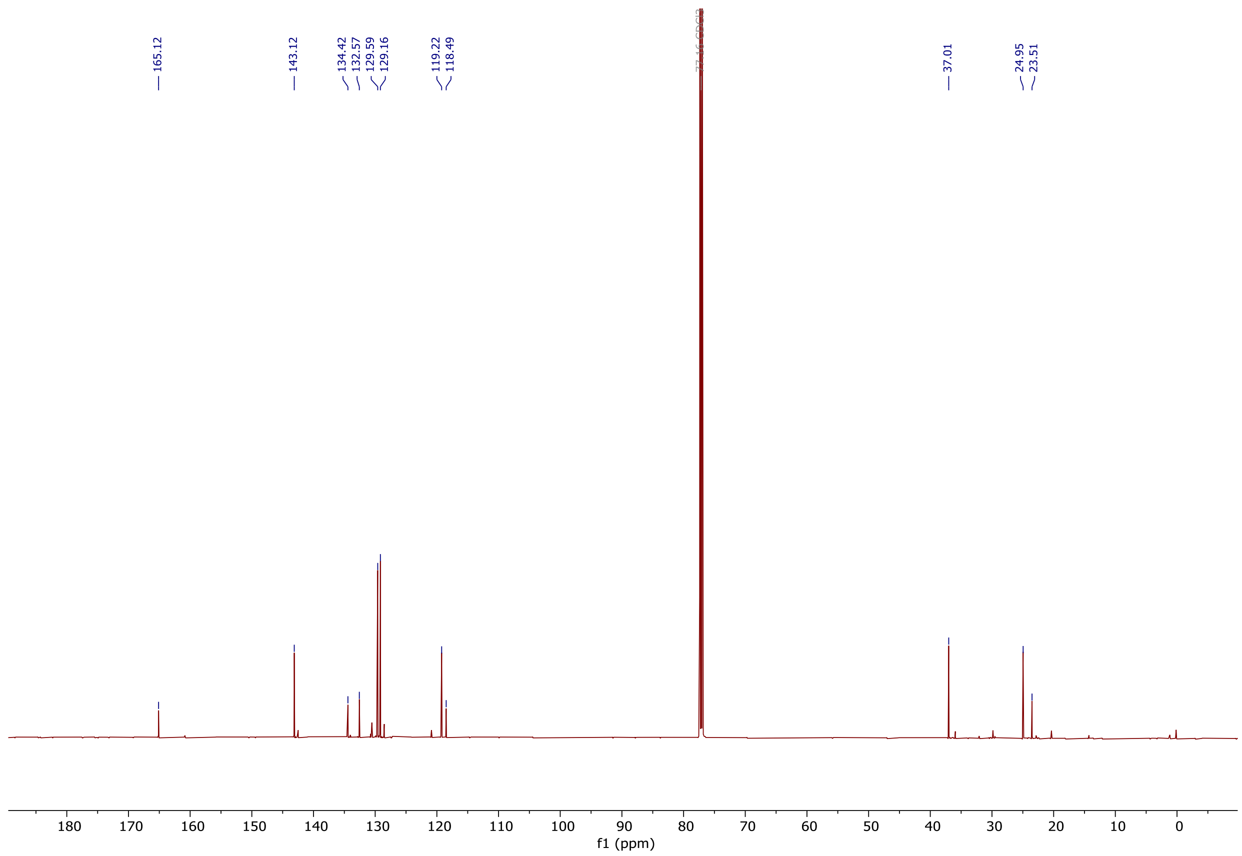

trans-2-(4-bromophenyl)-1-(thiazol-2-yl)cyclopropane-1-carbonitrile 5d

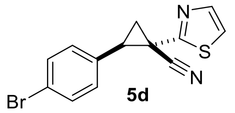

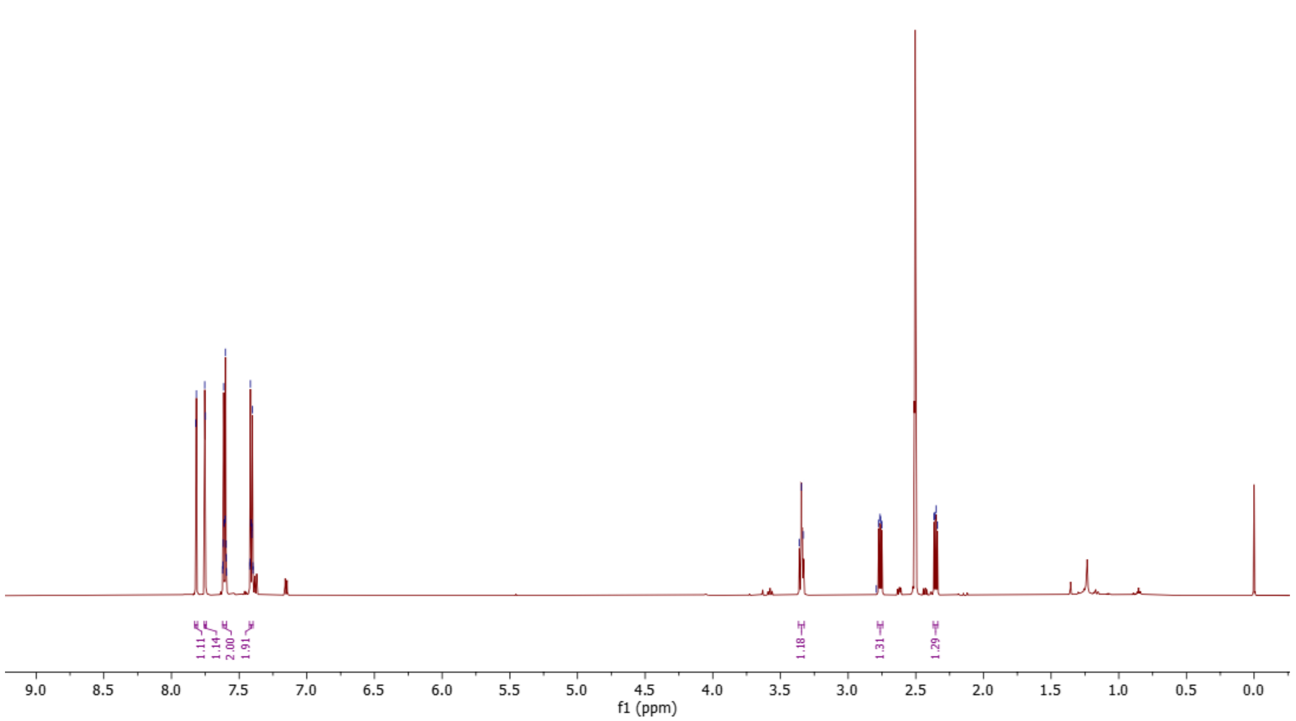

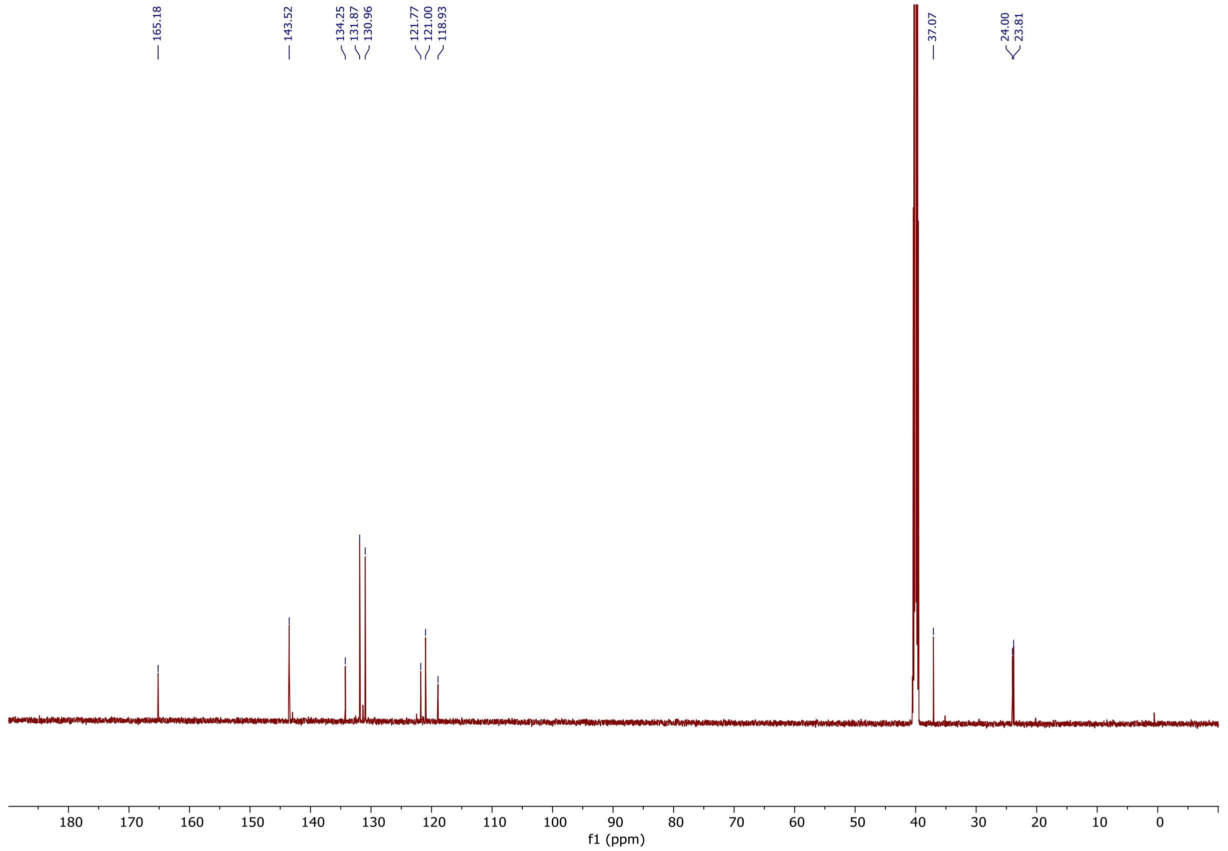

trans-2-(4-iodophenyl)-1-(thiazol-2-yl)cyclopropane-1-carbonitrile 5e

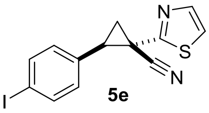

5e

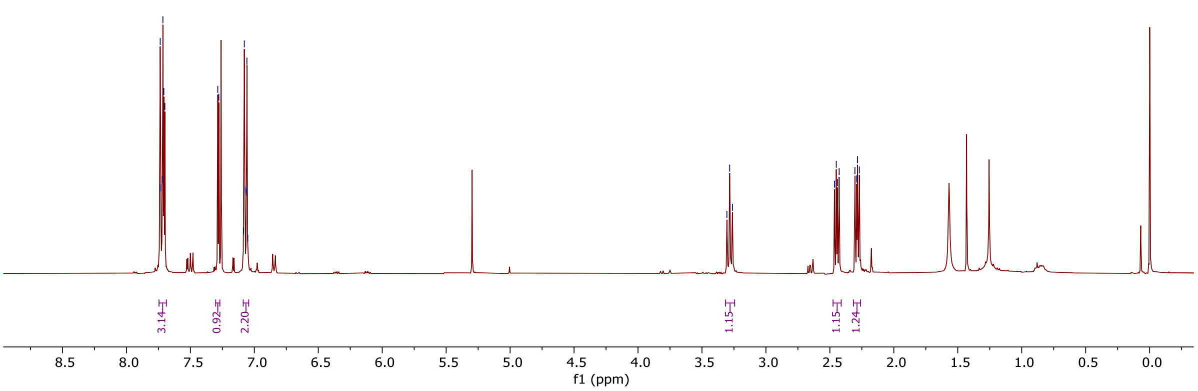

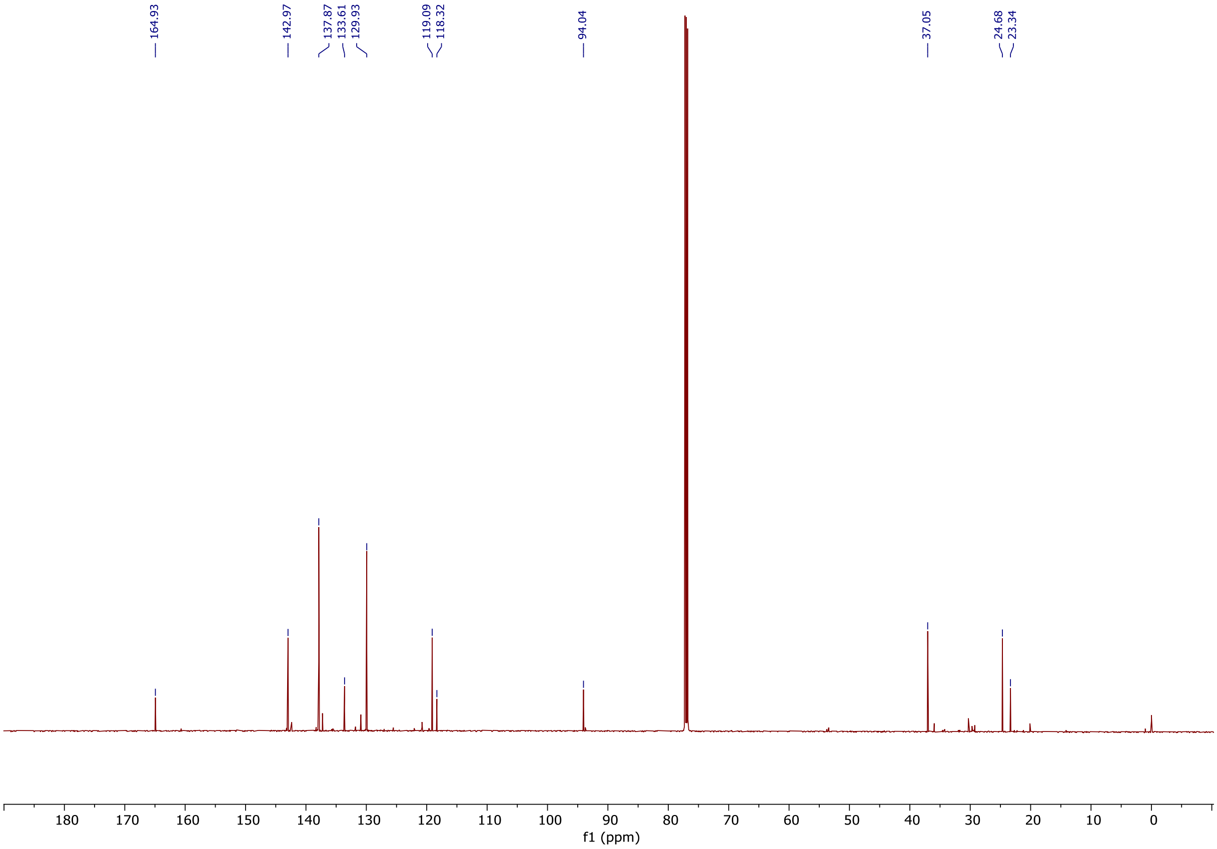

trans-2-(2-chlorophenyl)-1-(thiazol-2-yl)cyclopropane-1-carbonitrile 5f

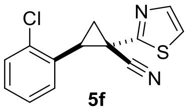

5f

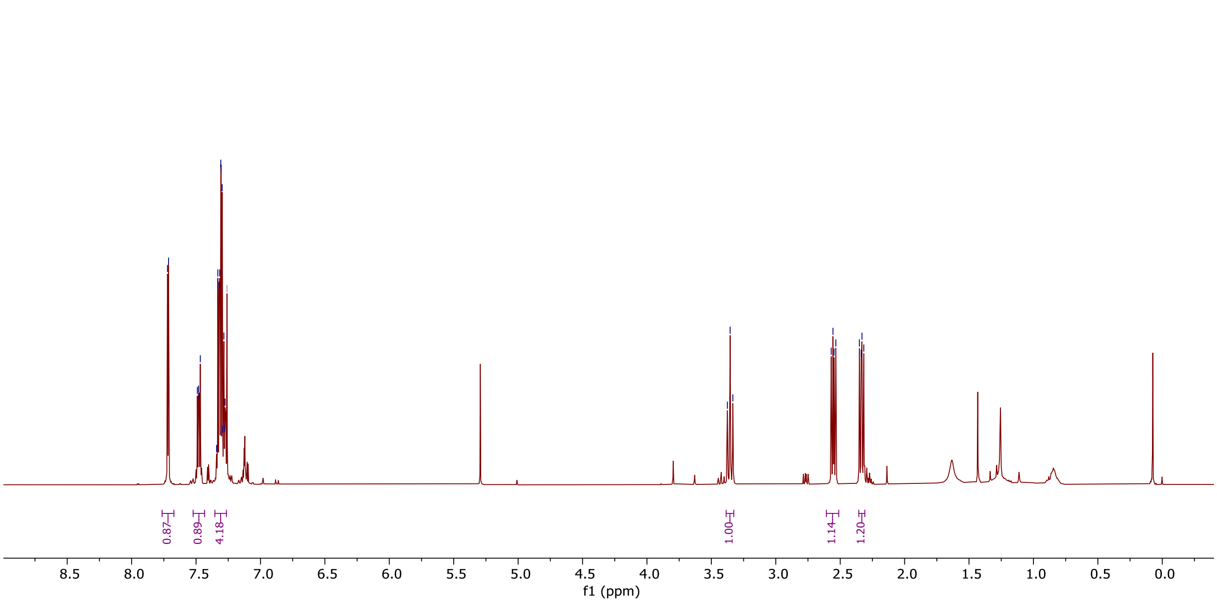

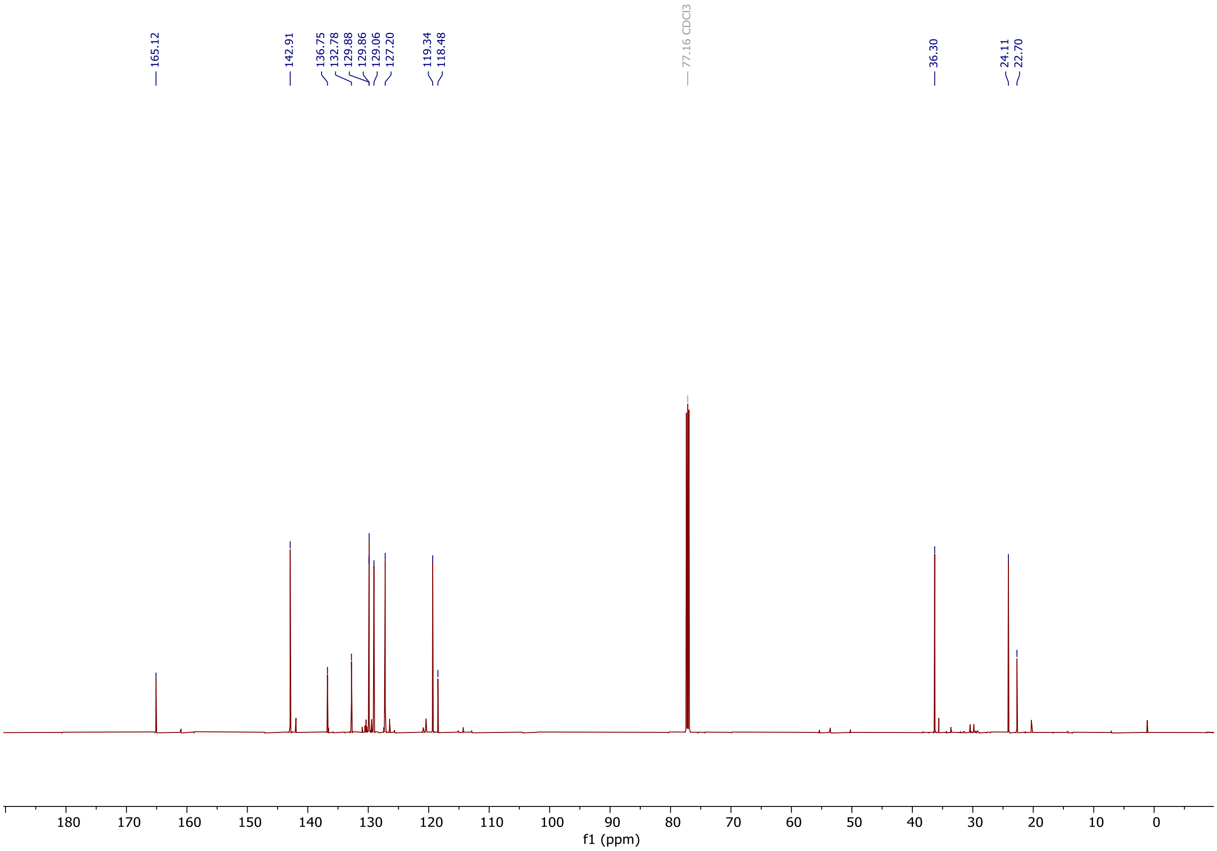

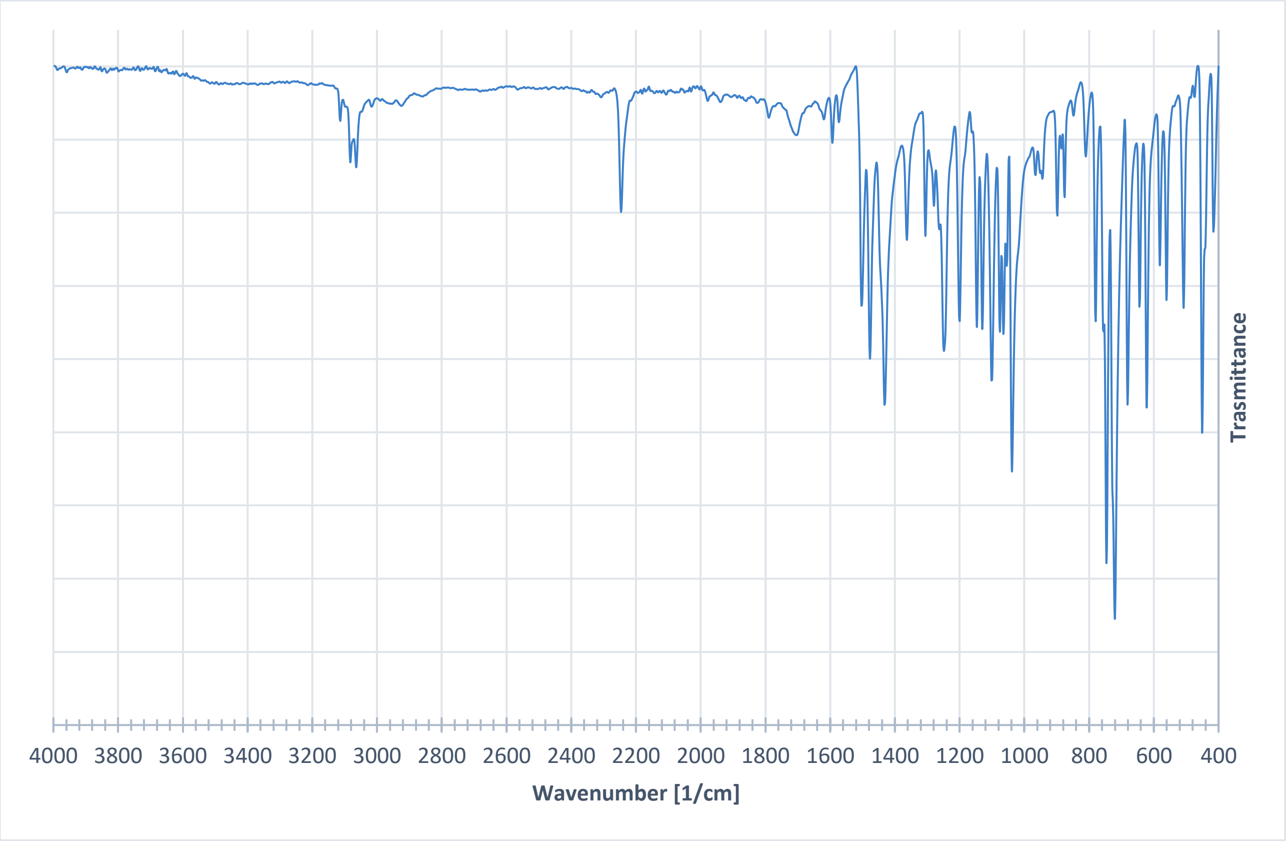
Trasmittance
4000 3800 3600 3400 3200 3000 2800 2600 2400 2200 2000 1800 1600 1400 1200 1000 800 600 400
Wavenumber [1/cm]

trans-2-(3-chlorophenyl)-1-(thiazol-2-yl)cyclopropane-1-carbonitrile 5g

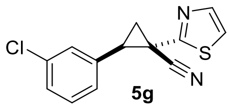

5g

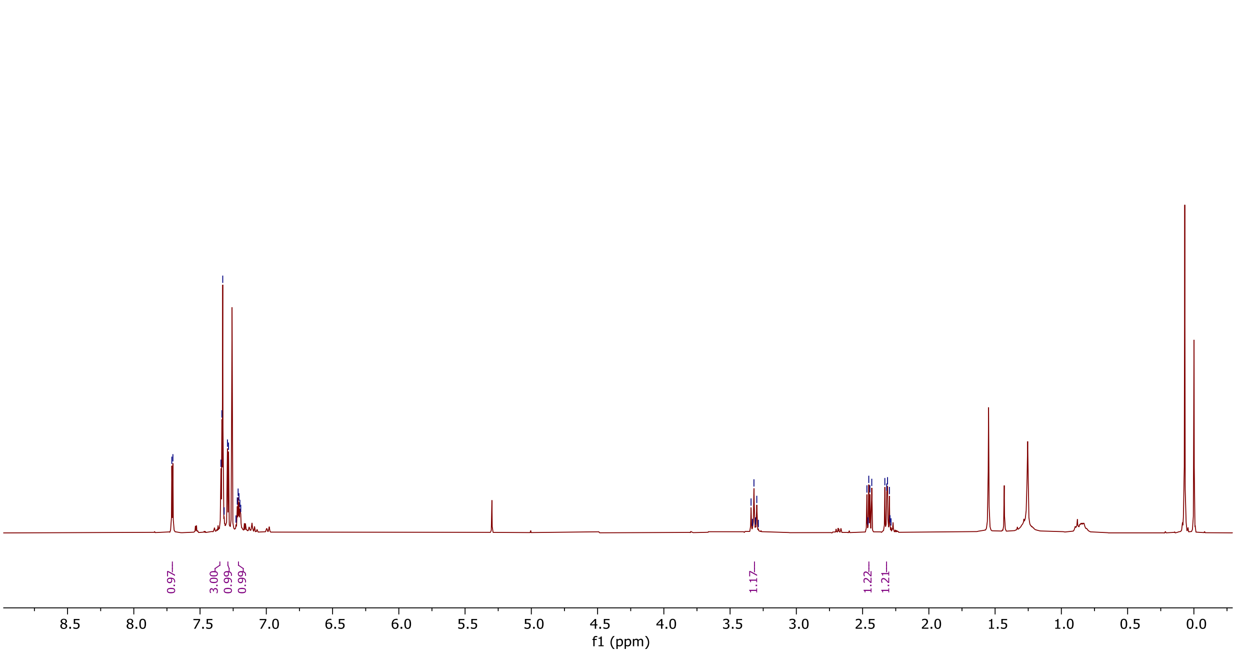

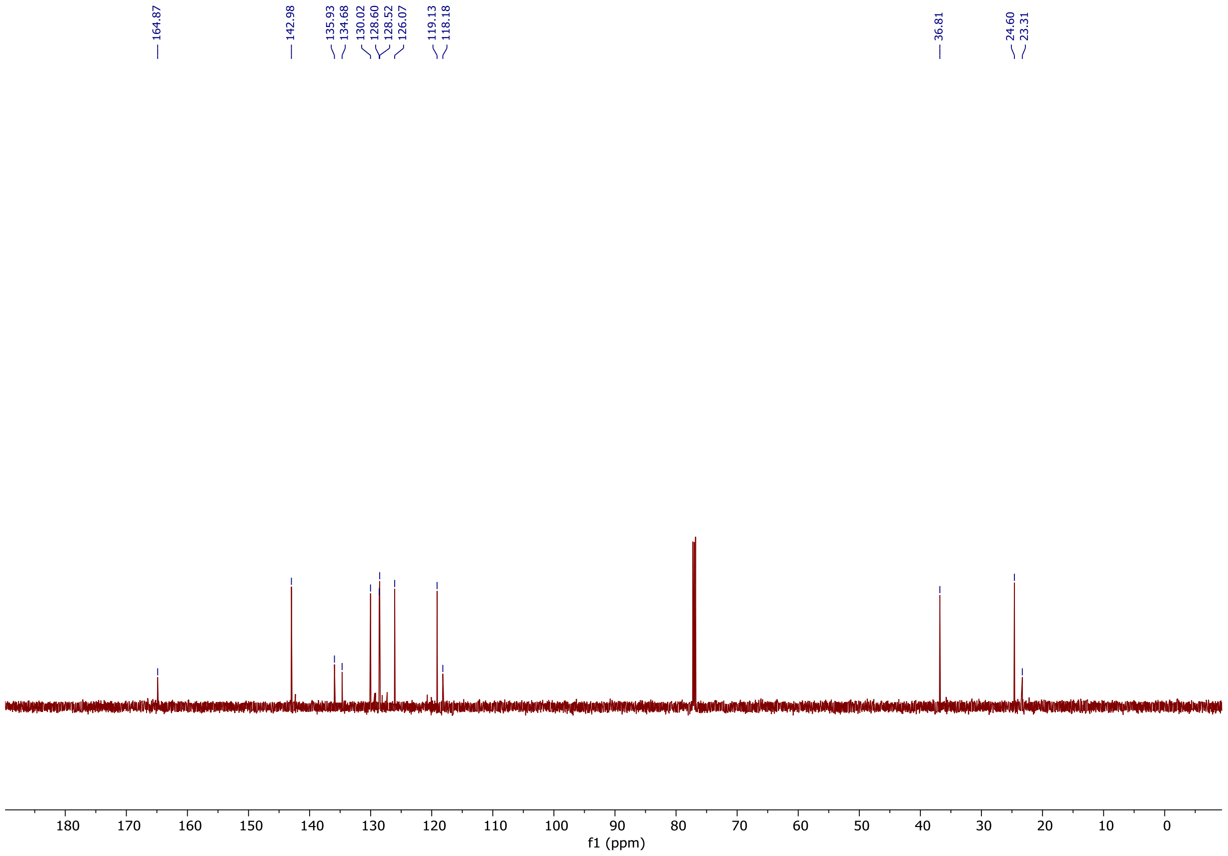

***trans*-2-(2-bromophenyl)-1-(thiazol-2-yl)cyclopropane-1-carbonitrile 5h**

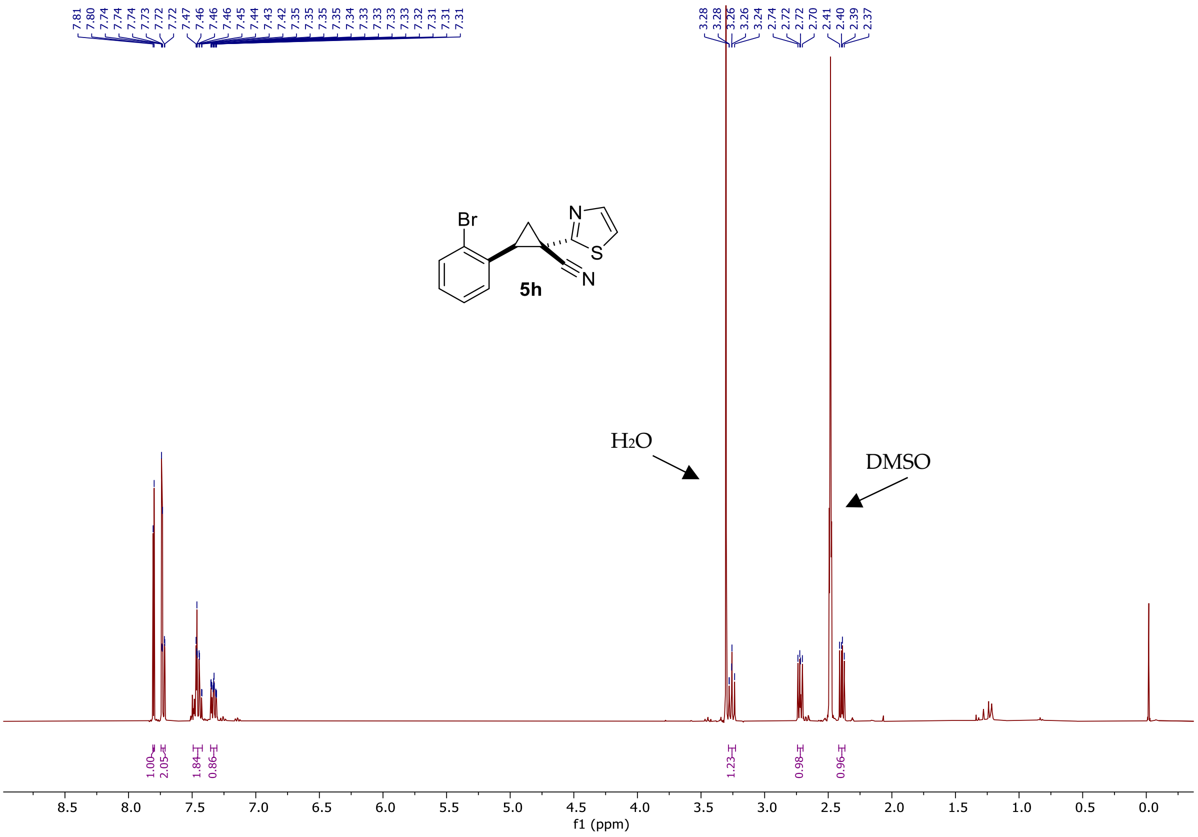

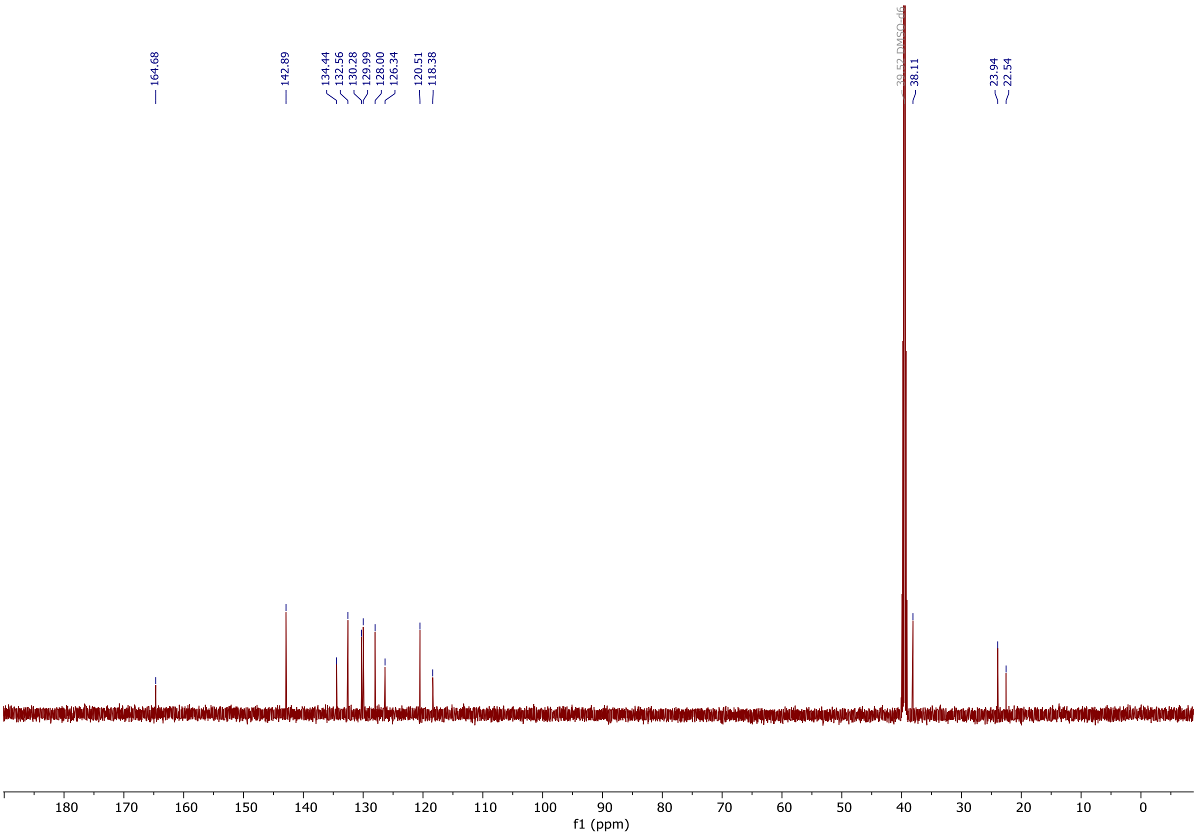

***trans*-2-(4-cyanophenyl)-1-(thiazol-2-yl)cyclopropane-1-carbonitrile 5i**

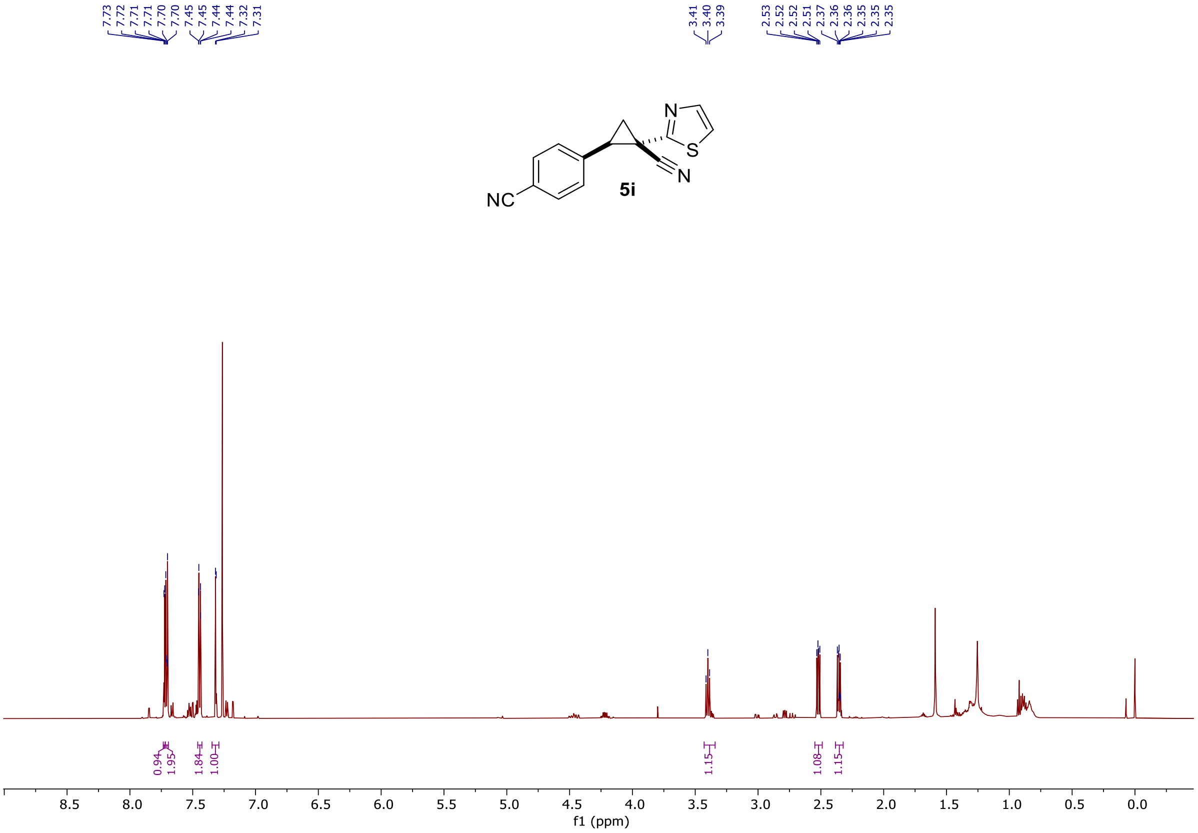

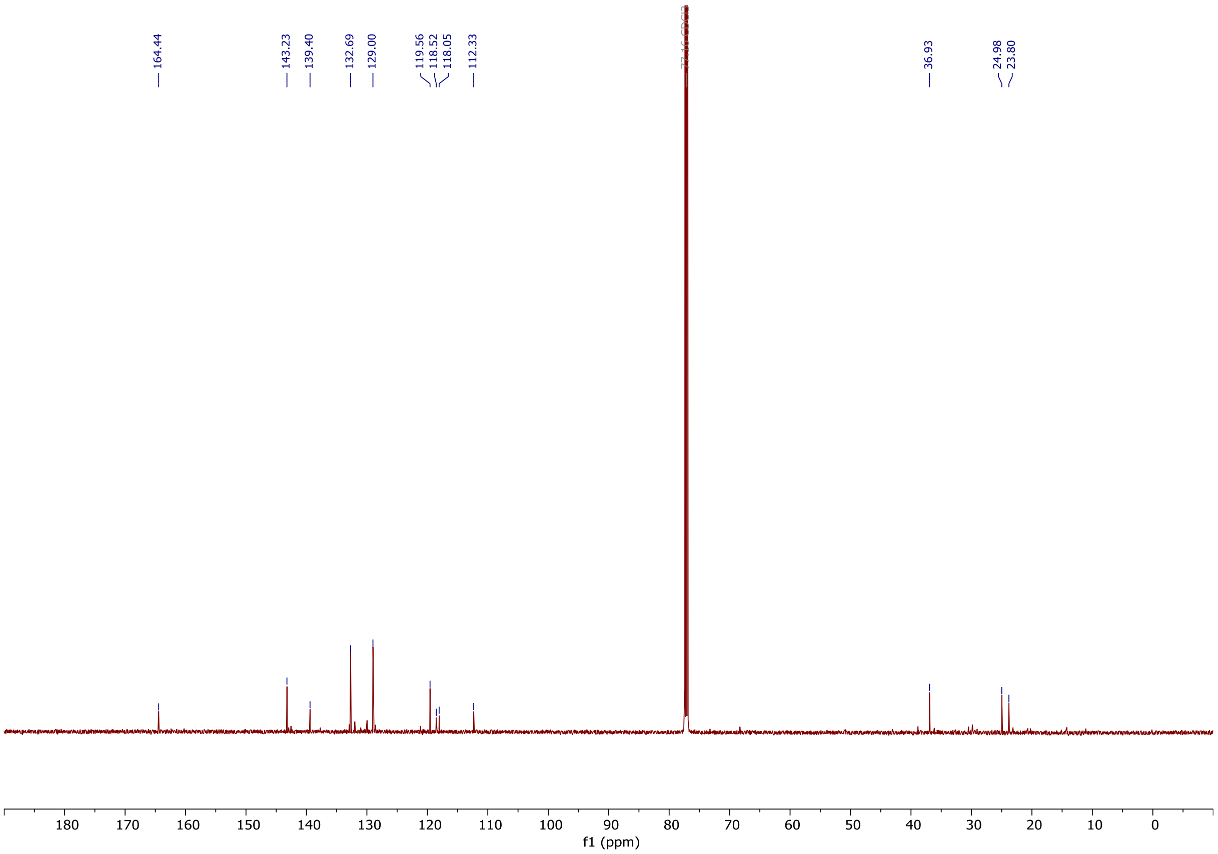

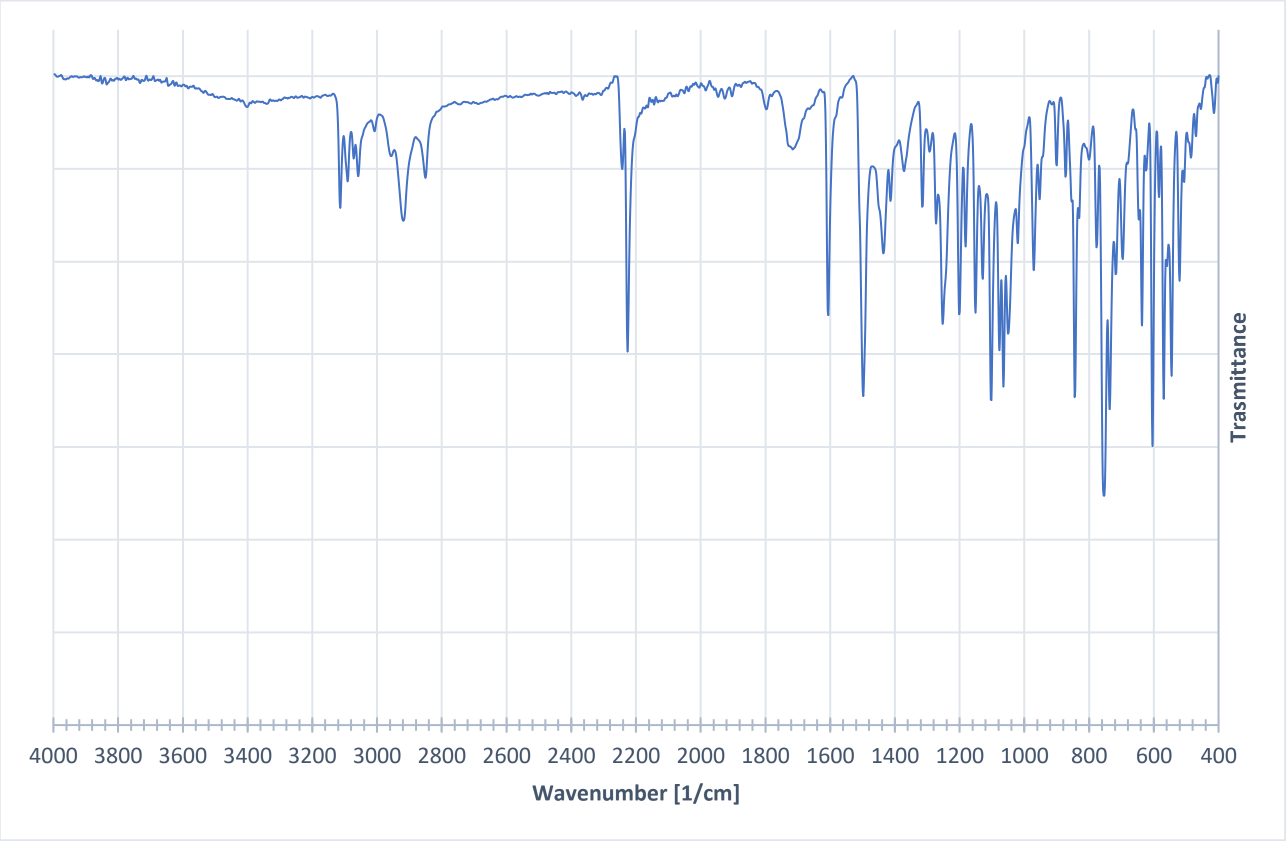
Trasmittance
4000 3800 3600 3400 3200 3000 2800 2600 2400 2200 2000 1800 1600 1400 1200 1000 800 600 400
Wavenumber [1/cm]

***trans*-2-(4-trifluoromethylphenyl)-1-(thiazol-2-yl)cyclopropane-1-carbonitrile 5j**

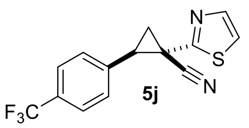

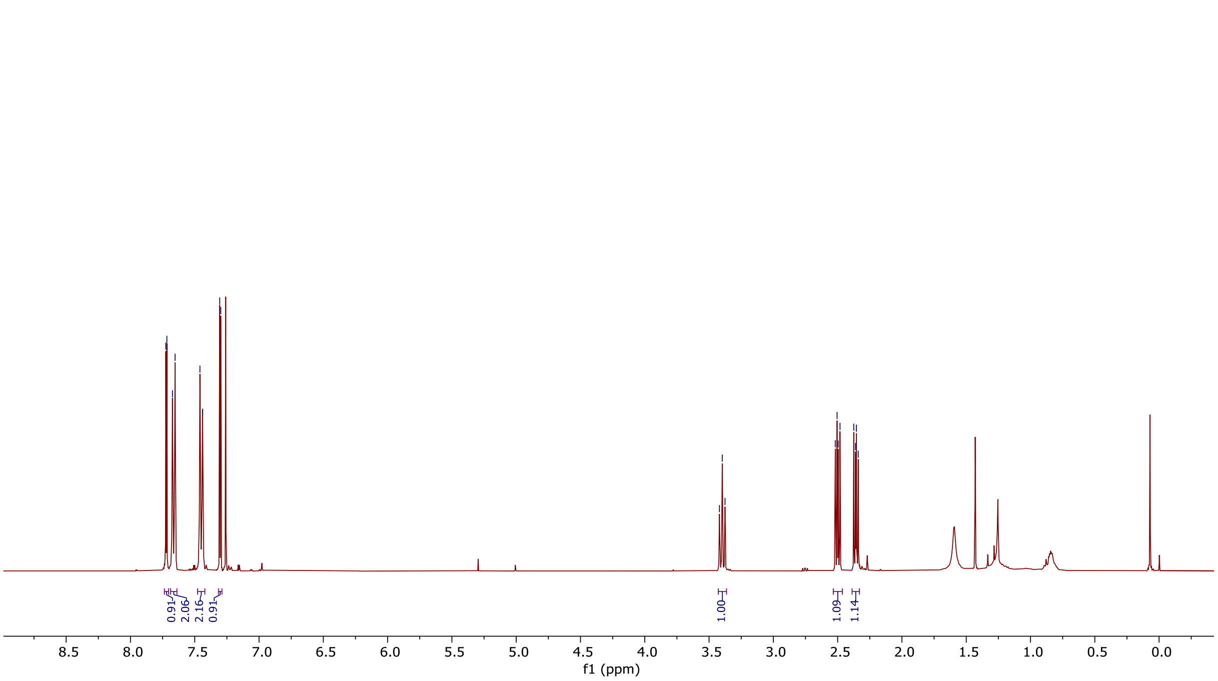

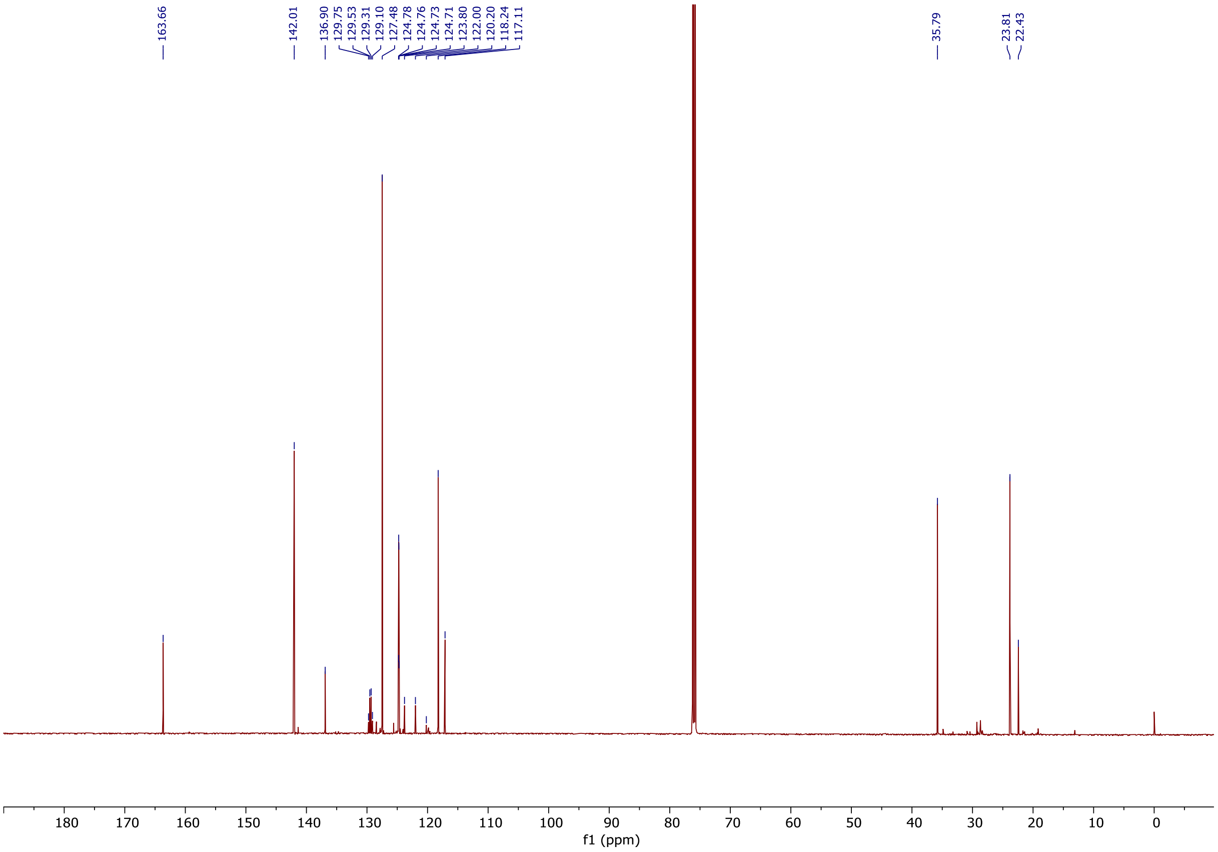

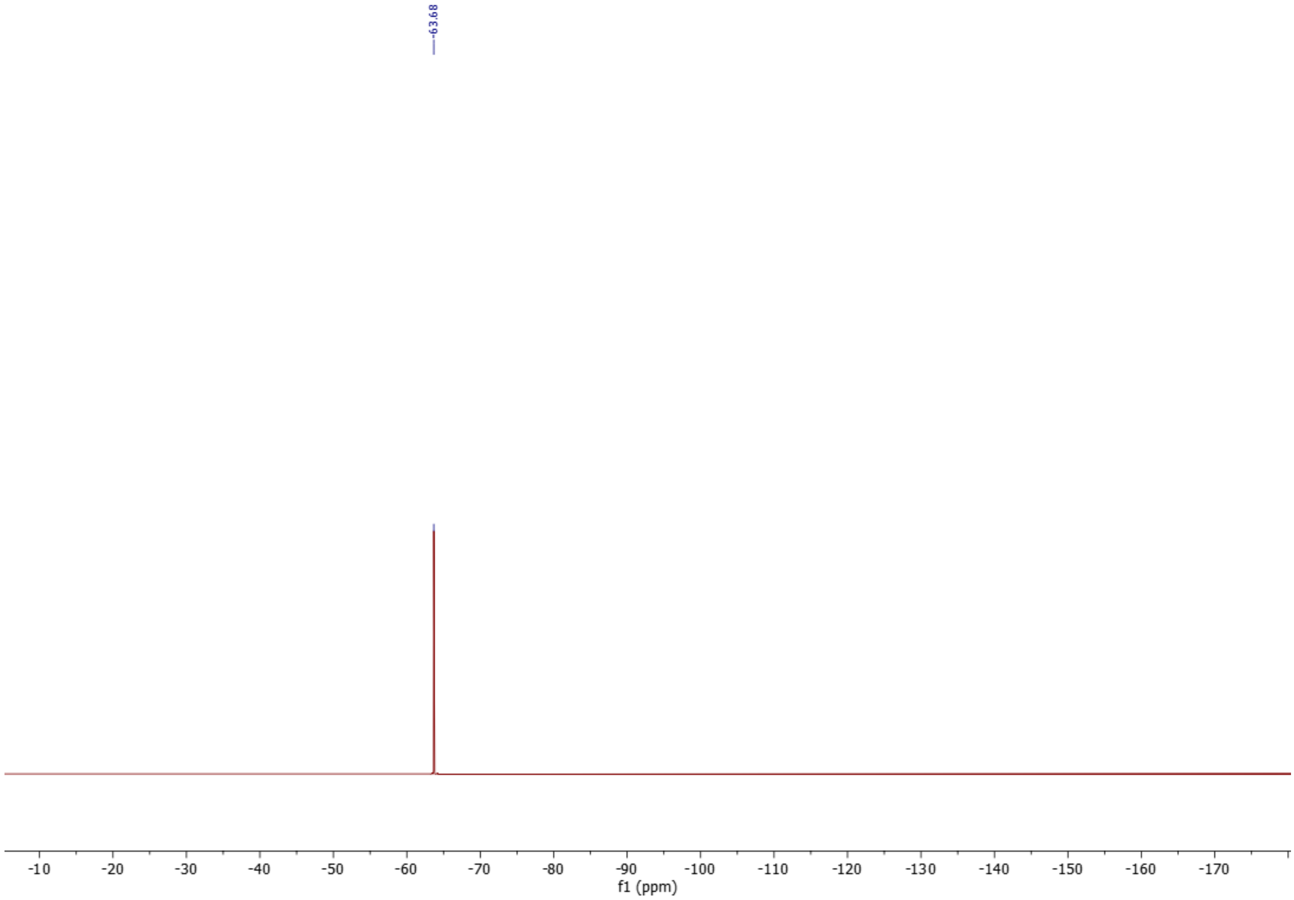

-63.68
-10
-20
-30
-40
-50
-60
-70
-80
-90
-100
-110
-120
-130
-140
-150
-160
-170
f1 (ppm)

trans-2-(4-nitrophenyl)-1-(thiazol-2-yl)cyclopropane-1-carbonitrile 5k

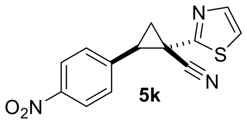

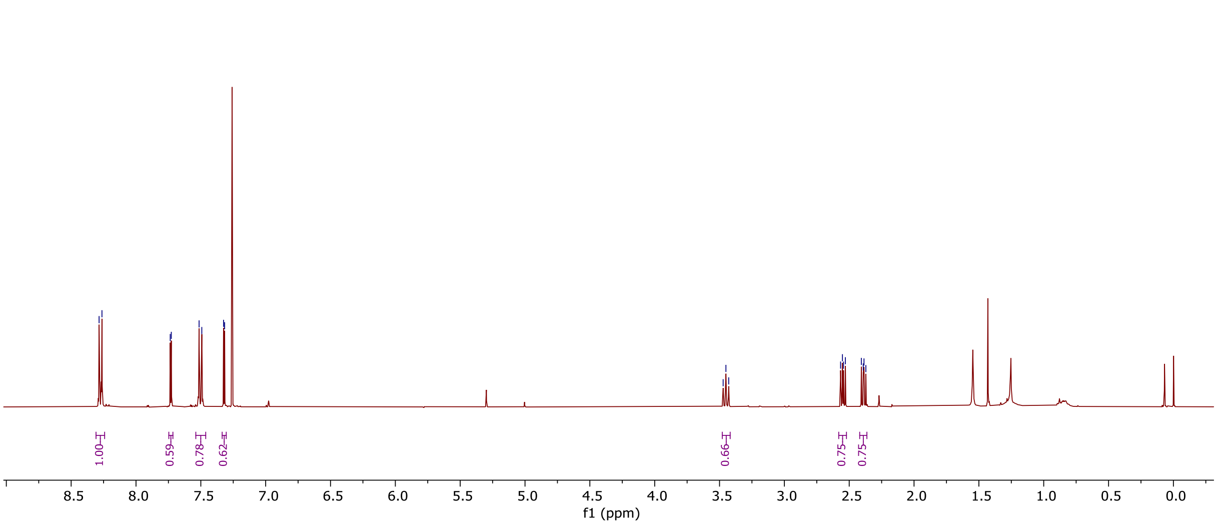

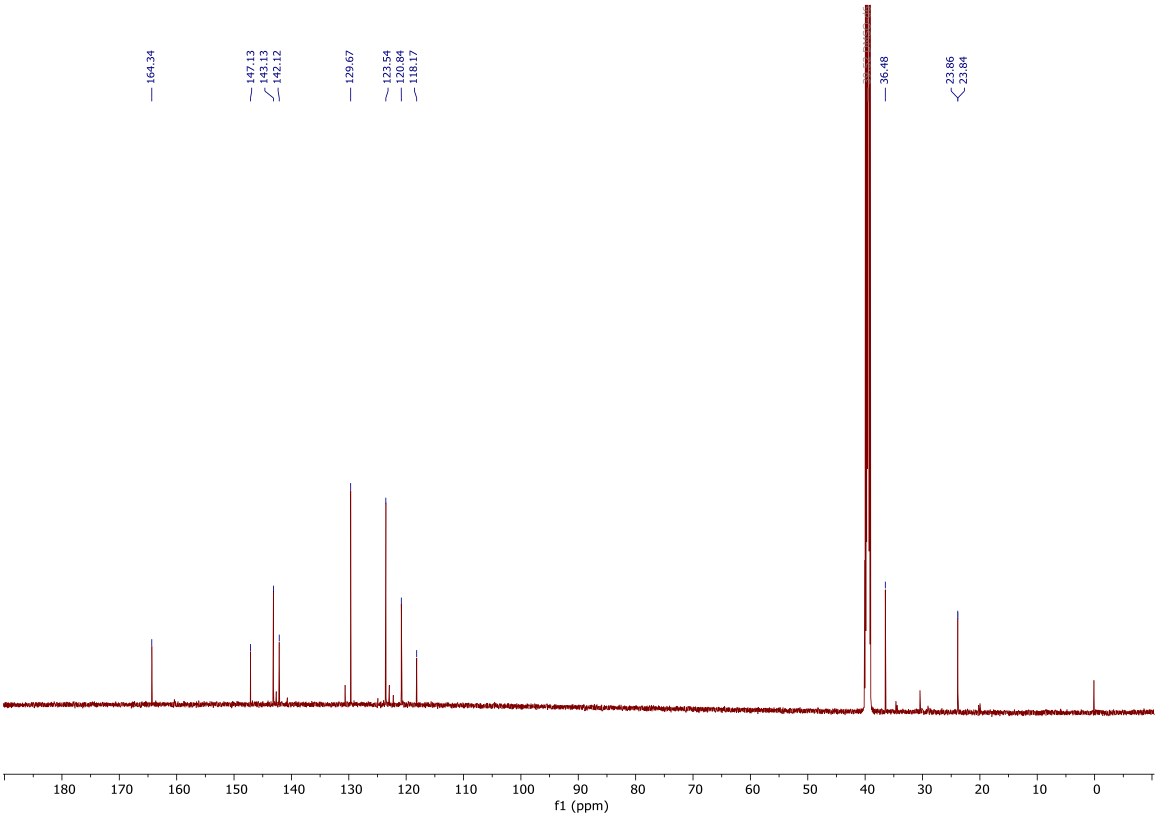

***trans*-2-(3-nitrophenyl)-1-(thiazol-2-yl)cyclopropane-1-carbonitrile 5l**

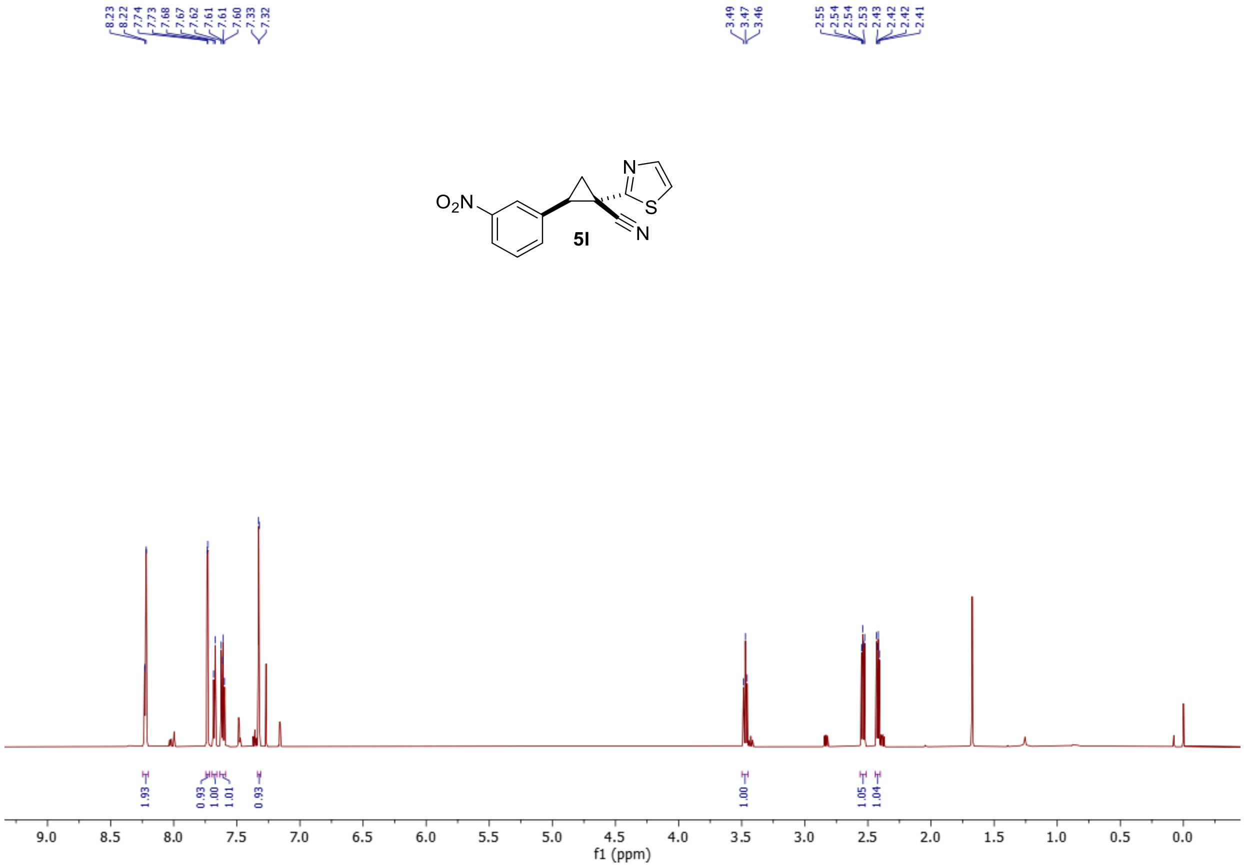

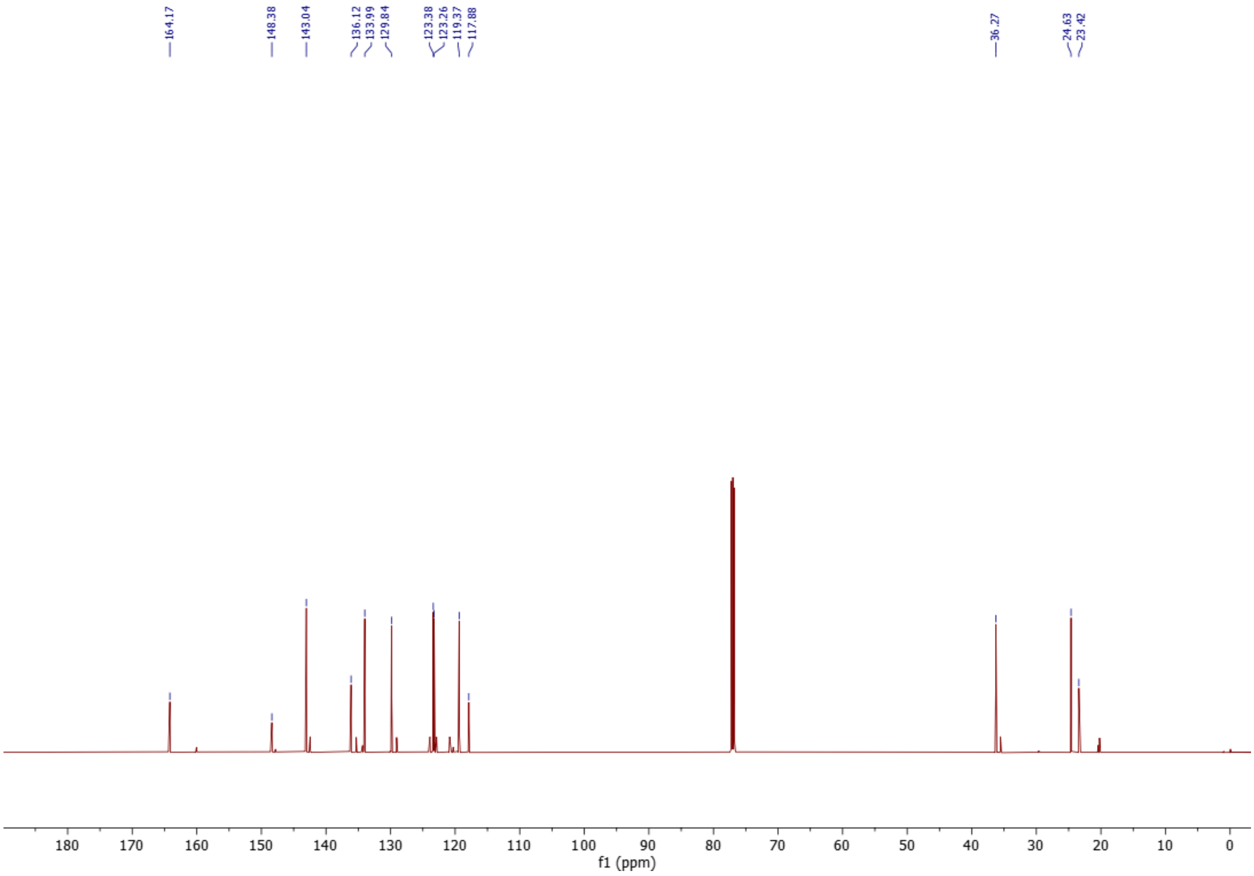

trans-2-(4-methoxyphenyl)-1-(thiazol-2-yl)cyclopropane-1-carbonitrile 5m

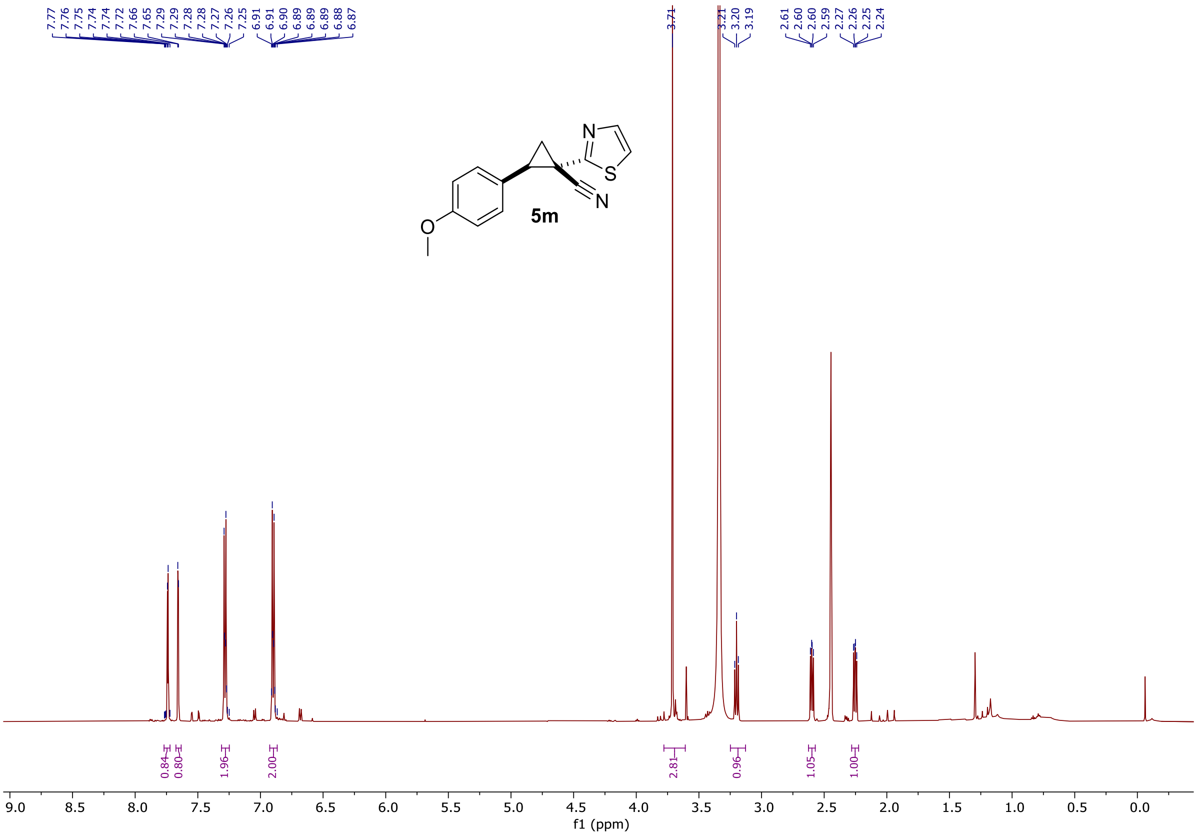

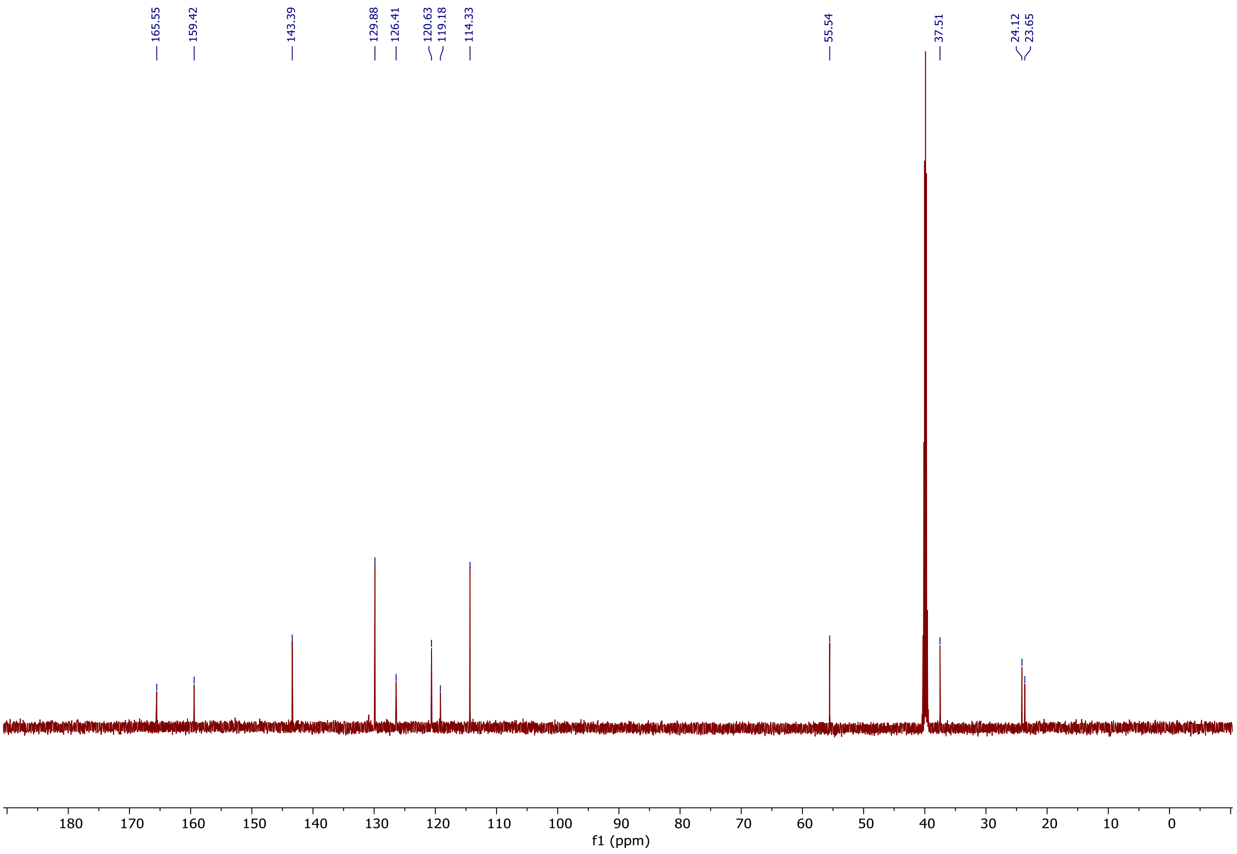

trans-2-(2-methoxyphenyl)-1-(thiazol-2-yl)cyclopropane-1-carbonitrile 5n

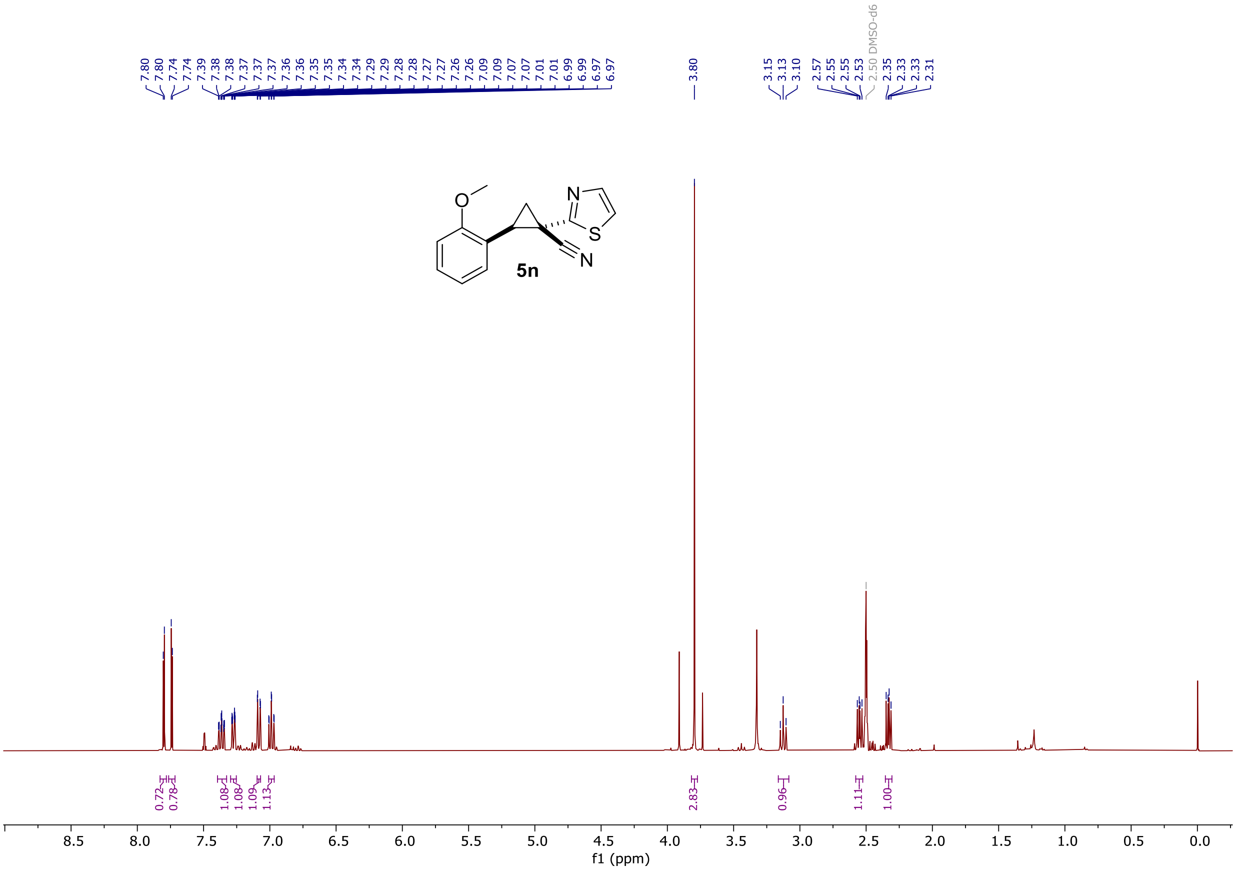

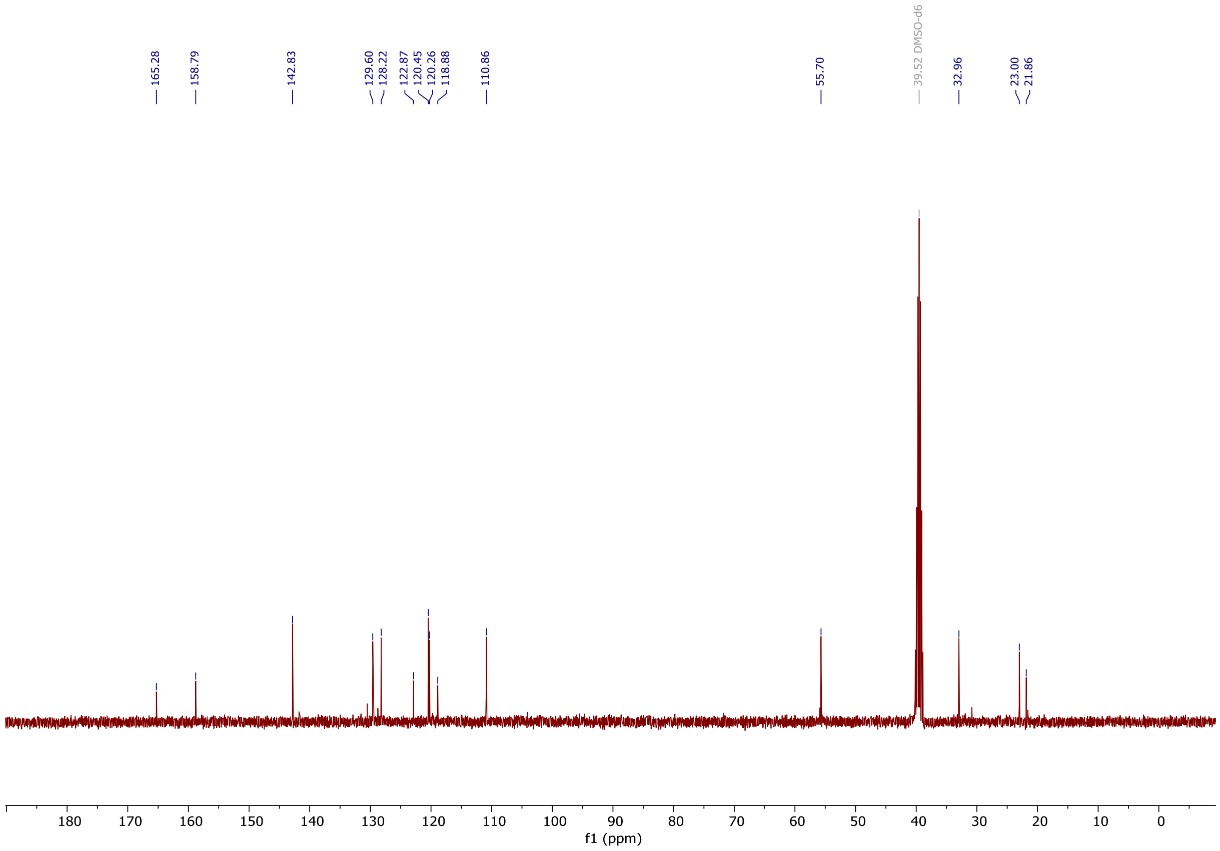

trans-2-(2-methylphenyl)-1-(thiazol-2-yl)cyclopropane-1-carbonitrile 5o

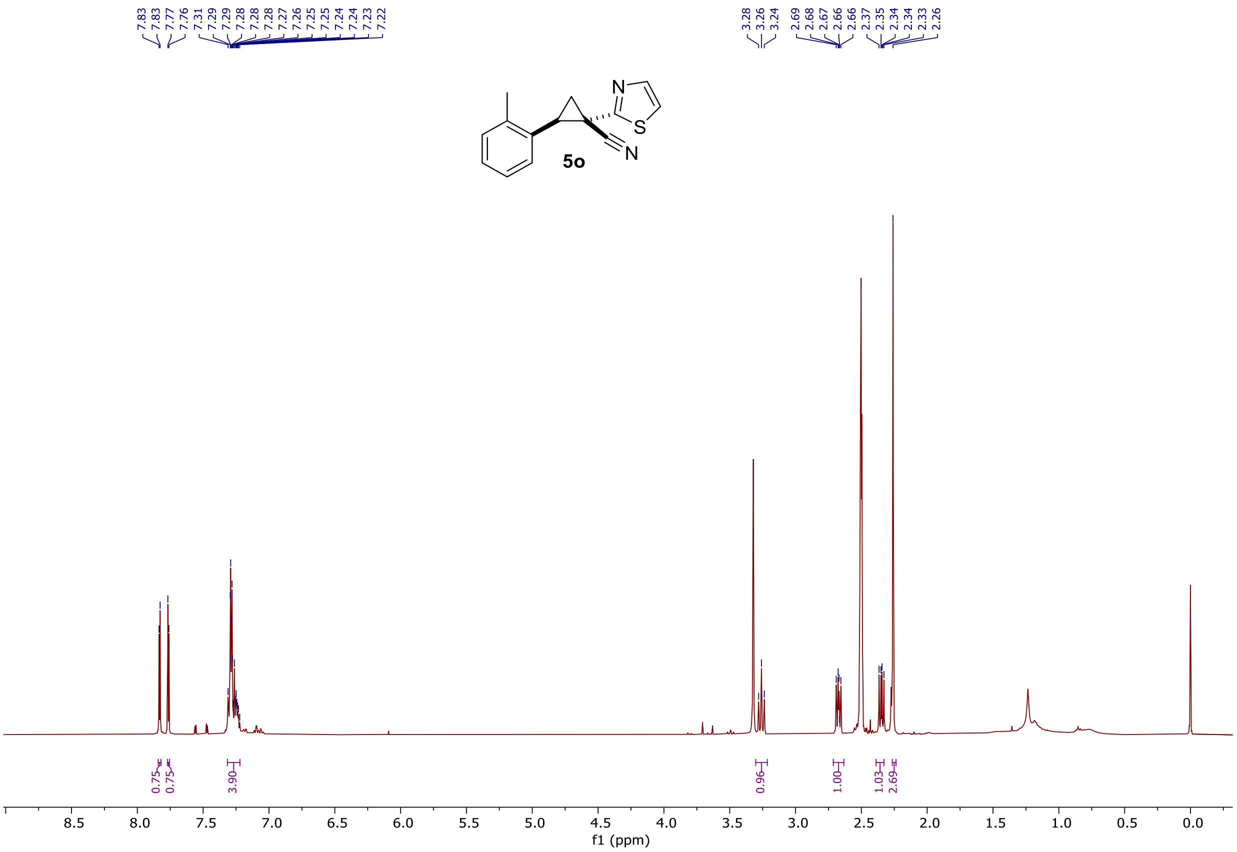

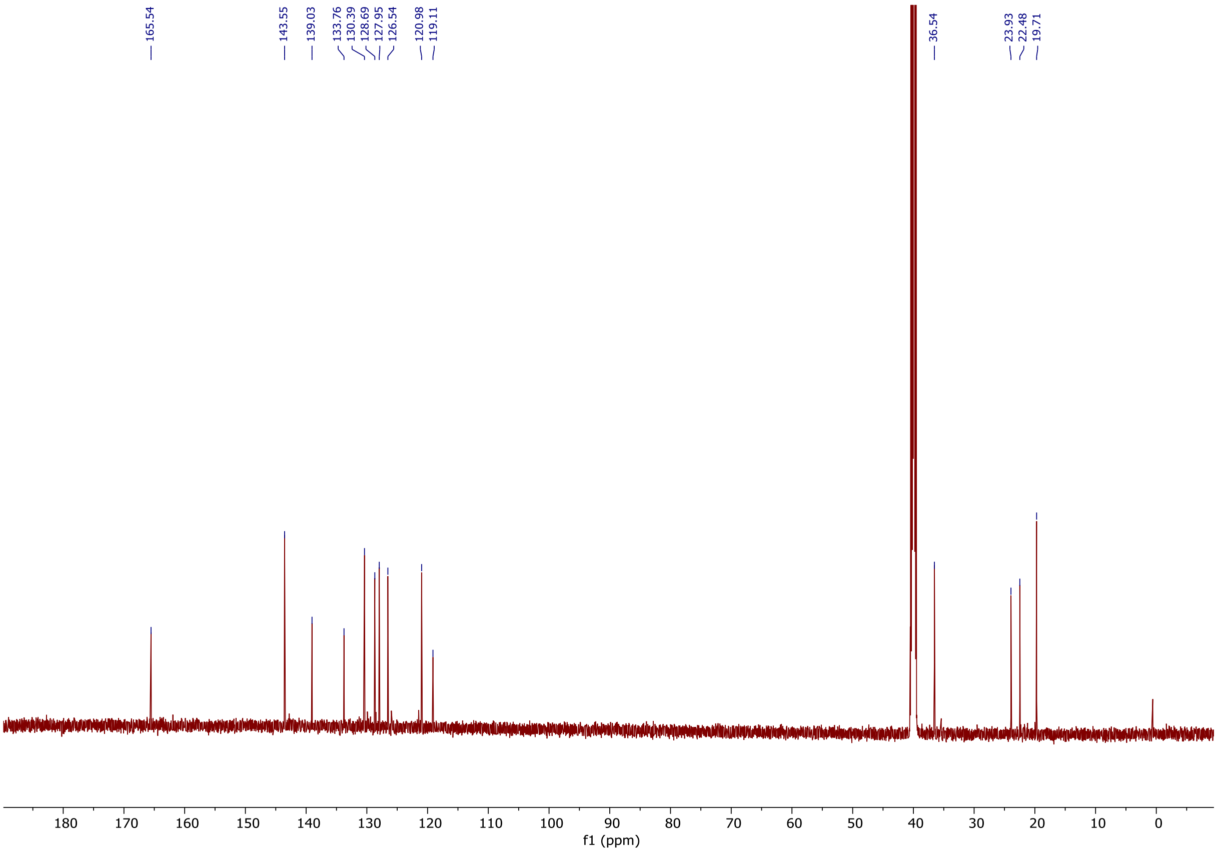

trans-1-(thiazol-2-yl)-2-(thiophen-2-yl)cyclopropane-1-carbonitrile 5p

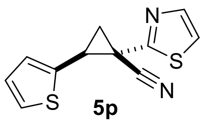

5p

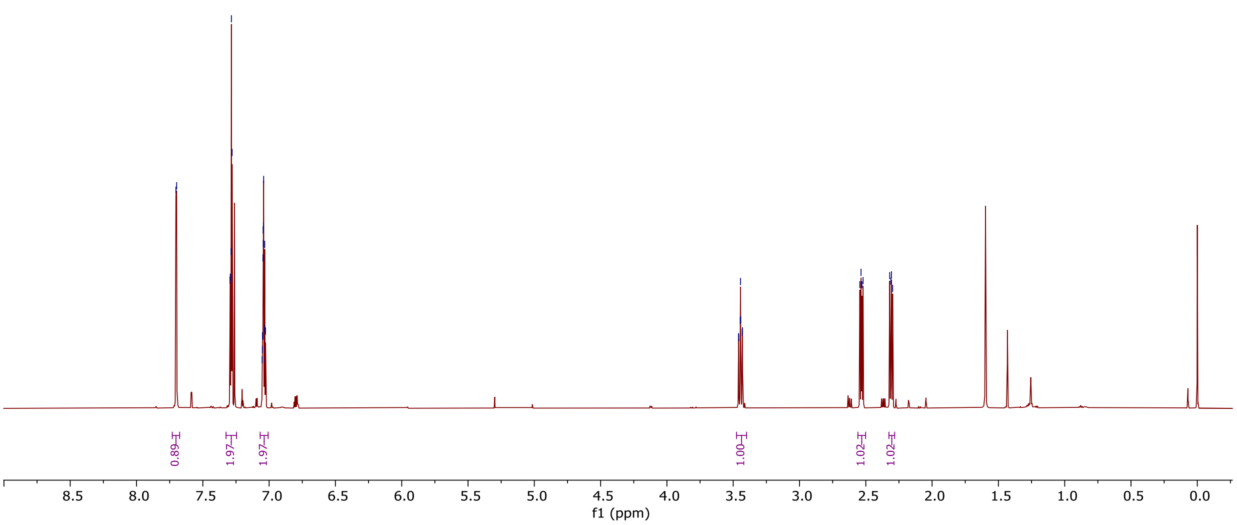

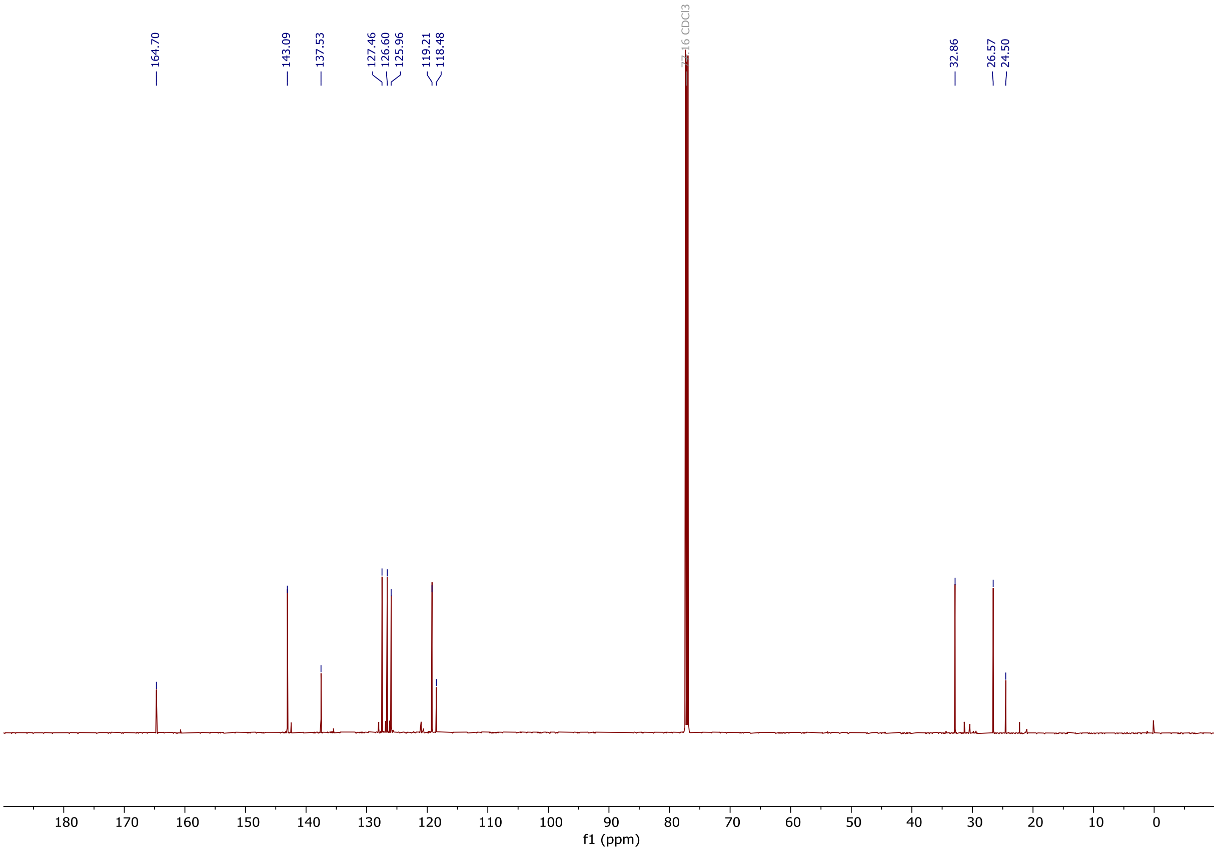

trans-2-(naphth-2-yl)-1-(thiazol-2-yl)cyclopropane-1-carbonitrile 5r

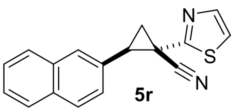

5r

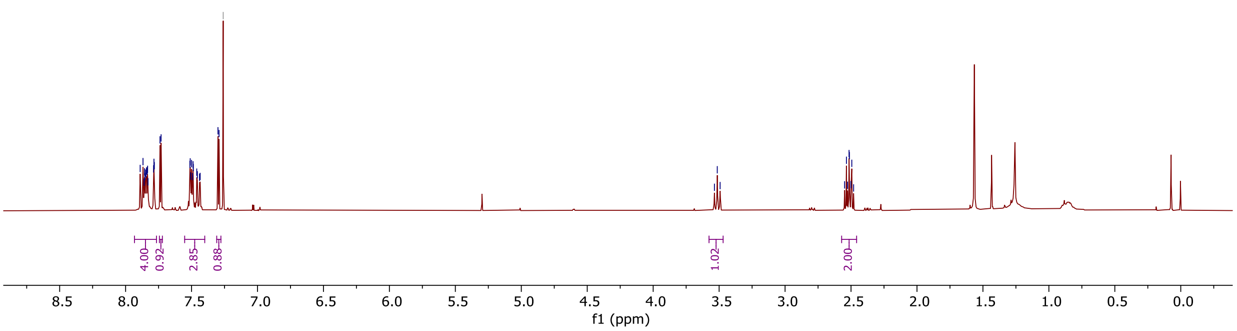

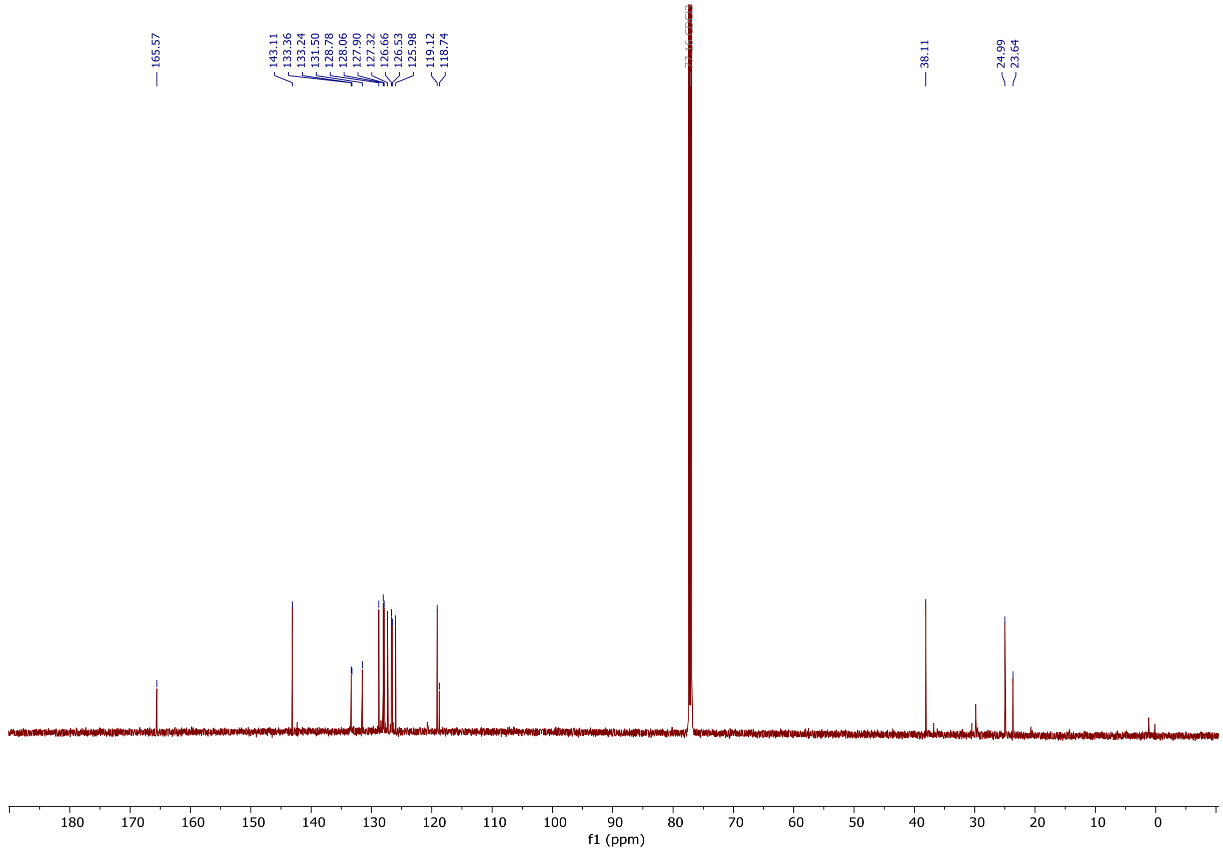

trans-2-(naphth-1-yl)-1-(thiazol-2-yl)cyclopropane-1-carbonitrile 5s

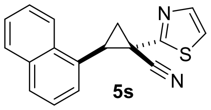

5s

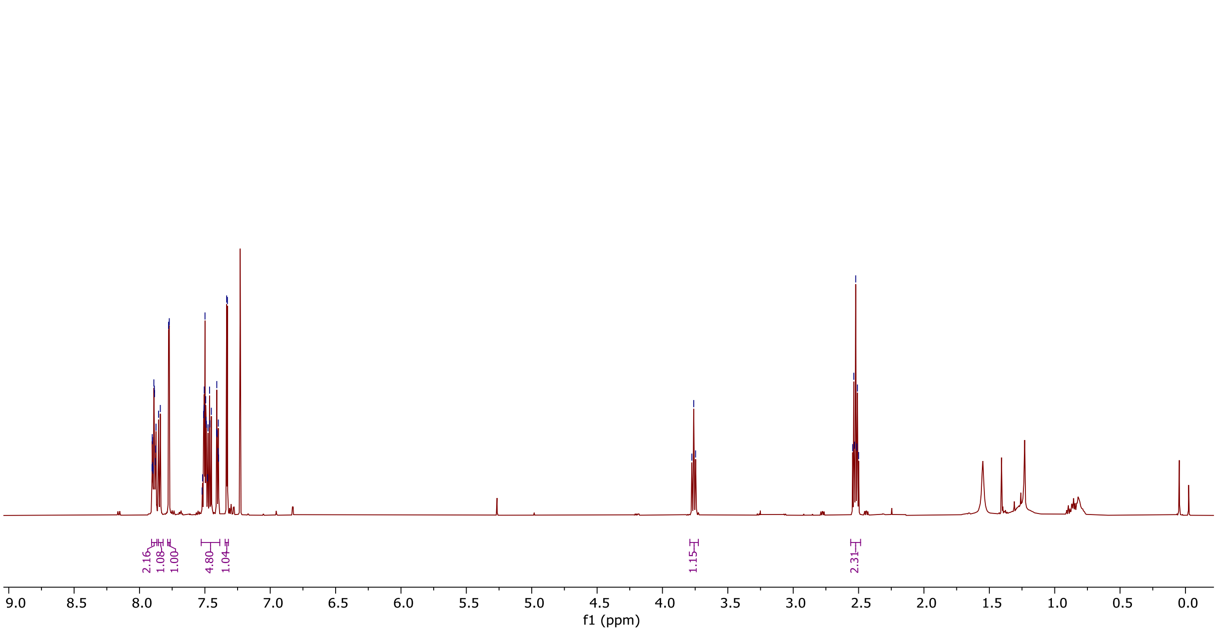

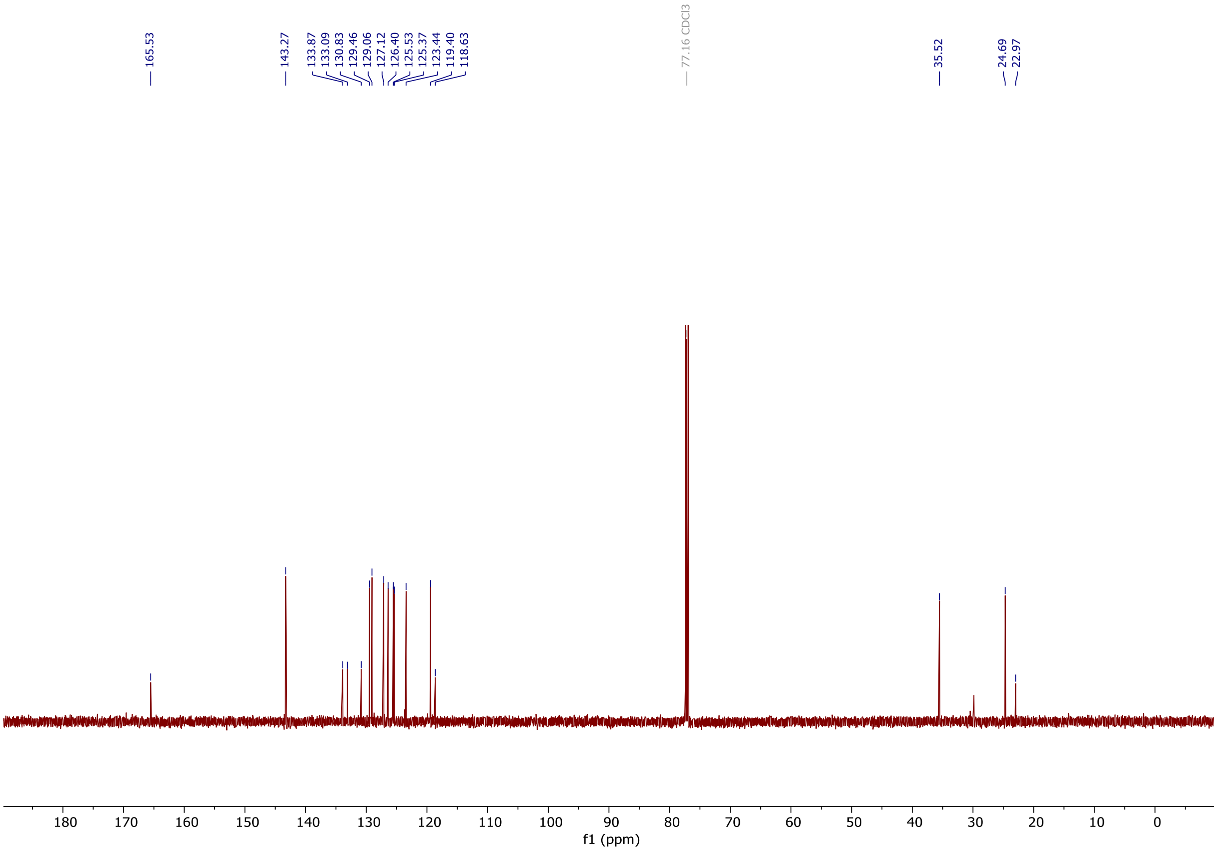

trans-2-phenyl-1-(thiazol-2-yl)cyclopropyl)methanamine 7a

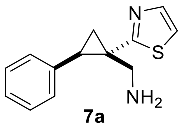

7a

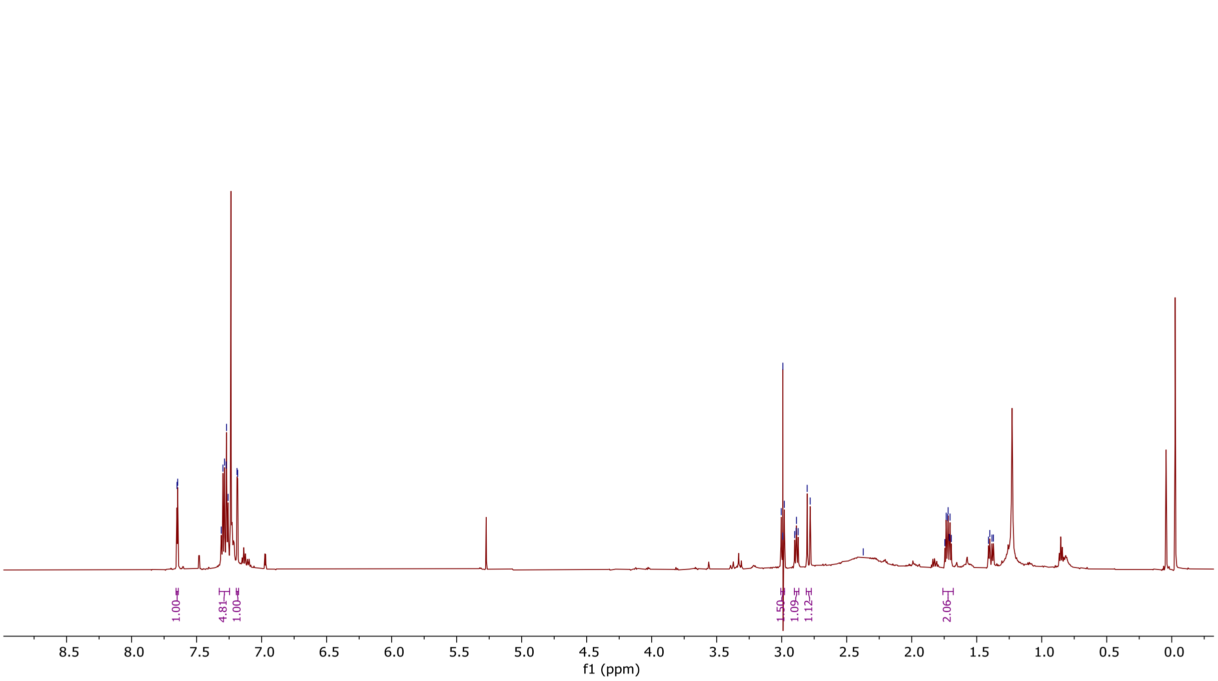

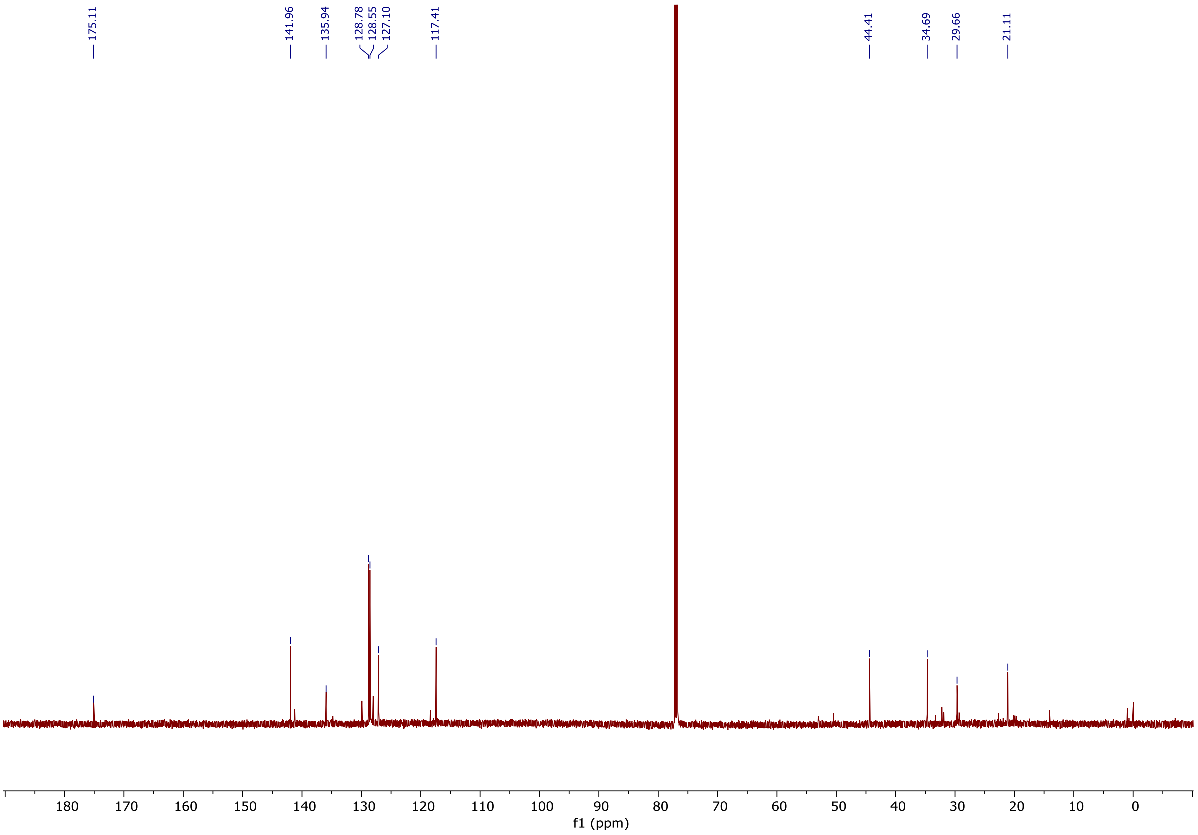

2,4-dichloro-4-phenyl-2-(thiazol-2-yl)butanenitrile 8a (mixture of diastereoisomers dr 1:1)

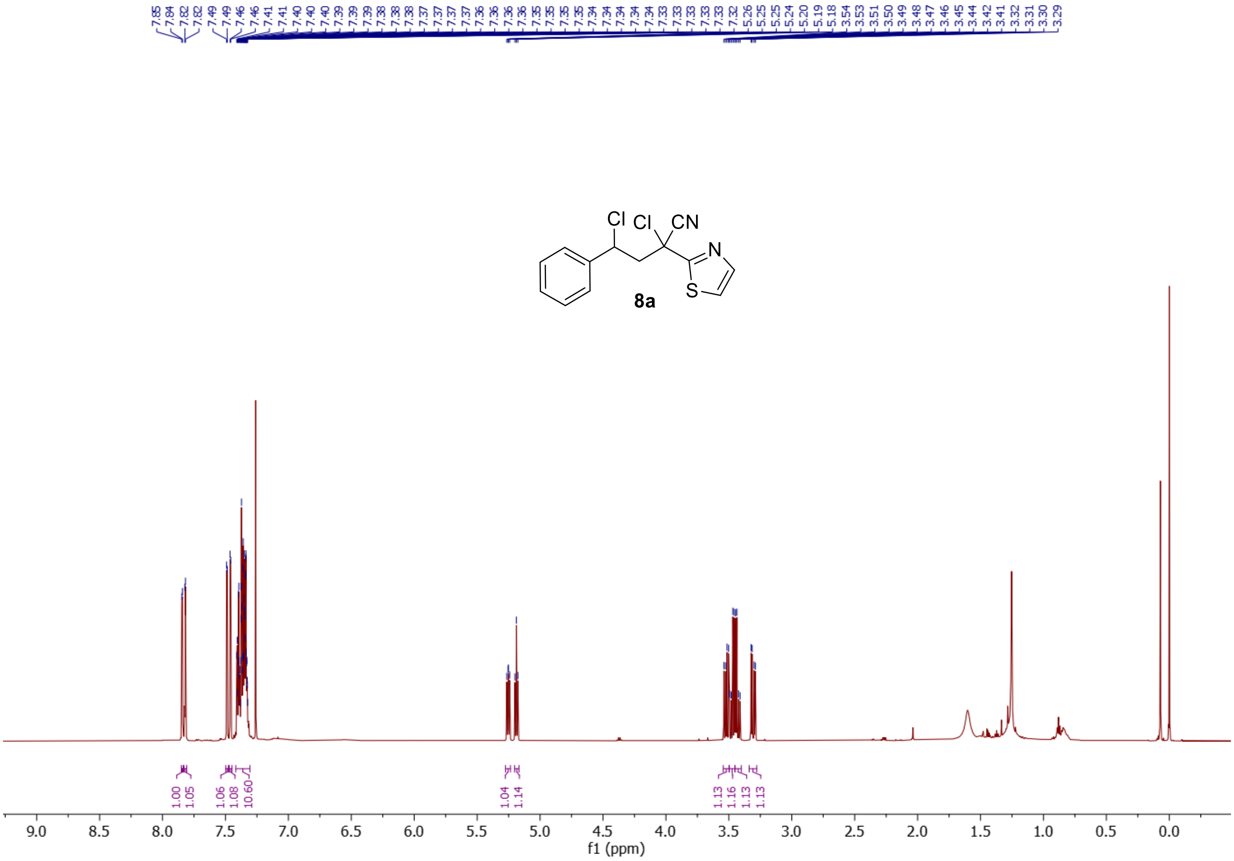

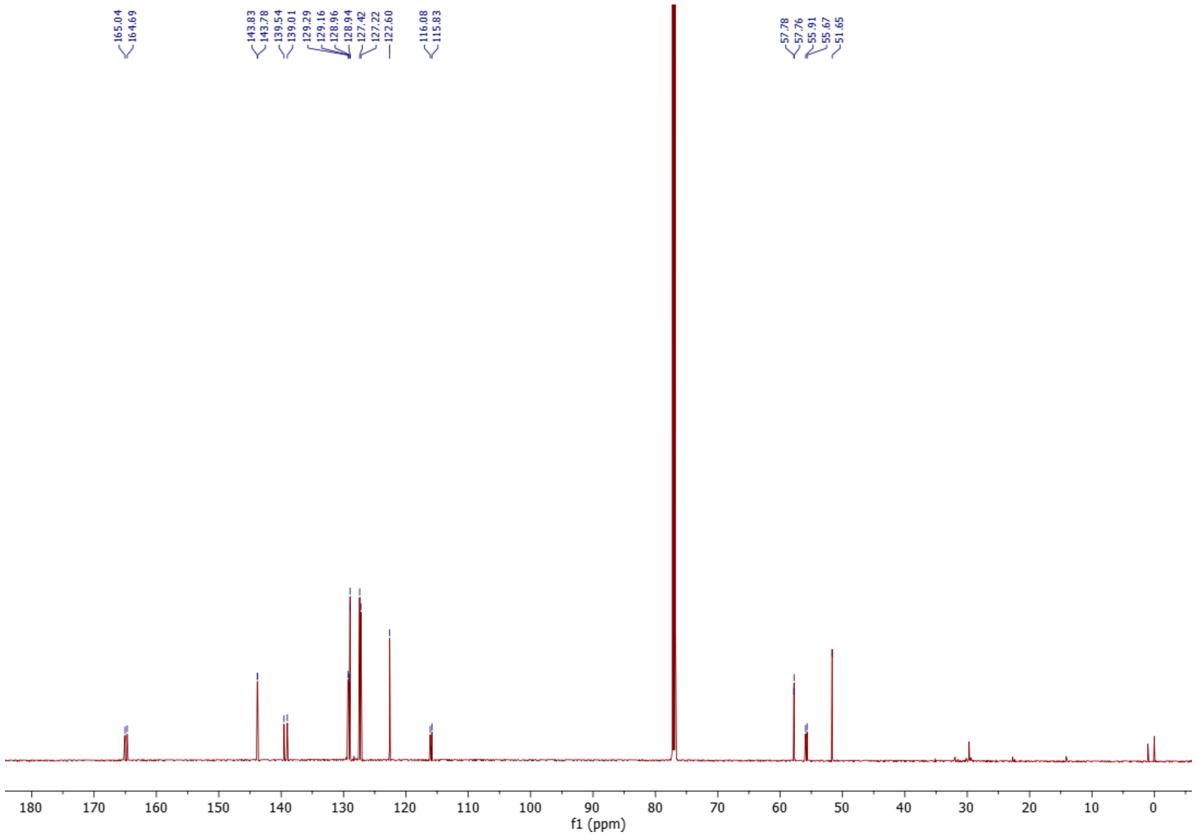

2,4-dichloro-4-(4-cyanophenyl)-2-(thiazol-2-yl)butanenitrile 8b (mixture of diastereo-isomers dr 1:1.3)

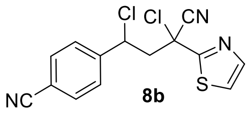

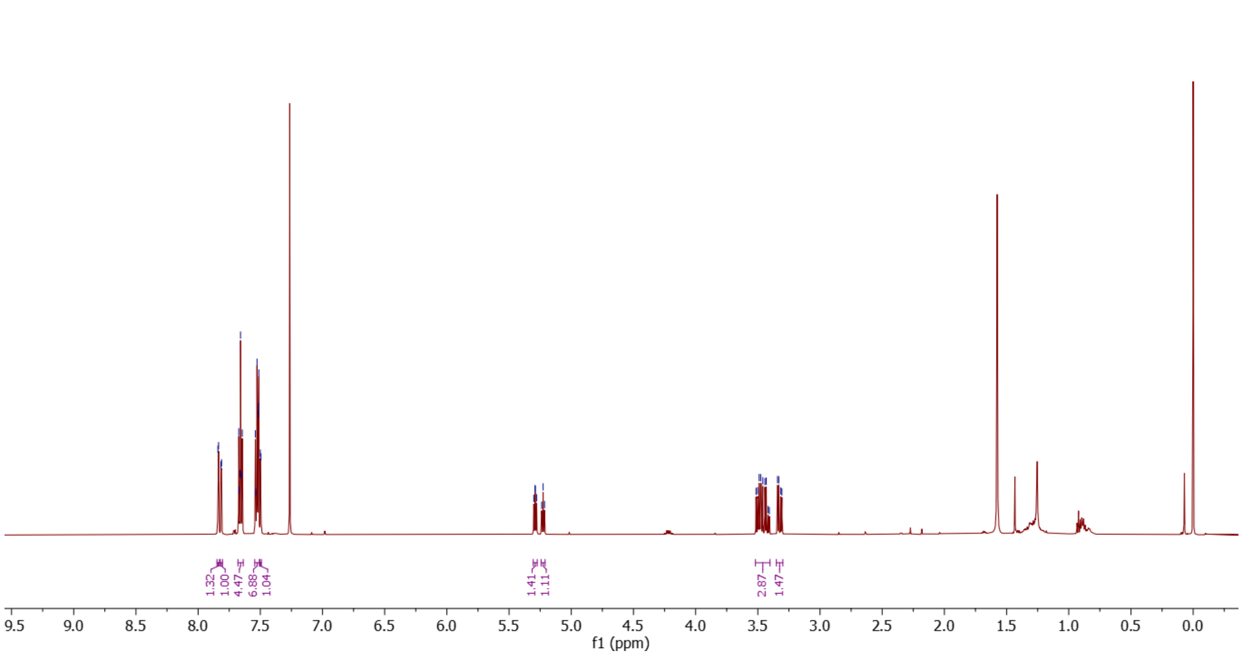

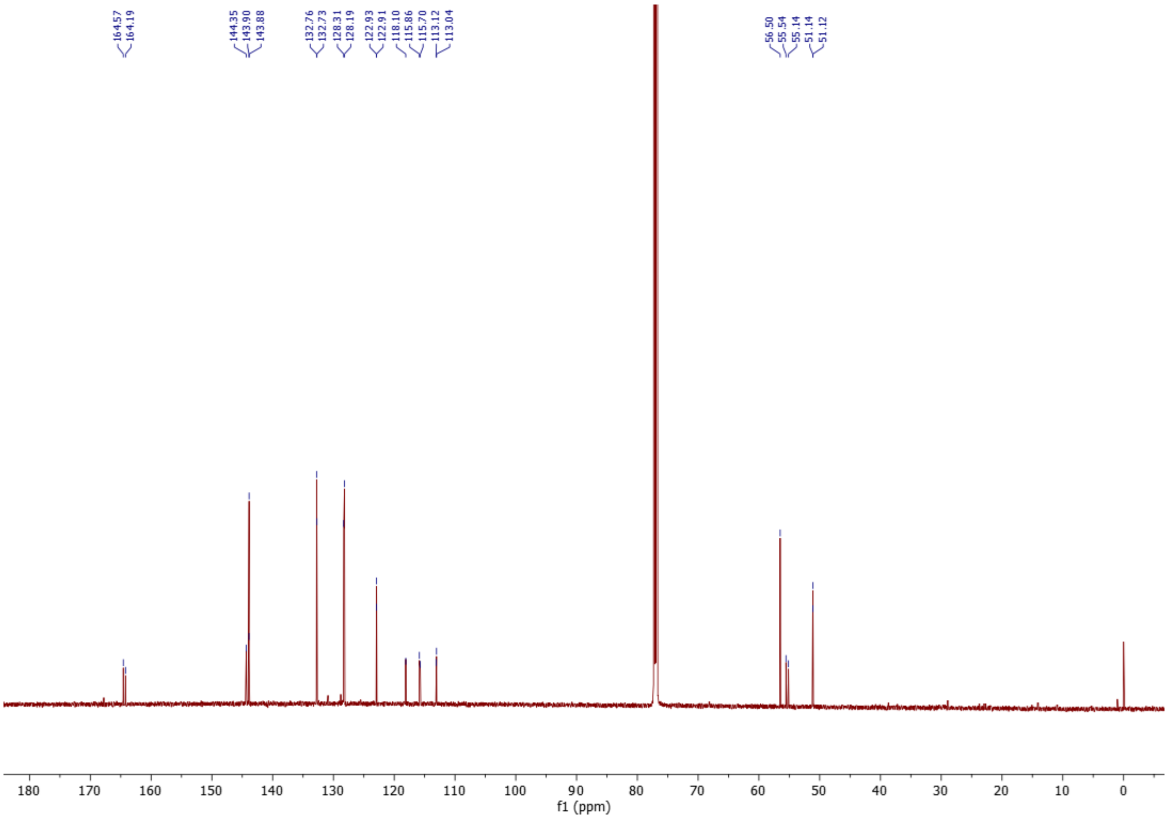

2,4-dichloro-4-(4-nitrophenyl)-2-(thiazol-2-yl)butanenitrile 8c (mixture of diastereo-isomers dr 1:1.4)

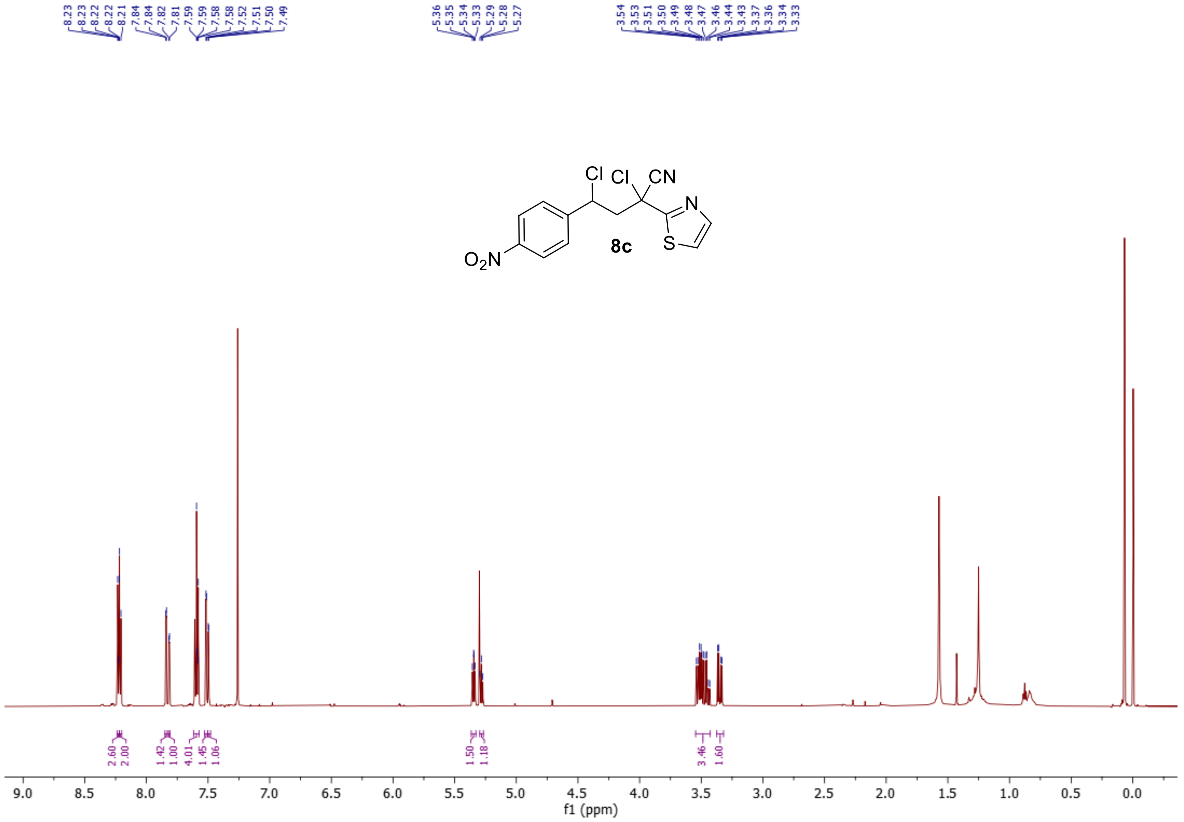

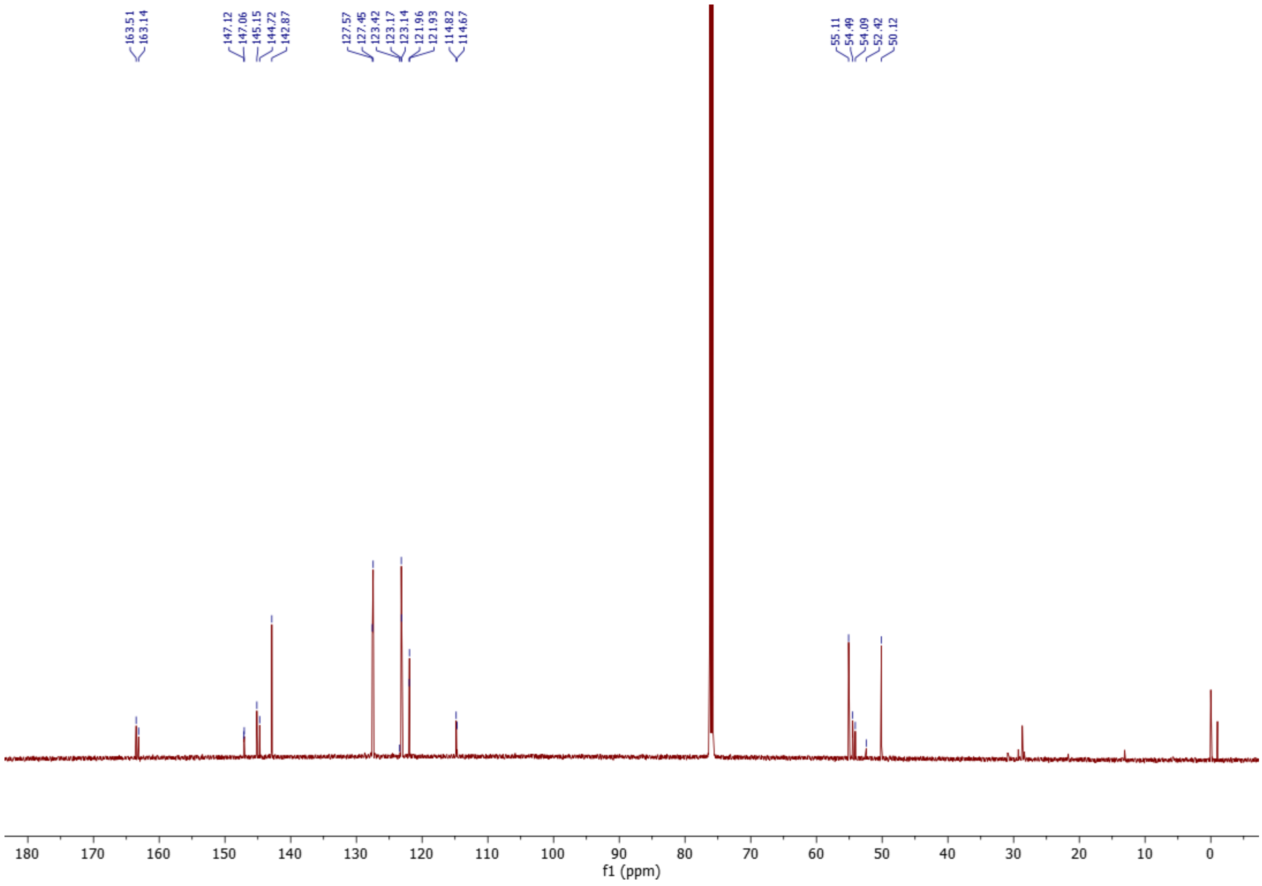

4-phenyl-2-(thiazol-2-yl)butanenitrile 9a

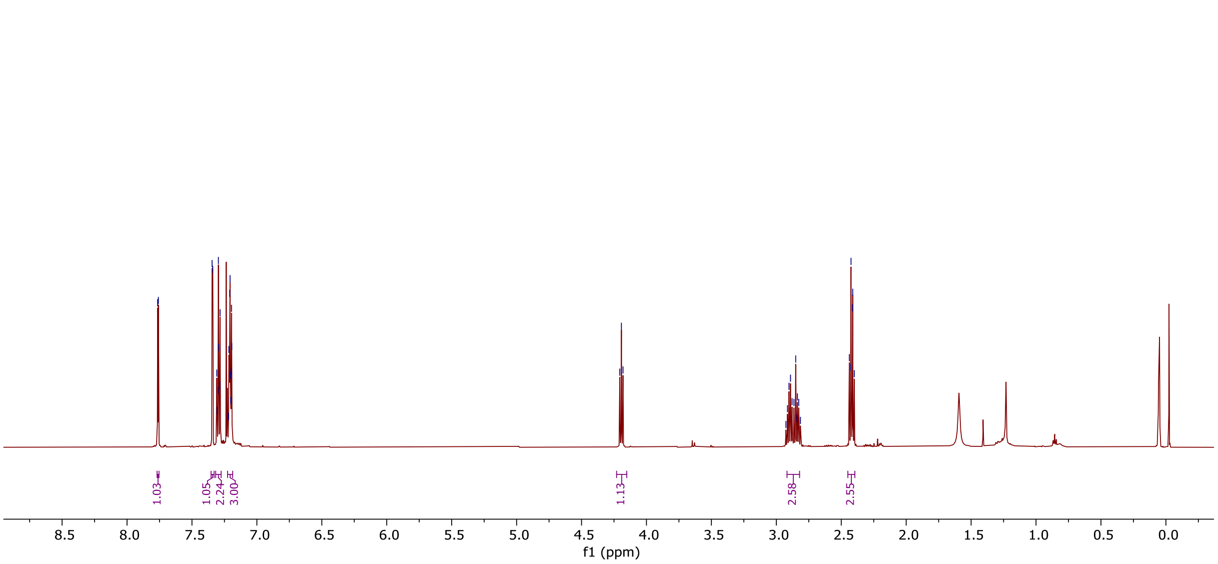

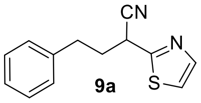

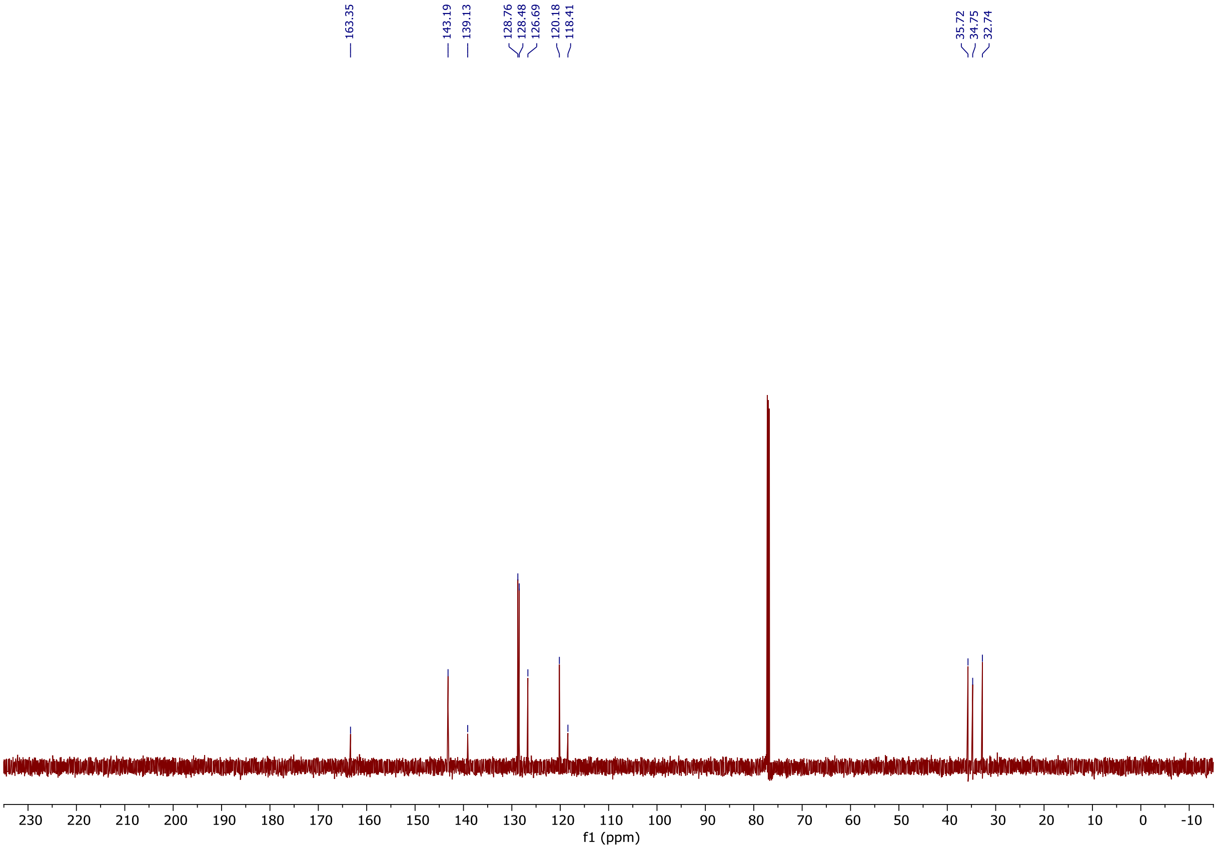

4-(2-chlorophenyl)-2-(thiazol-2-yl)butanenitrile 9b

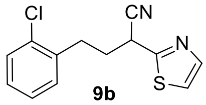

9b

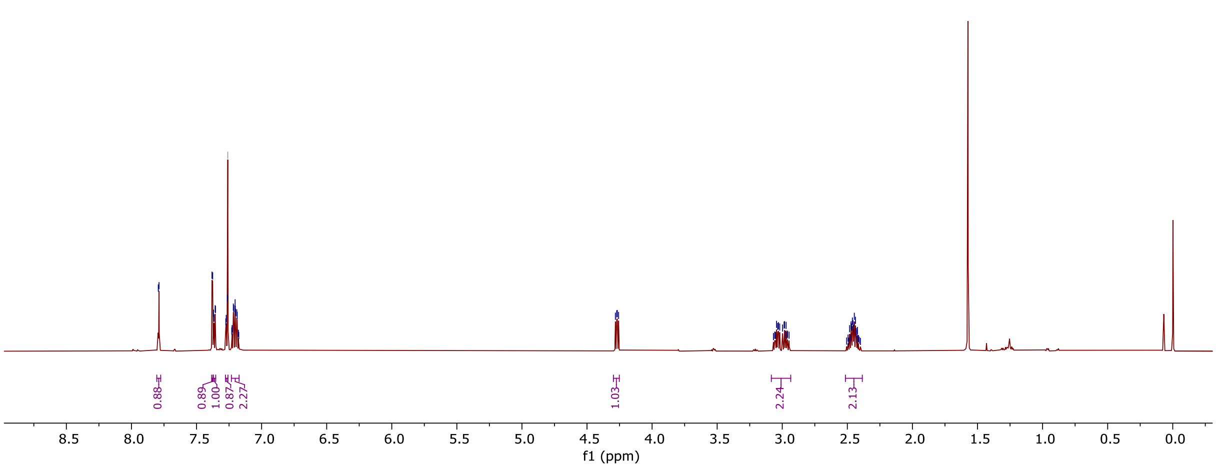

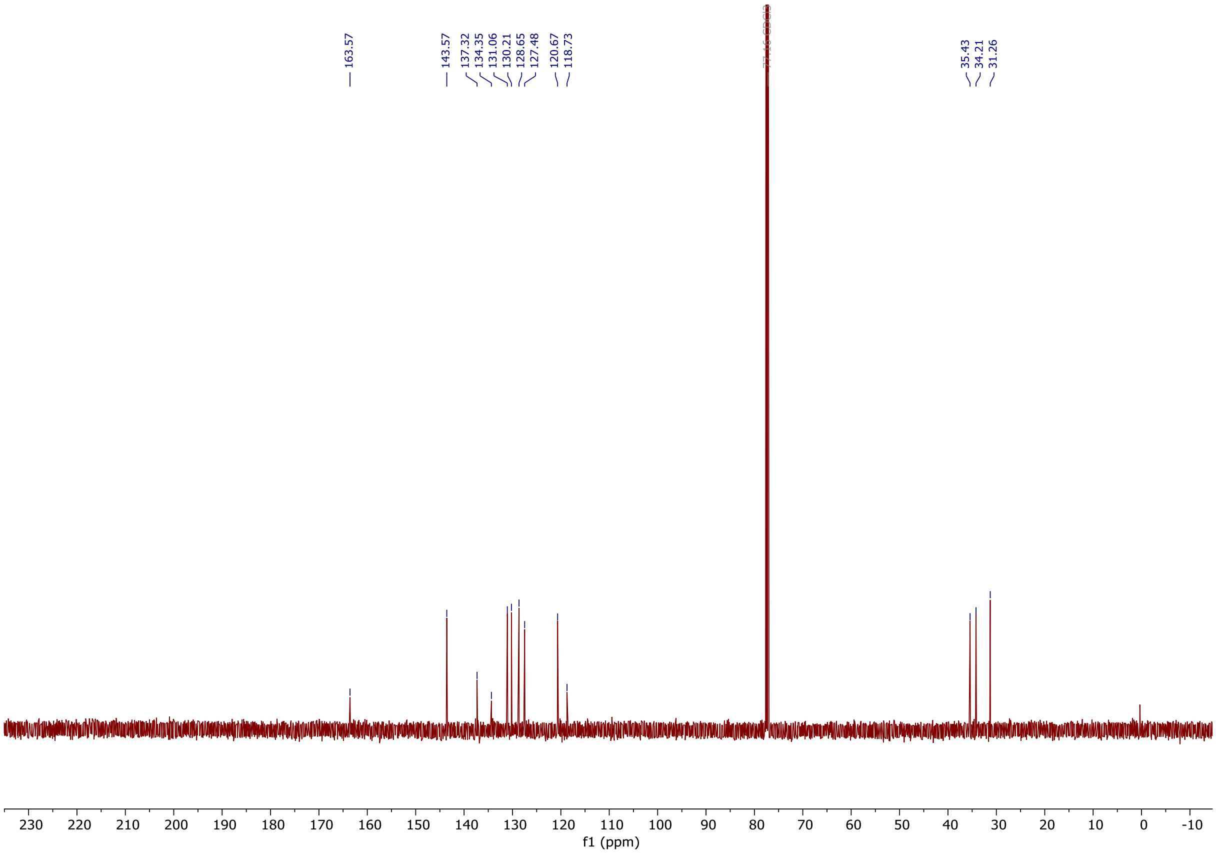

2-(thiazol-2-yl)-4-(4-(trifluoromethyl)phenyl)butanenitrile 9c

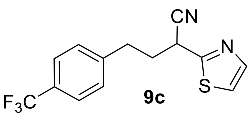

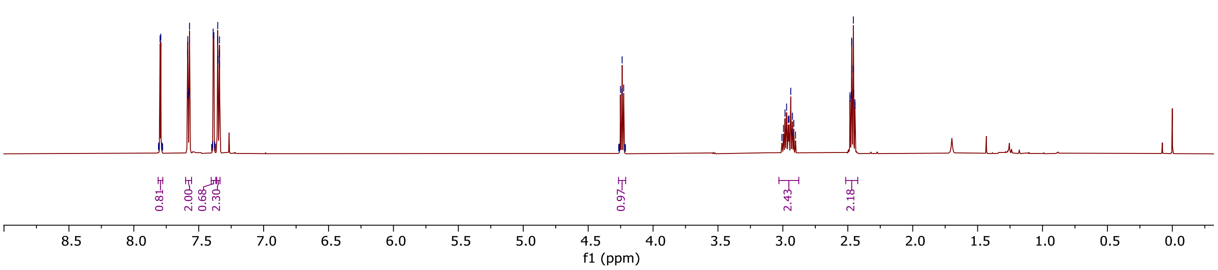

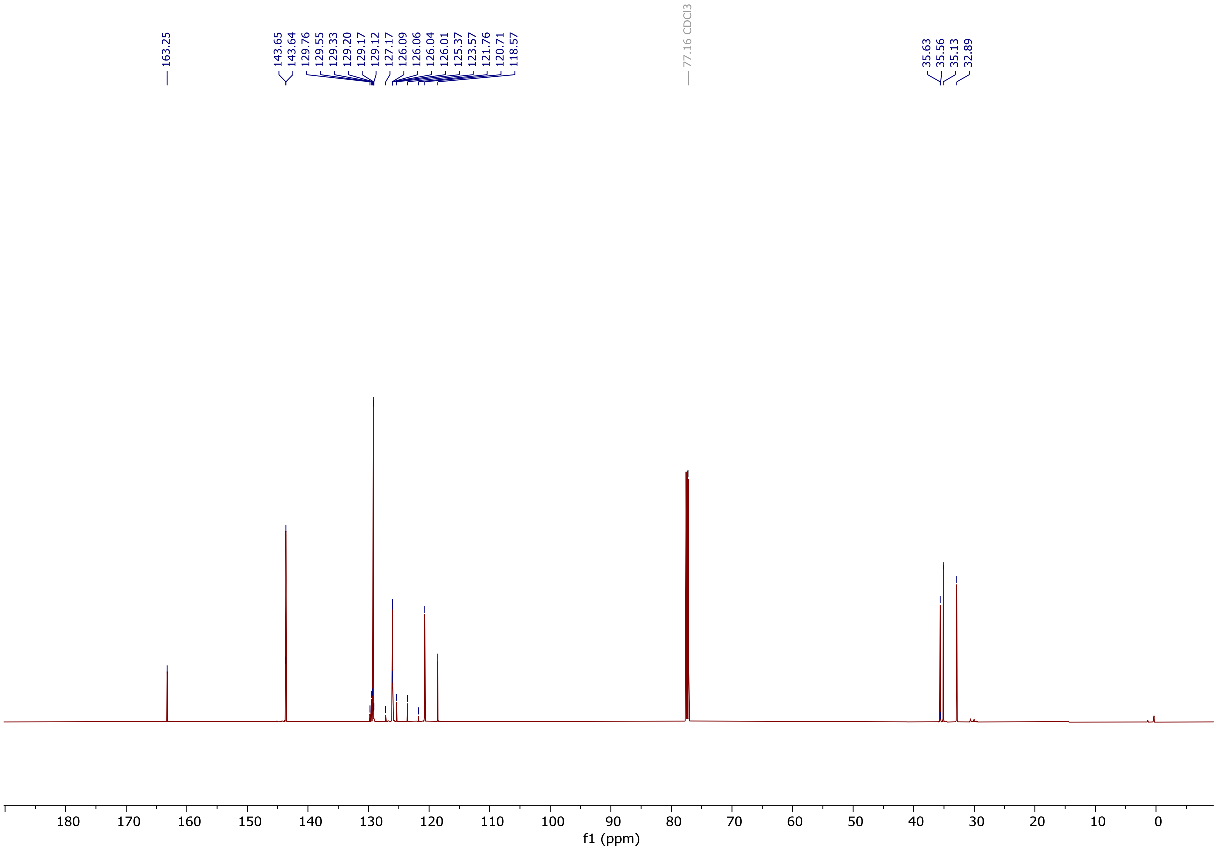

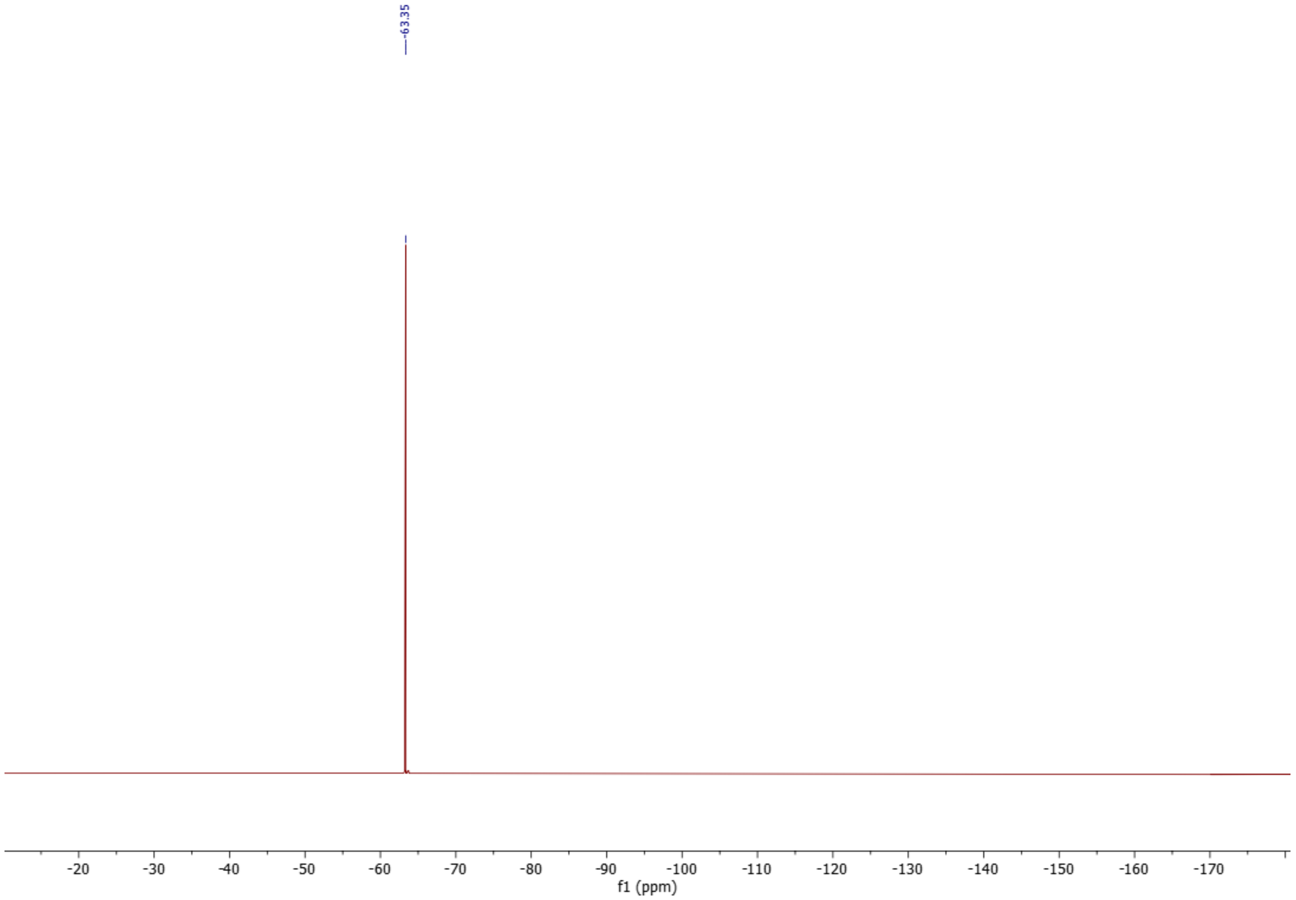

-63.35
-20
-30
-40
-50
-60
-70
-80
-90
-100
-110
-120
-130
-140
-150
-160
-170
f1 (ppm)

4-phenyl-2-(thiazol-2-yl)-4-(2,4,6-trimethoxyphenyl)butanenitrile 10a (mixture of diastereo-isomers dr 1:1.5)

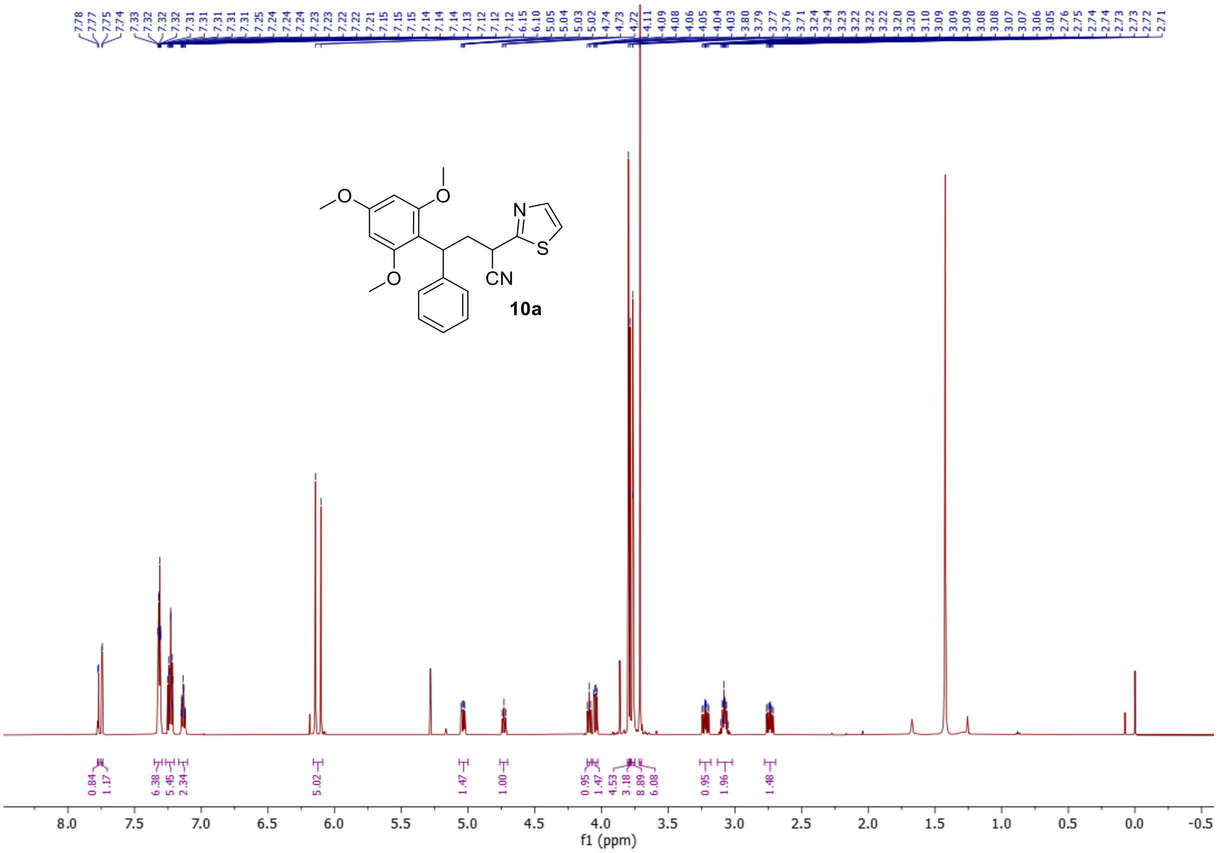

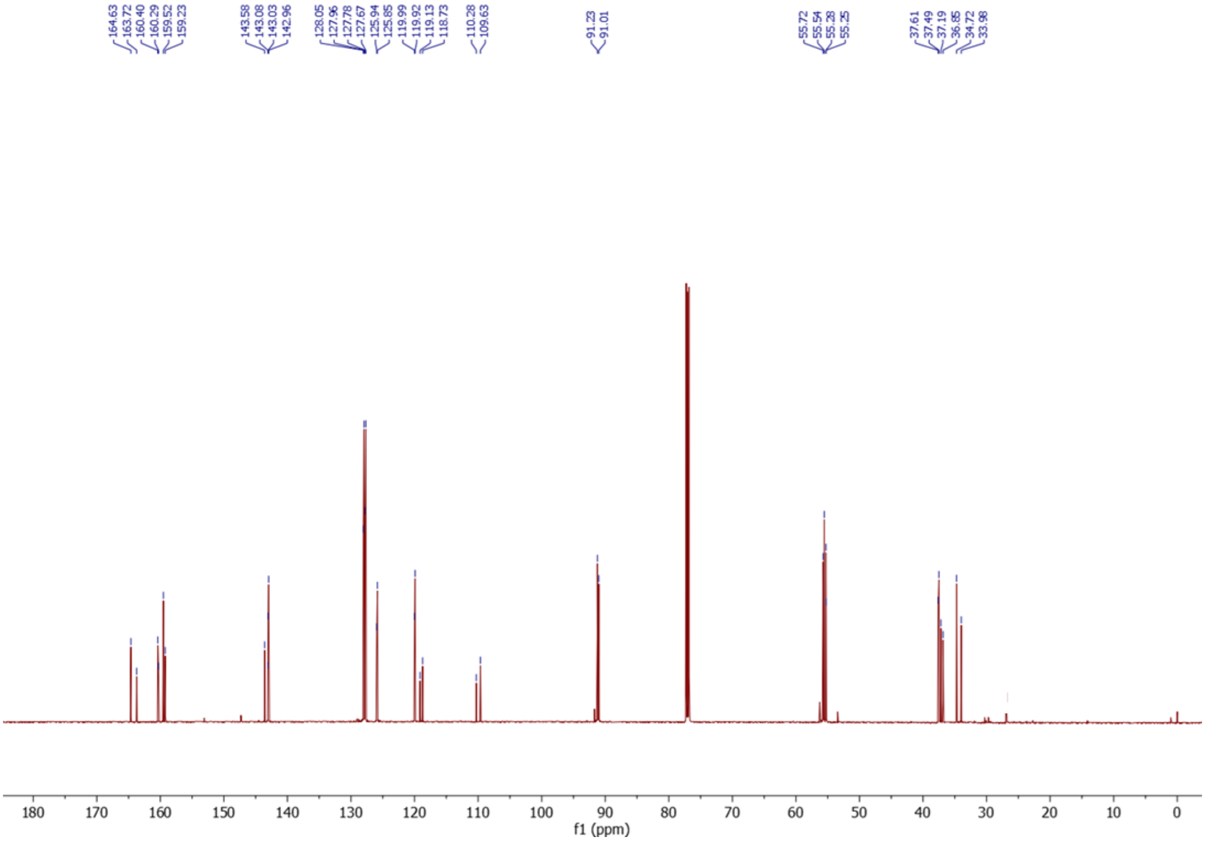

4-(2-chlorophenyl)-2-(thiazol-2-yl)-4-(2,4,6-trimethoxyphenyl)butanenitrile 10b (mixture of diastereoisomers dr 1:1.4)

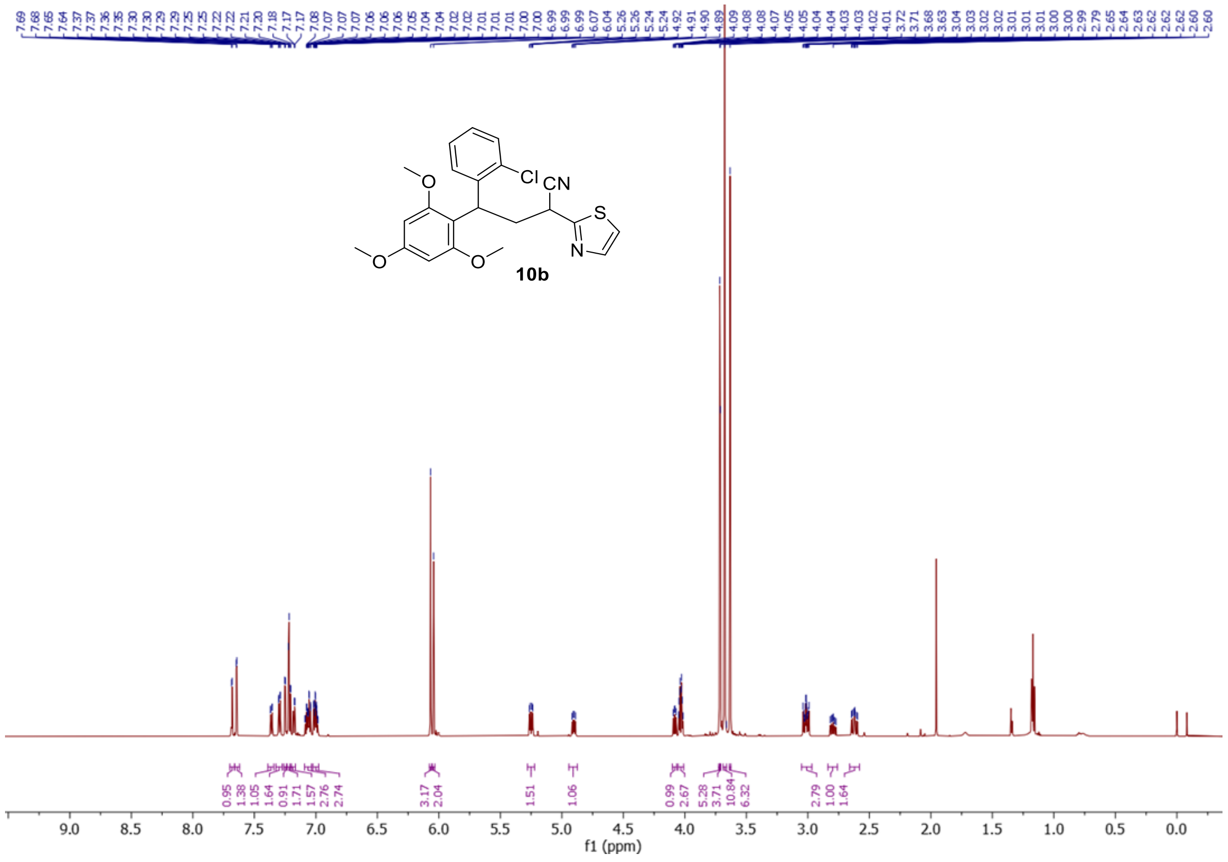

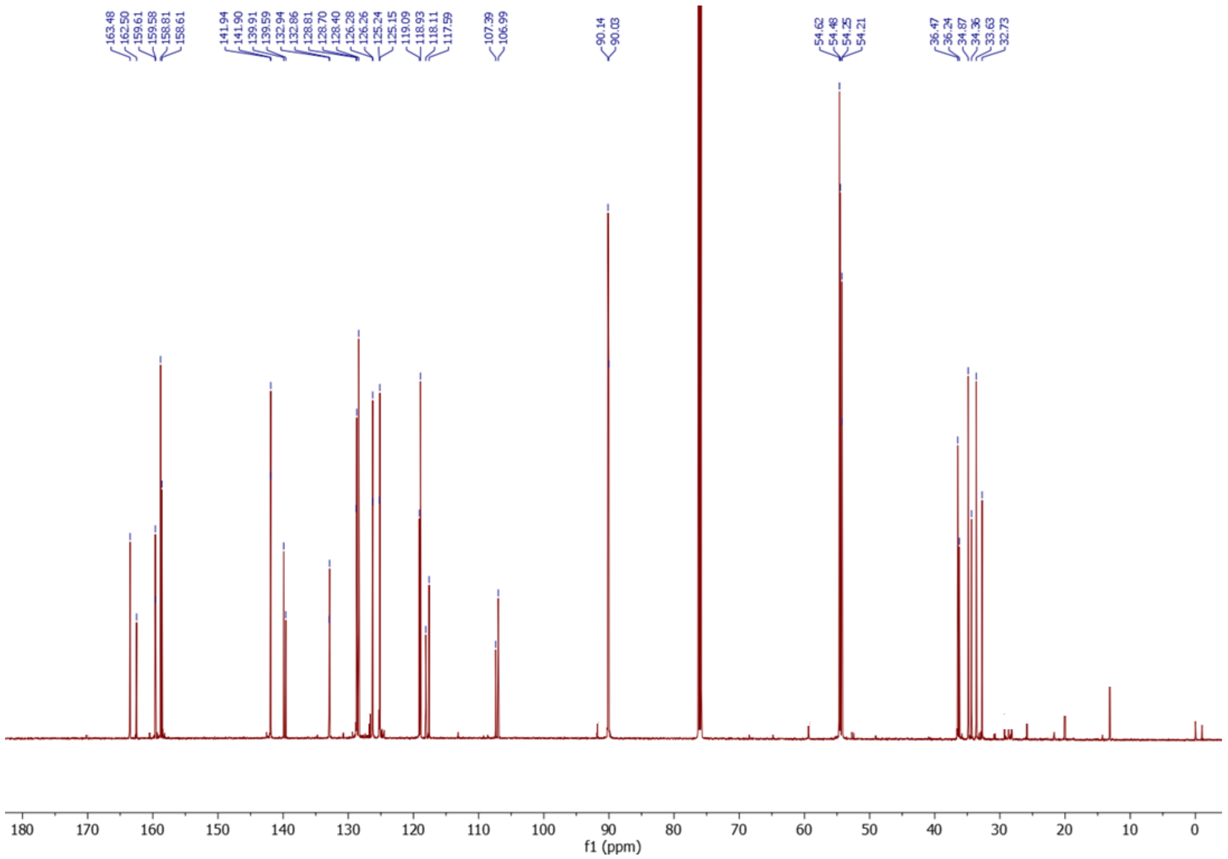

4-(3-cyano-3-(thiazol-2-yl)-1-(2,4,6-trimethoxyphenyl)propyl)benzonitrile 10c (mixture of diastereoisomers dr 1:1.4)

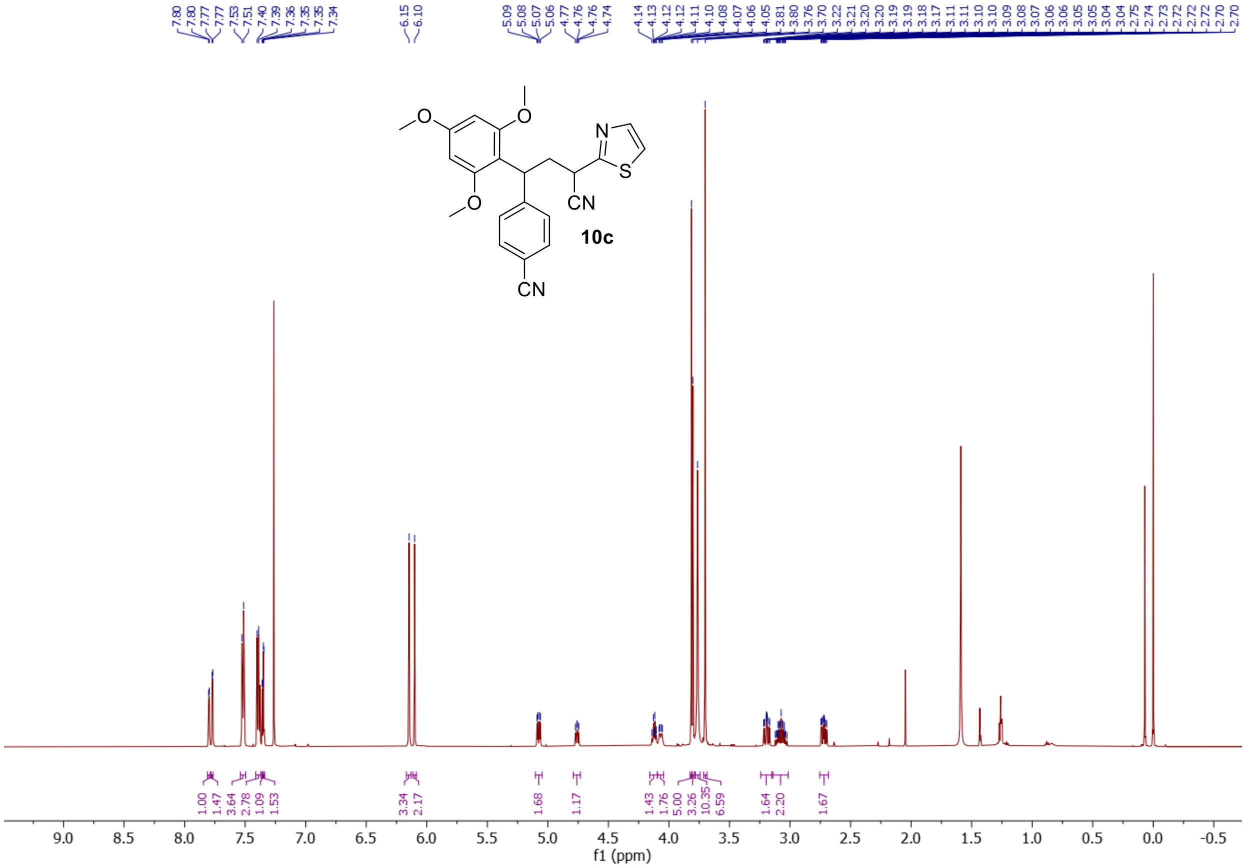

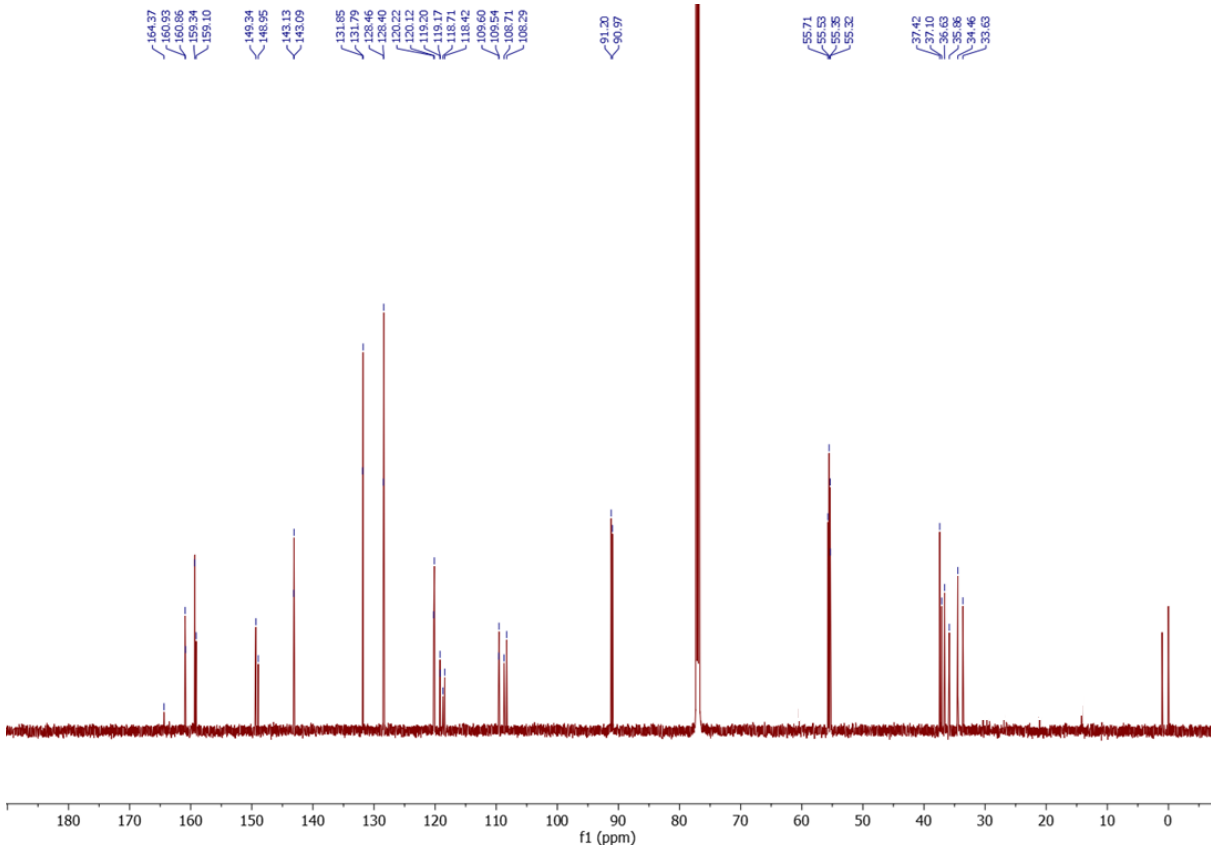

Geometries:

1+2m G=-1085.099201E_h
29
Coordinates from ORCA-job startingproto E -1085.265347698554

C	3.84395182918811	1.14069636816688	0.05744760167537
C	4.37035122558595	-0.14746783335172	0.04180051783012
C	3.51292092281379	-1.24235240056154	-0.00421714779062
C	2.13713467222713	-1.04955273600700	-0.03059418352897
C	1.60114899508152	0.24251609792096	-0.01680045011268
C	2.46854349244277	1.33708649370589	0.02489474315130
C	0.12499667141744	0.37455451356889	-0.04644606958885
C	-0.63646804819868	1.13650648718932	1.09539344486328
C	-0.60978543996597	1.62672745516443	-0.35296433355410
C	-1.83789663679321	0.51851066267903	1.57541588677575
N	-2.80947731860880	-0.00634273674841	1.92049238247127
C	0.12445117611576	1.86081329974658	2.07033335540486
N	0.75374783130417	2.45704527010318	2.83530363451510
H	4.50663253627544	1.99993646634824	0.09662678892619
H	5.44575987720141	-0.29655352063286	0.06578244734916
H	3.91521762967032	-2.25097452653571	-0.01869689235439
H	1.46536742257901	-1.90336750676271	-0.06763921010973
H	2.08220550297788	2.35165277839378	0.04328563002386
H	-0.39760362743340	-0.54401202476134	-0.30149101185740
H	-0.04694349306742	2.53965406381150	-0.51546122843698
H	-1.54894597311259	1.54360098620228	-0.89030931203103
O	-1.22440818132119	-2.59875392896609	-0.91626180203474
C	-2.33786226490208	-2.58495771406564	-1.39114640895162
C	-3.56563896213065	-2.13308453273683	-0.65062471908077
S	-3.98481206607441	-0.45149830003251	-1.29859110036940
H	-2.50735851873950	-2.88144165536491	-2.45203295330822
H	-4.41424976864858	-2.80742631661570	-0.80149908009727
H	-3.35717106310824	-2.00239714224756	0.41408351261120
H	-4.30757544557597	-0.84910220871042	-2.54443984409066

1+2m⁻ G=-1084.582347E_h
28
Coordinates from ORCA-job starting E -1084.740341693953

C	3.67118122734038	1.07349604075400	-0.12276800702561
C	4.19425599622114	-0.21339469174196	-0.21272480192800
C	3.33361295223958	-1.30735058063878	-0.18282336551545
C	1.96245425847741	-1.11835009867586	-0.05454143426096
C	1.43019101397558	0.17311666983444	0.04375442357695
C	2.29975696104472	1.26704243120983	-0.00121825532814
C	-0.04223408688699	0.29597158826363	0.15851803816687
C	-0.69949423735019	1.14540574692482	1.30302440892240
C	-0.83170630555369	1.49807097433415	-0.18267012234238
C	-1.81528163206030	0.57334436914313	2.00338226835723
N	-2.68965801512608	0.11374955058119	2.60600138567786
C	0.13458744587577	1.97819432224430	2.11736679761651
N	0.81280580486876	2.66455697239221	2.75803428529437
H	4.33354266277950	1.93457059305483	-0.14854161024936
H	5.26643608553837	-0.36150863032326	-0.31082964901103
H	3.73021561751634	-2.31588832585362	-0.26451677229329
H	1.28037109426095	-1.96502933553681	-0.05419739857061
H	1.91276123729447	2.27922826566178	0.06657409151567

H	-0.57500002683028	-0.64656517417631	0.01834966334407
H	-0.31148576890100	2.40440517592882	-0.47917564352479
H	-1.80677777328294	1.28261867189368	-0.63634255952814
O	-1.07392225323377	-2.64638528524229	-0.56674163001705
C	-2.13310573312076	-2.57479631311194	-1.19653999682743
C	-3.34998065006569	-1.85219719921898	-0.75393259815913
S	-3.20507715596562	-0.41899949242711	-1.92159161999219
H	-2.21576952429220	-3.03403005298277	-2.20479210977104
H	-4.26807816141357	-2.42117782027417	-0.94627623455823
H	-3.29296803334988	-1.56330837201696	0.30019844643089

ts1 (trans) G=-1084.564367E_h ImF= 36.15i

28

Coordinates from ORCA-job ts12 E -1084.723028363198

C	-1.93346167519742	0.40414313079687	-0.00531636226747
C	-3.22768881522778	-0.07203862642611	-0.17288534352607
C	-3.97177753573421	-0.50115052244902	0.92447961598718
C	-3.40915962834569	-0.44886157212175	2.19533490583738
C	-2.11321864705901	0.02980244546419	2.36778065054412
C	-1.36186695259771	0.45924781026614	1.27209014317668
C	0.02546686979309	0.97948972582021	1.38779148891307
C	1.19905301493633	0.01692623441757	1.64690800837076
C	0.76940196424958	1.06932181680799	2.67483573533627
C	2.46423582397300	0.24837417010026	0.97334398138530
S	1.18738860061903	0.17073654815966	-1.71227601775236
N	3.59130073257475	0.50516470347350	0.83619023951436
C	2.48759846718621	1.38631745202452	-2.01481751495599
C	2.44062331902139	2.71923911182085	-1.33580394128349
H	3.29520403422468	3.38563909473516	-1.63598065962828
O	1.63234682200121	3.13073936151138	-0.52828671899772
C	0.89939351738367	-1.35175853085926	1.95129630481761
N	0.64067562572164	-2.44082015927986	2.24502945997185
H	-1.32254743002059	0.70853734964706	-0.85528665173648
H	-3.65516302236498	-0.11532257762629	-1.17137770954426
H	-4.98252504314440	-0.87793034584621	0.78935640956283
H	-3.97760649169611	-0.78419906644226	3.05897681858857
H	-1.69283252406490	0.06313045353793	3.36875719633046
H	0.31379603067750	1.68833056207562	0.61102212588431
H	1.46484769346719	1.89313522023225	2.79757598007271
H	0.32389434052463	0.68803577017911	3.58854203594452
H	3.47922685558731	0.99486768026003	-1.71146398178822
H	2.61119405351158	1.62230075972040	-3.08841919875769

A (trans) G= -1084.566676E_h

28

Coordinates from ORCA-job int1 E -1084.724502838463

C	-1.99164761899834	0.38014711347915	0.28685291749041
C	-3.29017067206572	-0.06370821739335	0.07589604969782
C	-4.08952912372140	-0.44182172402538	1.15378828430044
C	-3.57818275913603	-0.37376340714905	2.44525947744934
C	-2.27543208833252	0.07050933965272	2.65866624964417
C	-1.47072704617274	0.45052713792035	1.58468619155947
C	-0.07791778572381	0.93736943756156	1.76247183962771
C	1.11531955376644	0.01210960034668	1.49324104702701
C	0.80817931736208	0.51970039447120	2.89174626948262
C	2.23805747389617	0.65162358486210	0.73938427643992
S	1.66515309445688	0.32614811882532	-1.34293994606271

N	3.17185976749234	1.33906173294552	1.04461328806320
C	2.95702783111396	1.45571251493586	-1.87609137473390
C	2.56590395434892	2.75185939126963	-2.50359822244172
H	3.44927435309291	3.28659030161441	-2.95143041076597
O	1.47359396148126	3.27110380024673	-2.55100955250419
C	0.82841063021910	-1.36891444551828	1.23868831476559
N	0.59520371509904	-2.49294672917493	1.08337487497742
H	-1.35083563788618	0.65826427614394	-0.54818276171411
H	-3.67853244599434	-0.12362724526311	-0.93729097371571
H	-5.10419599476807	-0.79324353009558	0.98548968511940
H	-4.19332463099671	-0.66870343124787	3.29164030267629
H	-1.88035920594523	0.12678638301328	3.66953095323247
H	0.11736456509943	1.92422347646613	1.34533992494077
H	1.53038553642014	1.23541066542363	3.27055621520872
H	0.42954377935926	-0.21164737097150	3.59960739017735
H	3.50450871122027	1.73127790379011	-0.92799735315968
H	3.70886876531290	0.98135092787067	-2.52489295678219

ts2 (trans) G= -1084.564264E_h ImF= 64.43i

28

Coordinates from ORCA-job ts22 E -1084.724169305486

C	-1.85583356681996	0.33858033101530	0.11705082377413
C	-3.14584646857121	-0.12722936476035	-0.09886492488054
C	-3.98004635227265	-0.42155573206487	0.97913285812135
C	-3.51179129180046	-0.24854249412717	2.27717944384135
C	-2.21699506655295	0.21595236130161	2.49698503712573
C	-1.37873418603518	0.51238850934562	1.42252596509034
C	0.00858855662154	1.01250041190745	1.59562282757163
C	1.19717156735216	0.04342919437496	1.51210977810044
C	0.84434724428125	0.75798582037242	2.80804291495775
C	2.33048391441861	0.56937779872748	0.70842306082206
S	1.71313093048448	-0.13604786190296	-1.38763111530203
N	3.21508536303437	1.35411443360734	0.85030564426124
C	2.71363864990101	1.24355960762842	-1.98932692981693
C	2.31877790478700	2.62180796363146	-1.52024335050540
H	3.15842618834380	3.36064013675858	-1.57892396973871
O	1.21484163201743	2.99165391248941	-1.18306310427581
C	0.90609136147540	-1.35791864516623	1.44817830943789
N	0.66872029529387	-2.49143272108241	1.45192517949097
H	-1.18182007792220	0.54829883769580	-0.71227194241423
H	-3.50100905342354	-0.26984185684899	-1.11614752999686
H	-4.98838936173320	-0.78912019175552	0.80659404368764
H	-4.15415609040224	-0.47717467558348	3.12390499949355
H	-1.85746617244573	0.35442154914298	3.51319508209833
H	0.23603069134051	1.91879173009780	1.03482404541520
H	1.55728545459197	1.51915392436916	3.10707479132341
H	0.43332097831152	0.13973059987536	3.60068004905038
H	3.76926145139734	1.10876266124643	-1.71854785915680
H	2.66468650432707	1.27911375970434	-3.09133612757613

B (trans) G= -1084.564634E_h

28

Coordinates from ORCA-job prodottotrans E -1084.728546675722

C	3.87058293632579	1.22158875771859	0.09895027956722
C	4.47913682904368	0.02126780109507	-0.25615097384296
C	3.68518744410001	-1.07651949625547	-0.57511725544701

C	2.30021711172784	-0.96936740481122	-0.53897039763242
C	1.67569787992490	0.23436846779092	-0.18878379980493
C	2.48509541068805	1.32978879294174	0.13056291322835
C	0.19523122324382	0.27673398343923	-0.17466594908133
C	-0.62068416826383	0.67319716127970	1.08963037822951
C	-0.60747427326682	1.53526406199130	-0.16240024315085
C	-1.84176638858425	-0.12016400698303	1.44408572455902
N	-2.19252002766673	-0.38450281475338	2.61713868998325
C	0.13332897509104	1.17800038116420	2.20253075687693
N	0.79527333284708	1.62604749154721	3.03920812001481
H	4.47854611186680	2.08310876125788	0.36130879508484
H	5.56241226336676	-0.05897295545670	-0.27871133541835
H	4.14517879048765	-2.02222529992665	-0.84949449112013
H	1.68298985915995	-1.83104271343549	-0.78115285835268
H	2.03665443579890	2.27228605256931	0.42691867381671
H	-0.27843877484847	-0.56392205943442	-0.67631187519748
H	-0.08066441412540	2.48180579186600	-0.09416106858540
H	-1.53532724673263	1.55252909498462	-0.72530254780034
O	-4.52713979482659	-0.03885931087992	2.95850345887078
C	-3.67369542243458	-0.95732357297315	2.69325232686766
C	-3.85116264234355	-1.67053325310377	1.26720309651958
S	-2.87548215191917	-0.73479878019381	0.04355745586442
H	-3.57103883933843	-1.79977946356231	3.43780627392176
H	-3.49810492171059	-2.71100847826731	1.27840308952754
H	-4.90963353761124	-1.62653598960911	0.99886476250149

4 (trans) G= -1085.125241E_h

29

Coordinates from ORCA-job protoprod E -1085.304547367195

C	3.83619052509794	1.19986199130346	0.12409192785650
C	4.42751216863709	-0.03992251658106	-0.09708435657034
C	3.62703092198389	-1.14442790896732	-0.37116203721783
C	2.24564046263432	-1.00629215870778	-0.42105490861401
C	1.64209860887937	0.23664708802947	-0.20296119382787
C	2.45465207483978	1.34005772348458	0.06954785107391
C	0.16237203380910	0.31337229799305	-0.26652852898151
C	-0.68049700600079	0.76756791242636	0.97598534563368
C	-0.62120535644490	1.57334662918031	-0.31364964217768
C	-1.88008219108881	-0.03347764866664	1.31874428924339
N	-2.08918243901227	-0.49657669112250	2.48514026144740
C	0.04181694650359	1.29530824981737	2.09713610946695
N	0.64406094620255	1.76257219552183	2.96612224744720
H	4.45300198050208	2.06608618942965	0.34351942529925
H	5.50747184874465	-0.14480500686722	-0.05451737865043
H	4.07848874805153	-2.11668760203526	-0.54536045852122
H	1.62280601887435	-1.87218666772522	-0.63207927185757
H	2.01837911799593	2.31667252813277	0.25382678426186
H	-0.30653844111233	-0.53634939481210	-0.75723482989467
H	-0.09198447757418	2.51782441704405	-0.24898353979783
H	-1.52371606850366	1.59749710541969	-0.91674881159034
O	-4.31115674791966	-0.32175290535513	3.25888436074307
C	-3.38440809595917	-1.16060973466594	2.59892912633486
C	-3.86047063929981	-1.58330299621425	1.18575962564329
S	-3.09248126785197	-0.38787809815515	0.02999561773623
H	-3.29197917839881	-2.04769768095236	3.23627214898349

H	-3.50757327256062	-2.58856253540239	0.93472586926426
H	-4.94958888456213	-1.54316454028436	1.11271717338451
H	-4.22990643706710	0.56610800403210	2.88618246608143

ts1 (cis) G= -1084.560395E_h ImF= 61.12i

28

Coordinates from ORCA-job ts1 E -1084.719065353482

C	-2.11787185631412	0.09943711155405	0.95110599324810
C	-2.56546422200282	-0.51945843980290	2.11647633494278
C	-1.83249984406672	-0.38610485259450	3.29172221252809
C	-0.65737888975929	0.36019987666620	3.29776525650639
C	-0.20863964049401	0.98633025288705	2.13504819297981
C	-0.95071428278745	0.85455972971891	0.95697235141584
C	1.03059854285112	1.81445205713496	2.14413908149517
C	2.26669520436402	1.36684164927232	1.34920857817040
C	2.26316018495180	1.38827941933660	2.87552213706252
C	2.20174504631199	0.04802285770573	0.68533198376757
S	1.62500173848269	0.77794975537492	-1.52217896543419
N	2.22088287879505	-1.10398672439712	0.96379936483735
C	1.04748102476627	-0.91141603526450	-1.79940081636572
C	0.29727968685538	-1.23683451372159	-3.04570376237224
H	0.03944274813520	-2.33065130646236	-3.10416561844510
O	-0.05151876503803	-0.49562529702497	-3.93770454177974
C	3.00952358414940	2.42126791781510	0.71575532732636
N	3.63023749497863	3.31404523112872	0.31762319868838
H	-2.67386514496761	-0.01490532940391	0.02415488137850
H	-3.47650608148168	-1.11247862393635	2.10574898520251
H	-2.16857185394392	-0.87338426812297	4.20345117559797
H	-0.07542529896012	0.45365583511147	4.21142807125107
H	-0.56391552259922	1.29242980652630	0.03713825892324
H	0.86072978595883	2.88882592337597	2.08604915063217
H	2.86701608871172	2.14442056506284	3.36710264513515
H	2.26977544143059	0.39486976126268	3.31311873865680
H	1.88286499729214	-1.63085851753656	-1.74193113205064
H	0.39783595438014	-1.24718484166616	-0.96848008329865

A (cis) G= -1084.563218E_h

28

Coordinates from ORCA-job intermediocis E -1084.722278408205

C	-2.73025839893611	1.23650285615952	1.90639114246658
C	-2.82451440547534	-0.09809472847306	2.29110111858186
C	-1.66418623750117	-0.80202514712134	2.59915852065773
C	-0.41913732900875	-0.18540208656546	2.53494400245984
C	-0.31389435959767	1.15561313993335	2.14941907834811
C	-1.48669265942108	1.85182228910137	1.83359842019281
C	0.97500935547838	1.88027402904238	2.05467517630294
C	2.12751212591686	1.38432977911953	1.13278428486266
C	2.27150607750022	1.40519880651474	2.63537482246875
C	1.96604441390344	0.04448923578322	0.46771847659046
S	1.22870968684802	0.43726867331000	-1.42671278144184
N	2.10326021222723	-1.07448863274636	0.90781648166274
C	0.92447617113555	-1.31324401884220	-1.69596257560942
C	1.53147533995425	-1.98125959833354	-2.87760733802802
H	1.12254153688349	-3.01974039851888	-3.02313090138555
O	2.38196946664042	-1.56784288025322	-3.63455606870136
C	2.79257507539436	2.43349291931203	0.41610744231029

N	3.34209398683483	3.31519225666174	-0.09837735597003
H	-3.62583291595394	1.79743859379801	1.65183417668027
H	-3.79379838054500	-0.58715714403573	2.34291140666011
H	-1.72280893531047	-1.84873631115463	2.88582309396144
H	0.47737647315412	-0.76057613893578	2.73884481510413
H	-1.41566641906890	2.88906534646808	1.51421697924030
H	0.85700747757547	2.96134338187026	2.01890294983055
H	2.92796124446852	2.14996516215206	3.07467836295313
H	2.30853331438844	0.40994525105981	3.06794717985063
H	1.37107942833243	-1.79731889124634	-0.76987837775466
H	-0.14444134581761	-1.57335574405960	-1.66892253229445

ts2 (cis) G= -1084.559414E$_h$ ImF= 103.81i

28

Coordinates from ORCA-job ts2 E -1084.719173629287

C	-2.70528552427806	1.22483565363214	1.98417338325565
C	-2.79539782263733	-0.11690210211332	2.34415956865566
C	-1.63023957465230	-0.83307455028167	2.60166261769522
C	-0.38440264468759	-0.22121995317631	2.51157673769951
C	-0.28339985328138	1.12684007743427	2.15080399541247
C	-1.46122867468458	1.83525050033540	1.88559903432219
C	1.00669741272081	1.84481359773468	2.02701008523179
C	2.11738764998648	1.35947253198306	1.05189191687917
C	2.31965314792670	1.34529131159414	2.54867219831704
C	1.91208292737787	0.03980146981455	0.36733927738157
S	1.03667549688740	0.57542535433138	-1.51012419919574
N	2.05103114759165	-1.09563282457744	0.73799128124963
C	0.67879747008632	-1.16063011735111	-1.83737945718935
C	1.76153846199935	-1.99666992088873	-2.46616284440580
H	1.56019193013766	-3.09725385743933	-2.36179136753869
O	2.73970898863298	-1.61977494390741	-3.06512326951009
C	2.76958806055287	2.41642427941052	0.33439178346755
N	3.31706398341886	3.30493855126671	-0.17003760012504
H	-3.60499003731781	1.79508782654660	1.76782604464613
H	-3.76538537020868	-0.60214937165527	2.41521010655309
H	-1.68591215243417	-1.88525921008616	2.86827666438805
H	0.51412140189402	-0.80559156882660	2.67677362259705
H	-1.39366219030530	2.87765651504806	1.58304229206116
H	0.89690149904830	2.92723775439582	2.01969151797316
H	2.99983070354093	2.07437562300444	2.97811768498045
H	2.36291305210891	0.34176556699900	2.96066367634350
H	0.43651499075859	-1.65650907340853	-0.88278226137368
H	-0.20289448018250	-1.25585111981889	-2.49237348977164

B (cis) G= -1084.568156E$_h$

28

Coordinates from ORCA-job .\prodottocis E -1084.733710246858

C	1.72969689090380	-1.28130432108980	-3.82822769149407
C	2.07524658765895	-2.39262755544469	-3.06413080735023
C	1.55322029880977	-2.53062550936134	-1.78148028878245
C	0.67634561563625	-1.58570385015287	-1.26109548509344
C	0.33010682531171	-0.46154368265824	-2.01815315099634
C	0.87127102265036	-0.32182868074959	-3.30234057993859
C	-0.59741162792438	0.59944715209614	-1.54738276084672
C	-0.69712753129777	1.09472970455636	-0.08845834700466
C	-1.83149997078349	0.32443020459536	-0.73656934758040
C	0.06832174776925	0.44054590112527	1.02591911621282

S	-0.95537778517602	-0.20335747874811	2.39987927970620
N	1.31285998442856	0.27813297671081	1.05997812221746
C	0.59591352538492	-0.62402142794507	3.26177412199286
C	1.70917787135523	-0.80047757585258	2.13981919198965
H	2.66407786073551	-0.40191873250554	2.58101926803413
O	1.76272237069212	-1.97540300366955	1.59775662620560
C	-0.89159352917330	2.50952219013583	0.04944649532357
N	-1.06635051337221	3.65243029026809	0.13148966406202
H	2.13746026783987	-1.15210410876079	-4.82785947681527
H	2.76325386506551	-3.13590146322656	-3.45951313508779
H	1.84710591320414	-3.35892704022401	-1.14421134093238
H	0.35173745519536	-1.72203796110313	-0.23314437311507
H	0.61776341345676	0.55532476122242	-3.89481361909622
H	-0.70590255601960	1.41525017843892	-2.25967423955003
H	-2.74391984973272	0.86021358690726	-0.97933321890090
H	-1.95575408398051	-0.68581535497081	-0.35735665752948
H	0.84193587869838	0.18367978942648	3.96501638546817
H	0.48392005266355	-1.57340898902027	3.79204624890156

4 (cis) G= -1085.12546E_h

29

Coordinates from ORCA-job .\prodottocisprotonato E -1085.304809803443

C	1.91473664987276	-1.66249710111331	-2.54294859814039
C	1.30493117125199	-2.68164622412466	-3.27069998262321
C	-0.03681160760739	-2.57418322421989	-3.62113487044423
C	-0.77032560219196	-1.45618769658027	-3.23708807492821
C	-0.16624090875736	-0.43635901903945	-2.50257686394314
C	1.18516856170845	-0.54298283572903	-2.16581464996147
C	-0.93899202880217	0.76997813773037	-2.09561119247684
C	-1.22049296005079	1.05121693300930	-0.59553816133255
C	-2.32971264142354	0.69349704891362	-1.57772388561633
C	-0.75573127326795	0.08922026580408	0.44212979681335
S	-1.27703773399563	-1.61809776561901	0.39520963913956
N	0.02446713824821	0.46051146267125	1.37888041714727
C	-0.44448388578184	-1.87019356599038	2.00996879347936
C	0.47593822666762	-0.65223747947522	2.20303243278391
H	0.47584657710726	-0.33764667148869	3.25841169532675
O	1.78778428301300	-1.03514368493973	1.80629763503556
C	-1.12731081158293	2.43288537104644	-0.21182025097143
N	-1.09044173581170	3.56190699132017	0.03537424266960
H	2.96027354920620	-1.74481988671344	-2.26145121317271
H	1.87529603624957	-3.55864787099255	-3.56237756281840
H	-0.51648598058722	-3.36469009474479	-4.19087804206212
H	-1.82034342169254	-1.37462432493833	-3.50617427705467
H	1.65395744609810	0.24637480014750	-1.58346918716263
H	-0.68415926983061	1.68342164418548	-2.63120992414551
H	-3.02701124168174	1.48685190193130	-1.82416702658141
H	-2.77176690090239	-0.28919944524461	-1.43979159671952
H	-1.20610712850308	-1.92022247742593	2.79267146695164
H	0.13800346769838	-2.79444848122774	1.99562046166774
H	2.31345202534930	-0.22623670715242	1.75647877914007

7a - trans (NOE)

30

Coordinates from ORCA-job aminatrans E -1011.309832876548

C	-3.52151776925232	-0.20649442652003	-1.28180260971047
C	-4.29666367479350	-0.56483013720250	-0.18104678719807
C	-3.85578963467097	-0.24421506897791	1.09843369072241
C	-2.64633732638129	0.42080513773952	1.28103488420049
C	-1.85083702231041	0.77107838981230	0.18704593379306
C	-2.31370268570052	0.45453687683422	-1.09843233727767
C	-0.53241189020286	1.44246081113213	0.31711869539984
C	0.73522826912316	0.63116490700634	0.68323756205114
C	0.17813235380596	1.67826682163629	1.61182174584288
C	1.95481339980967	0.99039898182350	-0.07367066873880
S	2.85980546377253	-0.17171527822548	-1.04373121262613
C	3.96491106387897	1.09827994749640	-1.46258158239546
C	3.56939749494740	2.26867612800537	-0.89002181872348
N	2.44466529865231	2.20105152997884	-0.10919516801246
C	0.55953809325222	-0.82018691467027	1.07344927020102
N	0.43306900309267	-1.65950385389151	-0.12176570073131
H	-3.86038837883079	-0.44200546937053	-2.28676964588800
H	-5.24136745178561	-1.08164432504924	-0.32140857937048
H	-4.46026861382974	-0.50488800149370	1.96265922365811
H	-2.33009914491161	0.68285573731376	2.28626369534533
H	-1.70877299226301	0.73027316272791	-1.95973076189609
H	-0.32896555266327	2.18464339574162	-0.45222876747451
H	0.74344467772599	2.59894186777099	1.70344379912060
H	-0.27359407648409	1.31052430697078	2.52844798252461
H	4.81815962442572	0.91824537893238	-2.10280907527974
H	4.07931244023934	3.21948959719660	-1.00349735444917
H	1.44970950170537	-1.14121062403366	1.63203573864983
H	-0.29822571163724	-0.89412210380720	1.76208034026088
H	0.43550624502080	-2.64120756742800	0.13103690422914
H	-0.45725100373485	-1.47046920744893	-0.57321739622750

7a - cis (NOE)

30

Coordinates from ORCA-job .\aminacis E -1011.307427901529

C	-2.59117499990190	0.65407458920978	-0.33266307141924
C	-3.11346416083502	0.14420982347288	0.85475162955982
C	-2.37294261003252	0.23523867678505	2.02826725026347
C	-1.11286367333105	0.82792399218710	2.01487218660704
C	-0.58061760238774	1.33367028488574	0.82941962541356
C	-1.33474303114590	1.24568429378779	-0.34511821342465
C	0.76357269881156	1.97022647780319	0.78163623182773
C	1.94754021077638	1.29986311234264	0.07729962243149
C	1.92085854344331	1.46611002801712	1.58249624706641
C	1.78603040491914	-0.04649094818444	-0.50907632872984
S	1.15604400506099	-1.43494687197290	0.37886295400420
C	1.35279048775476	-2.40000288385706	-1.04429473281027
C	1.85831151700593	-1.63791405425280	-2.05320787405417
N	2.09984938200117	-0.32404204338996	-1.74736279354643
C	2.83573542687598	2.23089193206189	-0.72976198451517
N	2.15048798929963	2.69066033007748	-1.94549846736545
H	-3.16419510721636	0.58390747081866	-1.25296482529170
H	-4.09433214899853	-0.32241511795883	0.86325195504622
H	-2.77543292251109	-0.15720800555653	2.95791235486527
H	-0.53564819180179	0.89875126060358	2.93337174274334
H	-0.91214652615658	1.63387055119590	-1.26911743670911

H	0.74161432714385	3.04182842914494	0.58452977491201
H	2.61473022001265	2.18176039864009	2.01412319480091
H	1.77438719157176	0.56775174888636	2.17683313806469
H	1.08153050100718	-3.44665488393348	-1.05837669924662
H	2.07363506150128	-2.00026679741280	-3.05263553030552
H	3.05310502673200	3.10838442637457	-0.10702425417224
H	3.79401570751531	1.71745370218515	-0.92057118628717
H	2.72137088302445	3.38913261624745	-2.41006222794757
H	2.08325138986110	1.90164746179146	-2.58219228178102

5a

26

Coordinates from ORCA-job prodottodisidratato E -1008.888927789579

C	-2.20461383424462	-3.45007016327404	0.33051336423305
C	-3.34815682136572	-2.68026990405421	0.14891923824834
C	-3.27258128774588	-1.29342441564892	0.25664139061885
C	-2.05923696021558	-0.68279334340147	0.54306640278278
C	-0.90379294288450	-1.44981155807608	0.72170275917252
C	-0.98742779518229	-2.83782916776114	0.61492070009310
C	0.38097184551379	-0.76843053620154	1.03479638335539
C	1.15806812808795	-0.03640440298943	-0.09085835971941
C	1.69513530475040	-1.29398474661646	0.58971860433143
C	1.78493859677473	1.26257535484066	0.24521982193613
S	1.78960145936096	2.61617216359669	-0.86947083058166
C	2.61908292029127	3.54666280607351	0.33331567520774
C	2.82263703983177	2.78955997194848	1.44825566971225
N	2.35136263089570	1.50328301448103	1.39336179040281
C	0.65315653468525	-0.17231098689896	-1.42440940789678
N	0.23573334325019	-0.26575229353510	-2.49940999790095
H	-2.25775451557146	-4.53189509666386	0.25052686174157
H	-4.29644878354657	-3.15817218843640	-0.07853160100436
H	-4.16132059690022	-0.68666660364135	0.11067304324454
H	-1.99657767221412	0.40038838286593	0.61645121663614
H	-0.09907523529745	-3.44503523815067	0.76495407959716
H	0.40181640705416	-0.18059227342636	1.95126037632070
H	2.55686921324821	-1.12596431115944	1.22700107435387
H	1.71211021713906	-2.18548917420161	-0.02882013149108
H	2.90887141863766	4.57254937740223	0.15143863322879
H	3.32333138564730	3.13550533292850	2.34596324337709
